# Prevalence of co-morbid anxiety and depression in pregnancy and postpartum: a systematic review and meta-analysis

**DOI:** 10.1017/S0033291725000601

**Published:** 2025-03-13

**Authors:** Lu Ou, Quan Shen, Meili Xiao, Weihong Wang, Tan He, Binglu Wang

**Affiliations:** 1 Department of Basic Nursing, School of Nursing, Hunan Normal University, Changsha, China; 2 Department of Clinical Nursing, Xiangya Nursing School of Central South University, Changsha, China

**Keywords:** co-morbid anxiety and depression, meta-analysis, perinatal, pregnancy, prevalence, postpartum

## Abstract

The prevalence of co-morbid anxiety and depression varies greatly between research studies, making it difficult to understand and estimate the magnitude of this problem. This systematic review and meta-analysis aim to provide up-to-date information on the global prevalence of co-morbid anxiety and depression in pregnant and postpartum women and to further investigate the sources of heterogeneity. Systematic searches of eight electronic databases were conducted for original studies published from inception to December 10, 2024. We selected studies that directly reported prevalence data on co-morbid anxiety and depression during the perinatal periods. We extracted data from published study reports and calculated the pooled prevalence of symptoms of co-morbid anxiety and depression. There are 122 articles involving 560,736 women from 43 different countries included in this review. The global prevalence of co-morbid anxiety and depression during the perinatal period was about 9% (95%CI 8%–10%), with approximately 9% (95%CI 8%–11%) in pregnant women and 8% (95%CI 7%–10%) in postpartum women. Prevalence varied significantly by the assessment time points, study country, study design, and the assessment tool used for anxiety and depression, while prevalence was not dependent on publication year, country income level, and COVID-19 context. No publication bias was observed for this prevalence rate. These findings suggest that approximately 1 in 10 women experience co-morbid anxiety and depression during pregnancy and postpartum. Targeted action is needed to reduce this burden.

## Introduction

Anxiety and depression are common mental health problems in pregnant and postpartum women and frequently co-occur (Biaggi, Conroy, Pawlby, & Pariante, 2016; Smythe, Petersen, & Schartau, 2022). It is widely known that the maternal and child outcomes of co-morbid anxiety and depression (CAD) are more negative than each condition alone (Spijker, Muntingh, & Batelaan, 2020). For women, comorbidity is associated with non-physiological delivery, recurrent mental illness, and even suicide (Hirschfeld, 2001; Vichi, Berardelli, & Pompili, 2021; Hulsbosch et al., 2023). For the offspring, adverse outcomes include preterm birth, low birth weight, poor infant cognitive development, and mental health problems in late childhood (Field et al., 2010; Yang et al., 2017; Uguz, Yakut, Aydogan, Bayman, & Gezginc, 2019). Furthermore, CAD is associated with a high disease burden and economic cost (e.g. lengths of hospital stay, hospital transfers, and delivery-related costs) (Bitew, Hanlon, Kebede, Medhin, & Fekadu, 2016; McKee et al., 2020). Therefore, a better understanding of the prevalence of CAD in pregnant and postpartum women is crucial for the monitoring and management of this problem.

Currently, numerous studies have reported symptoms of CAD in pregnant or postpartum women, however, prevalence rates vary greatly, ranging from 5% to 73% (Abdelhai & Mosleh, 2015; Premji et al., 2020). Many studies have demonstrated that the methods- and sample-related variables, including study design, publication year, study country, assessment tool, and assessment time-points predict the prevalence rate variation (Canuto, Weber, Baertschi, Andreas, & Härter, 2018; Xiao, Hu, Huang, Wang, & Lei, 2022; Mohamadi, Ahmadzad-Asl, Nejadghaderi, Jabbarinejad, & Davoudi, 2023). A previous systematic review has examined some of these issues and assessed the variation across different time points (1st, 2nd, and 3rd trimester of pregnancy, or 1–4, 5–12, 1–24, and >24 weeks of postpartum), measures (self-reported, clinical diagnosis), county income levels (low to middle income, high income), and the publication year (<2010 and >2011) (Falah-Hassani, Shiri, & Dennis, 2017); however, the literature search used only included studies published up to January 2016 and have not assessed the variations in other important moderating factors, such as assessment tools, and COVID-19 context. For example, Allegri et al. (2023) reported that the COVID-19 epidemic and related restrictions increase anxiety-depressive symptoms in pregnant women; Reck et al. (2013) showed that assessment of anxiety using a general anxiety scale (State–Trait Anxiety Inventory), rather than using pregnancy-specific anxiety scale (Pregnancy-Related Anxiety Questionnaire), underestimate anxiety level during pregnancy.

Despite establishing precise prevalence estimates of CAD is a key first step in understanding the burden experienced by women in the perinatal periods and addressing this important public health issue (Ogunyemi et al., 2018), a comprehensive evaluation of the estimated prevalence of CAD in pregnancy and postpartum is still lacking. To address this gap, the current systematic review and meta-analysis aims to: (i) synthesize the newest evidence to provide the global prevalence of CAD among pregnant and postpartum women, and (ii) investigate whether prevalence varies based on publication year, country, study design, COVID-19 context, time points, and the assessment tools for anxiety or depression, most of which were not performed in previous review (Falah-Hassani et al., 2017).

## Methods

### Design

This systematic review was conducted following the Centre for Reviews and Dissemination guidelines (Tacconelli, 2010), and reported in accordance with the Preferred Reporting Items for Systematic Review and Meta-analysis (PRISMA) statement (Page et al., 2021). This review protocol was registered on PROSPERO on November 20, 2023, with the registration number CRD42023480485. This review contained no deviations from the pre-registered protocol.

### Search strategy

We systematically searched the literature in the seven electronic databases (PubMed, Web of Science, EMBASE, the Cochrane Library, PsycINFO, CINAHL, and Scopus) from inception to December 10, 2024. Additionally, we hand-searched the reference lists of the previous review (Falah-Hassani et al., 2017) and included articles, and we also consulted a grey literature database (Google Scholar) to identify other potentially relevant studies. The full search strategy in the above databases is available in Supplementary Table S1.

### Study selection

The study selection was conducted based on a three-step screening process: literature retrieval, preliminary review, and full-text review. Literature retrieval was independently conducted by four reviewers (OL, SQ, WBL, and HT) and all selected articles were exported into EndNote X20 (2020). Subsequently, the same reviewers preliminarily filtered the selected articles by reviewing titles and abstracts after identifying duplication. Finally, full texts were retrieved and reviewed following the eligibility criteria. The overall kappa selection between the two reviewers was 0.83. Any disagreements were resolved through consulting the other reviewer (XML).

Inclusion criteria were studies: (1) assessed for anxiety and depression using validated diagnostic criteria or self-report questionnaire in the perinatal period (pregnancy and after birth within 1 year); (2) directly reported the prevalence of CAD in the full-text; (3) we included cross-sectional studies, longitudinal studies, cohort studies, clinical trial studies, case report and case series studies; however, for the latter three study designs, we only included data from the recruitment phase (selecting individuals with CAD from a large population); and (4) written in English language. Exclusion criteria were: (1) unavailable data on the prevalence of CAD after contacting the corresponding authors and (2) abstract, review, case report, comment, letter, and protocol.

### Data extraction

Two reviewers (OL and SQ) independently extracted data using a unified excel table from the eligible studies as follows: name of the first author, publication year, study country, study design, study setting, sample size, women’s characteristics, assessment tools of anxiety and depression, time point of measuring anxiety and depression, and the prevalence of CAD. The data extracted to calculate the prevalence of CAD were the number of women who were identified as CAD divided by the total number of women in the perinatal period. Any disagreement was resolved by discussion and consulting the third reviewer (XML).

Time points of assessment were defined as the two stages (pregnancy and postpartum) with the following nine time points: first trimester (1–12 gestational weeks), second trimester (13–28 gestational weeks), third trimester (29–40 gestational weeks), as well as, 1–7 days postpartum, 1 week to 1 month postpartum, 1 to 2 months postpartum, 2 to 4 months postpartum, 4 to 6 months postpartum, 6 to 12 months postpartum. If data were missing or in a format that could not be extracted, reviewers would attempt to contact the corresponding author for the relevant data.

For a randomized controlled trial that reported the prevalence of CAD, we just extracted the baseline data regarding the identified CAD case and total sample size. For longitudinal studies that measured CAD at several time points, we extracted the identified case and sample size at a single time point and incorporated them into similar or same time points. For case-control studies, we just used the data during the early case screening stage.

### Risk of bias assessment

Two reviewers (OL and SQ) independently used a tool to assess the risk of bias in all included studies developed by Hoy et al. (2012), which was designed specifically for prevalence studies and has been widely used in assessing the risk bias of prevalence studies (Migliavaca, Stein, Colpani, Munn, & Falavigna, 2020). This tool consists of 10 domains, and each included study underwent scrutiny across these domains involving (1) population representation; (2) sampling frame; (3) participant selection procedures; (4) non-response bias; (5) direct data collection from subjects; (6) acceptability of case definition; (7) reliability and validity of study instrument; (8) data collection mode; (9) data collection length; and (10) appropriate description of numerator and denominator. There were two categories for each item: low bias risk (1) and high bias risk (0). The total score ranged from 0 to 10, and the overall bias risk score was graded on the basis of how many studies have a high risk of bias: low (8–10), moderate (6–7), and high (0–5) (Santiago, Oliveira, Silva, Silva, & Villela, 2023). The overall kappa selection between the two reviewers was 0.86. Any disagreements were discussed and consulted with a third one (XML).

### Data analysis

The meta-analysis was performed using the ‘meta’ package of R Software Version 4.3.2. Statistical tests used 2-tailed *p*-value of <0.05 for significance. A random-effects model was used to pool the overall prevalence estimates and 95% confidence intervals (95% CI) since the data were not similar (e.g. inconsistent assessment tools for anxiety and depression) in the included studies. All prevalence rates were tested for normality, and the log transformation was used to correct for non-normally distributed raw estimates (Lipsey & Wilson, 2000; Higgins, White, & Anzures-Cabrera, 2008; Cheung, 2019). We presented the overall prevalence of CAD with 95% CI in the forest plot, as well as the prevalence by subgroups, defined prior by publication year (before 2016, after 2016), country income level (high-income or low- and middle-income country), study design (cohort study, cross-sectional study, longitudinal study, prospective cohort study, quasi-experimental study, retrospective cohort study, secondary analysis of RCT, case–control study), risk of bias (low-risk, moderate-risk, high-risk), COVID-19 context (whether yes or no), CAD disorders (whether diagnosed as CAD disorders by doctors), assessment tools for anxiety (e.g. STAI, GAD-7, and HADS), assessment tools for depression (e.g. EPDS, HADS, and PHQ-9), and different time points in the perinatal periods (pregnancy, postpartum). Meanwhile, the year 2016 was chosen as the time point since a recent relevant review retrieved data prior to 2016 (Falah-Hassani et al., 2017). The country income classification was based on the World Bank Country and Lending Groups (The World Bank Group, 2020). Further, to clearly present the global prevalence of CAD in different countries, we also synthesized the prevalence of the different countries and drew a world map with the use of ‘map’ package. Heterogeneity was quantified using the Q test combined with *I*^2^ statistics in each pooled analysis. Heterogeneity was not regarded as important if *I*^2^ was <50% or the *p-*value of the *Q* test was >0.1 (Higgins, Thompson, Deeks, & Altman, 2003; Borenstein, Hedges, Higgins, Rothstein, & 2009).

Meta-regression was performed with Restricted Maximum Likelihood methods to test the source of heterogeneity, which could determine if prevalence estimates were conditional on certain moderators. The potential moderators for the prevalence of CAD were publication year, study design, COVID-19 pandemic context, study countries, assessment tools for anxiety and depression, and time-point of assessment, according to previous studies (Canuto et al., 2018; González-Mesa et al., 2020; Luo, Xue, Ma, & Liu, 2021; Xiao et al., 2022; Mohamadi, et al., 2023; Hannon et al., 2023). Additionally, to determine the robustness of the observed outcomes in performing the meta-analysis, sensitivity analyses were performed to explore the impact of an individual study on the overall result following the sequential exclusion of each study (Higgins et al., 2023). Publication bias was estimated using a funnel plot and Egger’s test. If the *p*-value of Egger’s test was more than 0.05 and funnel plots were symmetrical, it indicated that there was no obvious publication bias in this meta-analysis (Egger, Davey Smith, Schneider, & Minder, 1997).

## Results

### Search results

**Figure 1.**
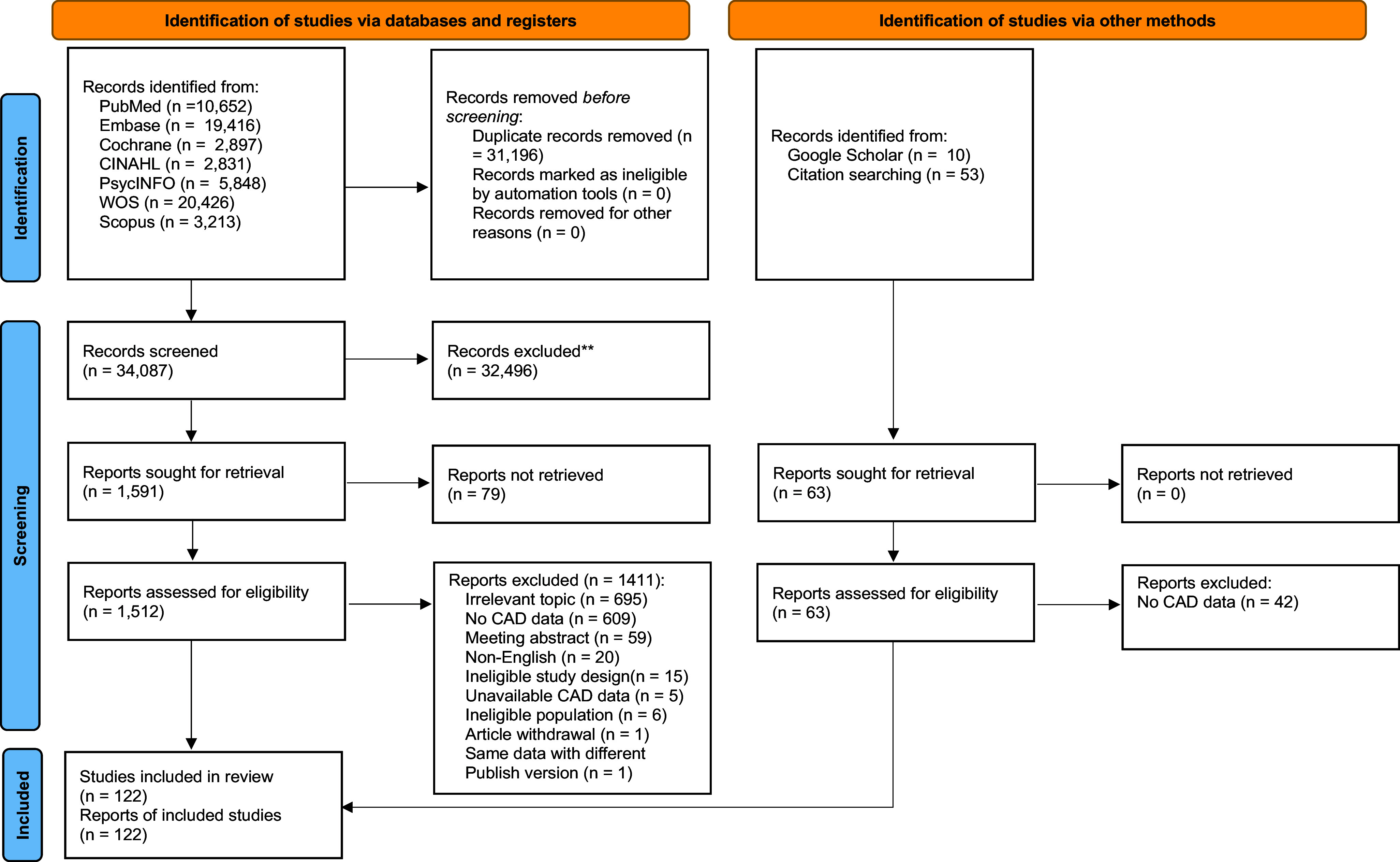
Flow chart of the search strategy and selection of studies.

The literature research initially yielded 65,283 potentially relevant studies. After removing duplicates, there were 34,087 studies left. Of these 34,087 studies, 32,496 were removed after the screening of titles and abstracts, and 1,411 studies remained for further full-text reading (the reasons for exclusion were presented in Figure 1). Finally, 122 articles met the inclusion criteria and were eventually included in this systematic review and meta-analysis, involving three clinical trial studies, two case–control studies, and 117 observational studies (31 cohort studies, 23 longitudinal studies, and 63 cross-sectional studies).

### Characteristics of the included studies

The included studies were conducted in 43 different countries covering six continents, most of which were conducted in Asia (*n* = 45), Europe (*n* = 27), and Africa (*n* = 16). The others were conducted in North America (*n* = 24), South America (*n* = 6), and Australia/Oceania (*n* = 12). When stratified according to the study country, 67 studies were conducted in high-income countries, followed by middle-income countries (*n* = 43) and low-income countries (*n* = 12). The sample size ranged from 75 to 116,457, with a total of 560,736 participants. Regarding the assessment tool for anxiety and depression, the majority of the studies used a self-report questionnaire to assess anxiety and depression. For anxiety, most of the studies used the State–Trait Anxiety Inventory (STAI) (*n* = 21), followed by 7-item Generalized Anxiety Disorder (GAD-7, *n* = 18) and Hospital Anxiety and Depression Scale (HADS, *n* = 16). For depression, the Edinburgh Postnatal Depression Scale (EPDS) was the most used (*n* = 51), followed by the Hospital Anxiety and Depression Scale (HADS, *n* = 14) and 9-item Patient Health Questionnaire (PHQ-9, *n* = 11). Additionally, there were 23 articles performed in the COVID-19 context. The detailed characteristics of the included studies were presented in Supplementary Table S2.

### Quality assessment

In the included studies, 17 studies scored nine points, 74 scored eight points, 26 scored seven points, 5 scored six points based on Hoy’s tool. Thus, 31 studies (25.4%) showed moderate risk of bias, and 91 studies (74.6%) were rated as low risk of bias.

For the validity of study methods, 4% (*n* = 5) studies did not use the applicable study instrument, 20% (*n* = 25) studies had a lower response rate or did not describe refusers, and pregnant women in 6% (*n* = 7) studies belong to the specific population. 86% (*n* = 105) of studies had not a random sample. The detailed risk of bias assessments of the included studies was presented in the Supplementary Table S3.

### Prevalence of co-morbid anxiety and depression

**Figure 2.**
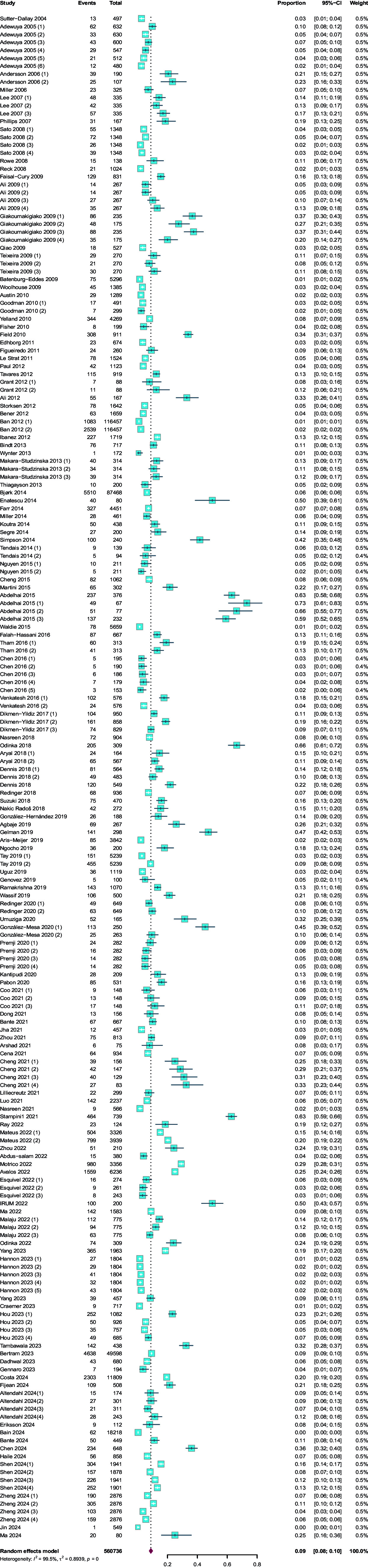
Overall prevalence rates of co-morbid anxiety and depression in pregnancy and postpartum

**Figure 3.**
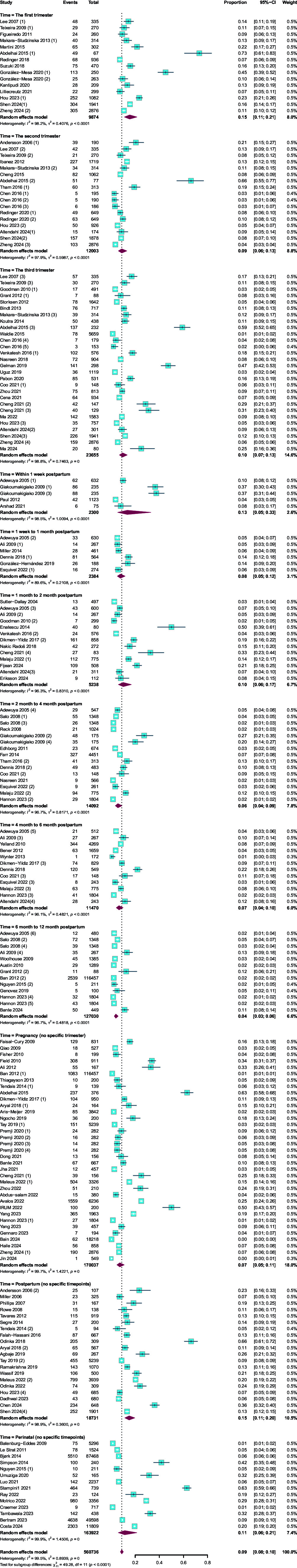
Overall prevalence rates of co-morbid anxiety and depression by assessment timepoints

The overall prevalence of CAD during pregnancy and postpartum was about 9% (95%CI 8%–10%, *I*^2^ = 99.5%, *n* = 122), with 9% (95%CI 8%–11%, *I*^2^ = 99.4%, *n* = 66) in pregnancy periods and 8% (95%CI 7%–10%, *I*^2^ = 99.3%, *n* = 59) in postpartum periods. The overall prevalence and the prevalence in different assessment time periods were illustrated in Figures 2 and 3, respectively. Prevalence estimated by time-points of the assessment in the pregnancy periods showed the highest rate of CAD of 15% in the first trimester (95%CI 11%–21%, *I*^2^ = 98.2%, *n* = 14). Similar rates of CAD were also observed in the second trimester (9%, 95%CI 6%–13%, *I*^2^ = 97.9%, *n* = 14) and third trimesters (10%, 95%CI 7%–13%, *I*^2^ = 98.9%, *n* = 26). Meanwhile, during postpartum periods, the highest rate of CAD was 13% (95%CI 5%–33%, I^2^ = 98.5%, n = 4) within 1 week postpartum, with a gradual decline trend during 1 year postpartum.

**Figure 4.**
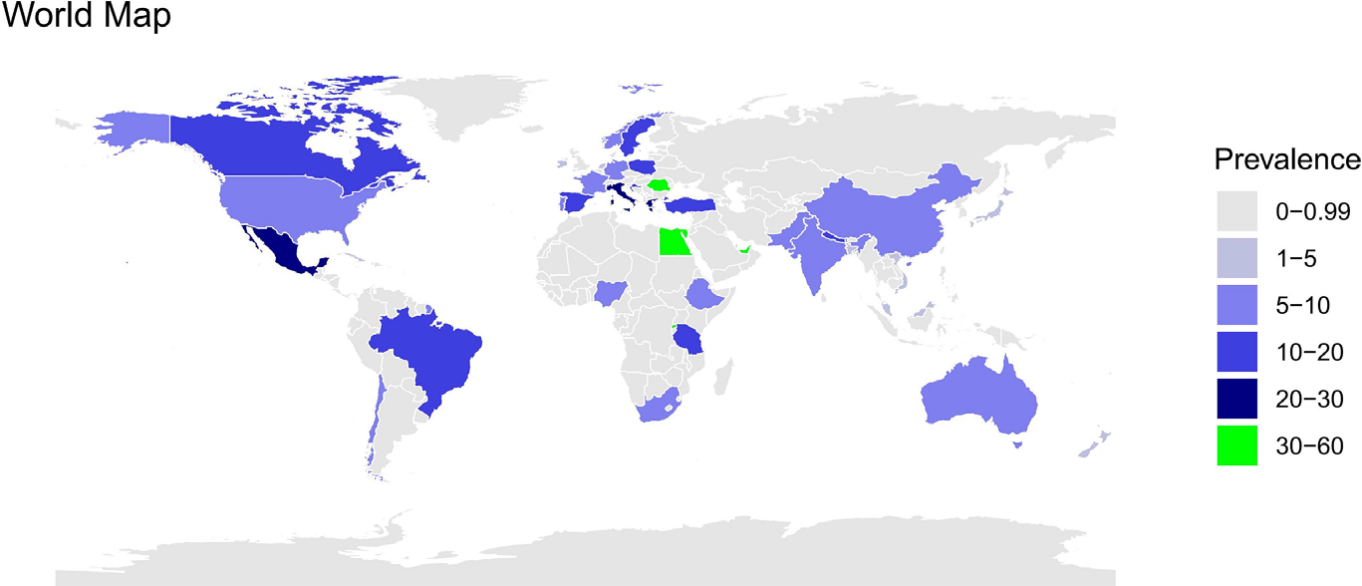
Distribution of the prevalence rates of co-morbid anxiety and depression in pregnancy and postpartum across countries in world map.

Analysis conducted by study regions showed that low- and middle-income countries yielded a higher rate of maternal CAD (low-income countries: 9%, 95%CI 7%–13%, *I*^2^ = 98.7%, *n* = 12; middle-income countries: 9%, 95%CI 8%–10%, *I*^2^ = 98.7%, *n* = 43), while high-income countries exhibited the lowest prevalence (8%, 95%CI 6%–9%, *I*^2^ = 99.7%, *n* = 67) (see in Supplementary Figure S1). Meanwhile, we also provided the worldwide map to visualize the prevalence rates in these 43 countries (see Figure 4).

Regarding the COVID-19 context, women in the COVID-19 pandemic (13%, 95%CI 9%–19%, *I*^2^ = 99.1%, *n* = 14) yielded a higher prevalence rate of CAD than those were not during the COVID-19 period (9%, 95%CI 7%–10%, *I*^2^ = 99.4%, *n* = 94), and the prevalence rates in the Post COVID-19 period were 6% (95%CI 2%–19%, *I*^2^ = 99.4%, *n* = 8) (Supplementary Figure S2). Publication year was classified to compare the prevalence of CAD from prior to and post 2016. The prevalence rates in studies published before and after 2016 were 8% (95%CI 7%–10%, *I*^2^ = 99.6%, *n* = 49) and 9% (95%CI 8%–11%, *I*^2^ = 99.1%, *n* = 73), respectively (Supplementary Figure S3).

### Test of heterogeneity

The heterogeneity in reported CAD prevalence among pregnancy and postpartum women bears statistical significance (*I*^2^ = 99.5%, *p* <0.0001). Similarly, significant heterogeneity was also observed in these subgroup analyses (see Table 1). Further, meta-regression was also performed, and it found that the prevalence rates significantly varied based on time points, study design, and assessment tools for anxiety and depression, study countries, while prevalence was not conditional on the publication year, country income level, and COVID-19 context (see Supplementary Table S4).

**Table 1.** Subgroup analysis of the prevalence of co-morbid anxiety and depression

Outcome variables	No. of Studies	ES (95%CI)	*I*^2^ (95%CI)	*p*-value
Pregnancy periods				
The first trimester	14	15% [11%, 21%]	98.2% [97.7%,98.5%]	<0.0001
The second trimester	14	9% [6%, 13%]	97.9% [97.3%,98.3%]	<0.0001
The third trimester	26	10% [7%, 13%]	98.9% [98.6%,99.0%]	<0.0001
Pregnancy (no specific trimester)	32	7% [5%, 11%]	99.7% [99.7%,99.7%]	<0.0001
**Perinatal (no specific time points)**	14	11% [6%, 21%]	99.9% [99.9%,99.9%]	<0.0001
**Postpartum periods**				
Within 1 week postpartum	4	13% [5%, 33%]	98.5% [97.3%,99.1%]	<0.0001
1 week to 1 month postpartum	6	8% [5%, 12%]	89.6% [82.5%,93.6%]	<0.0001
1 month to 2 month postpartum	13	10% [6%, 17%]	96.3% [94.5%,97.4%]	<0.0001
2 month to 4 month postpartum	13	6% [4%, 9%]	96.7% [95.4%,97.5%]	<0.0001
4 month to 6 month postpartum	12	7% [4%, 10%]	96.1% [94.5%,97.2%]	<0.0001
6 month to 12 month postpartum	11	4% [3%, 6%]	96.7% [95.5%,97.6%]	<0.0001
Postpartum (no specific time points)	20	15% [11%, 20%]	98.9% [98.7%,99.1%]	<0.0001
**Publication year**				
Before 2016	49	8% [7%, 10%]	99.6% [99.5%,99.7%]	<0.0001
2016–2024	73	9% [8%, 11%]	99.1% [98.9%,99.3%]	<0.0001
**Covid–19 context**				
Pre_COVID	94	9% [7%, 10%]	99.4% [99.3%,99.4%]	<0.0001
Dur_COVID	14	13% [9%, 19%]	99.1% [98.8%,99.3%]	<0.0001
Unclear	5	9% [7%, 11%]	96.9% [95.6%,97.8%]	<0.0001
Mixed_Context	2	12% [1%, 100%]	97.4% [94.6%,98.6%]	<0.0001
Post_COVID	8	6% [2%, 19%]	99.4% [99.1%,99.5%]	<0.0001
**Study design**				
Cross-sectional study	63	12% [10%, 16%]	99.8% [99.8%,99.8%]	<0.0001
Longitudinal study	23	9% [7%, 11%]	97.1% [96.5%,97.5%]	<0.0001
Cohort study	31	7% [5%, 8%]	98.9% [98.7%,99.1%]	<0.0001
Clinical trial study	3	4% [1%, 12%]	98.9% [98.2%,99.2%]	<0.0001
Case control study	2	7% [5%, 9%]	—	0.7831
CAD Disorders *				
Yes	26	6% [5%, 9%]	98.4% [98.0%, 98.7%]	<0.0001
Unclear	96	9% [8%, 11%]	99.6% [99.6%, 99.6%]	<0.0001
**Country income level**				
High-income countries or region	67	8% [6%,9%]	99.7%[99.6%,99.8%]	< 0.0001
Middle-income countries or region	43	9% [8%, 10%]	98.7%[98.7%,99.2%]	< 0.0001
Low-income countries or region	12	9% [7%, 13%]	98.7%[98.4%,98.9%]	<0.0001
**Study country**				
China	17	10% [7%, 13%]	97.8%[97.3%,98.1%]	< 0.0001
Australia	12	7% [5%, 10%]	96.5%[95.4%,97.3%]	< 0.0001
US	13	7% [5%, 10%]	98.6%[98.3%,98.8%]	< 0.0001
Nigeria	5	9% [5%, 17%]	99.1%[98.8%,99.3%]	< 0.0001
Pakistan	5	7% [3%, 15%]	99.2%[99.1%,99.4%]	< 0.0001
Canada	4	18% [11%, 29%]	97.3%[96.2%,98.0%]	< 0.0001
Ethiopia	4	10% [8%, 13%]	85.2%[79.1%,89.1%]	< 0.0001
India	4	8% [4%, 19%]	93.2%[88.6%,95.9%]	< 0.0001
Multi−country	3	16% [12%,21%]	95.4%[93.3%,96.9%]	< 0.0001
Brazil	3	15% [13%, 17%]	56.1%[21.8%,79.4%]	0.1028
Sweden	3	13% [7%, 24%]	88.8%[75.2%,95.1%]	< 0.0001
Turkey	3	12% [5%, 29%]	98.9%[98.6%,99.1%]	< 0.0001
Singapore	3	10% [6%, 18%]	92.8%[86.1%,96.2%]	< 0.0001
Portugal	3	9% [8%, 11%]	7.06%[0%,38.4%]	0.3711
France	3	9% [3%, 32%]	96.8%[93.0%, 98.6%]	< 0.0001
Greece	2	25% [16%, 38%]	94.4%[91.0%,96.5%]	< 0.0001
Egypt	2	52% [34%, 81%]	97.4%[96.3%,98.1%]	< 0.0001
Mexico	2	26% [8%, 86%]	97.6%[96.4%,98.2%]	< 0.0001
Italy	2	21% [2%, 100%]	99.7%[99.6%,99.8%]	< 0.0001
Spain	2	17% [6%, 51%]	97.1%[95.9%,97.8%]	< 0.0001
South Africa	2	8% [7%, 10%]	41.4%[0%,73.1%]	0.1816
Germany	2	7% [1%, 67%]	98.9%[98.3%,99.3%]	< 0.0001
Norway	2	6% [4%, 7%]	84.5%[67.8%,93.1%]	0.0112
Japan	2	5% [2%, 9%]	97.3%[96.3%,98.0%]	< 0.0001
Malaysia	2	4% [1%, 18%]	95.3%[90.7%,97.6%]	< 0.0001
The Netherlands	2	2% [1%, 3%]	87.6%[75.1%,94.3%]	0.0045
Vietnam	2	4% [3%, 6%]	0%	0.4328
UK	1	1% [1%, 3%]	—	—
Romania	1	50% [39%, 61%]	—	—
Rwanda	1	32% [25%, 39%]	—	—
United Arab Emirates	1	32% [28%, 37%]	—	—
Tanzania	1	18% [13%, 24%]	—	—
Croatia	1	15% [11%, 20%]	—	—
Poland	1	12% [10%, 14%]	—	—
Nepal	1	12% [10%, 15%]	—	—
Israel	1	11% [8%, 14%]	—	—
Ghana and Côte D’Ivoire	1	11% [8%, 13%]	—	—
Cyprus	1	10% [8%, 13%]	—	—
Chile	1	9% [6%, 13%]	—	—
Qatar	1	4% [3%, 5%]	—	—
Cuba	1	4% [3%, 6%]	—	—
Bangladesh	1	3% [2%, 5%]	—	—
Ireland	1	2% [2%, 2%]	—	—
New Zealand	1	1% [1%, 2%]	—	—
**Measurement tool for anxiety**				
STAI	21	11% [8%, 14%]	97.7%[97.4%,98.0%]	< 0.0001
GAD–7	18	13% [10%, 16%]	97.9%[97.6%,98.1%]	< 0.0001
HADS	16	11% [7%, 17%]	99.3%[99.2%,99.4%]	< 0.0001
DASS 21	8	4% [3%, 7%]	97.2%[96.5%,97.7%]	< 0.0001
SAS	7	9% [6%, 14%]	98.7%[98.4%,98.9%]	< 0.0001
STAI-S	5	12% [7%, 20%]	96.5%[94.7%,97.6%]	< 0.0001
SCID	4	5% [2%, 10%]	94.9%[91.1%,97.0%]	< 0.0001
Self-reported anxiety	4	4% [2%, 7%]	98.9%[98.6%,99.1%]	< 0.0001
BAI	3	18% [6%, 53%]	96.5%[92.7%,98.3%]	< 0.0001
CIDI	3	6% [1%, 45%]	89.3%[79.2%,94.6%]	< 0.0001
MINI	3	5% [2%, 13%]	96.2%[92.3%,98.2%]	< 0.0001
Recorded anxiety	3	4% [2%, 12%]	99.9%[99.9%,99.9%]	< 0.0001
HARS	2	26% [8%, 86%]	97.6%[96.4%,98.2%]	< 0.0001
PRIME-MD	2	15% [8%, 31%]	90.8%[81.2%,95.6%]	< 0.0001
STAI/EPDS–3ANXIETY	2	12% [4%, 37%]	98.3%[97.3%,99.0%]	< 0.0001
PRA	2	6% [5%, 8%]	6.9%[0%,29.7%]	0.3676
SRAS	2	7% [4%, 12%]	96.5%[94.7%,97.6%]	< 0.0001
PHQ–4	1	63% [59%, 66%]	—	—
STAI-T	1	28% [15%, 51%]	—	—
DASS 42	1	21% [18%, 25%]	—	—
BSI–18	1	18% [13%, 24%]	—	—
CIS-R	1	16% [13%, 18%]	—	—
GAD–2	1	16% [13%, 20%]	—	—
HSCL 25	1	12% [10%, 15%]	—	—
PASS	1	9% [6%, 13%]	—	—
AKUADS	1	8% [5%, 13%]	—	—
Previous diagnosis	1	8% [3%, 17%]	—	—
SCL–4a	1	6% [6%, 6%]	—	—
AUDADIS-IV	1	5% [4%, 6%]	—	—
SCL–25	1	5% [4%, 6%]	—	—
PHQ-ANXIETY	1	3% [2%, 5%]	—	—
Auto-CIDI	1	2% [2%, 3%]	—	—
BSI	1	1% [1%, 2%]	—	—
Computerized adaptive diagnostic tool CAT-MH®	1	1% [1%, 2%]	—	—
**Measurement tool for depression**				
EPDS	51	10% [8%, 11%]	97.8%[97.5%,98.1%]	< 0.0001
HADS	14	10% [6%, 17%]	99.3%[99.2%,99.4%]	< 0.0001
PHQ–9	11	10% [8%, 13%]	95.2%[93.7%,96.4%]	< 0.0001
DASS 21	6	5% [3%, 7%]	97.5%[96.8%,98.0%]	< 0.0001
CESD	5	14% [9%,22%]	99.0%[98.7%,99.2%]	< 0.0001
SDS	4	15% [10%, 22%]	98.4%[97.6%,98.9%]	< 0.0001
Self-reported depression	3	5% [3%, 9%]	98.3%[97.3%,98.8%]	< 0.0001
SCID	3	3% [2%, 5%]	51.6%[13.4%,77.0%]	0.1024
CIDI	2	4% [0%,100%]	92.3%[83.7%,96.5%]	0.0003
Recorded depression	2	3% [1%, 10%]	100.0%[100.0%,100.0%]	< 0.0001
PHQ–4	1	63% [59%, 66%]	—	—
HDRS	1	47% [42%, 53%]	—	—
PHQ–8	1	25% [24%, 26%]	—	—
PRIME-MD	1	22% [17%, 27%]	—	—
DASS 42	1	21% [18%, 25%]	—	—
CIS-R	1	16% [13%, 18%]	—	—
The Tale of Whooley’s two questions	1	16% [13%, 20%]	—	—
BDI	1	14% [9%, 20%]	—	—
MINI	1	13% [10%, 15%]	—	—
HSCL 25	1	12% [10%, 15%]	—	—
AKUADS	1	8% [5%, 13%]	—	—
Previous diagnosis	1	8% [3%, 17%]	—	—
EPDS PRIME-MD	1	7% [5%, 11%]	—	—
EPDS/PHQ–9	1	7% [5%, 9%]	—	—
SCL–4d	1	6% [6%, 6%]	—	—
AUDADIS-IV	1	5% [4%, 6%]	—	—
SRDS	1	5% [4%, 8%]	—	—
PRIME‑MD PHQ	1	3% [1%, 5%]	—	—
BSI	1	1% [1%, 2%]	—	—
Computerized adaptive diagnostic tool CAT-MH®	1	1% [1%, 2%]	—	—

*Note:* AKUADS, Aga Khan University Anxiety and Depression Scale; AUDADIS-IV, Disorder and Associated Disabilities Interview Schedule-DSM-IV; BAI, Beck Anxiety Inventory; BSI, Brief Symptom Index; CESD, Center for Epidemiological Studies-Depression scale; CIDI, Composite International Diagnostic Interview; CIS-R, Clinical Interview Schedule – Revised Version; DASS, Depression, Anxiety and Stress Scales; EPDS, Edinburgh Postnatal Depression Scale; GAD, Generalized Anxiety Disorder scale; HADS, Hospital Anxiety and Depression Scales; HARS, Hamilton Anxiety rating scale; HSCL, Hopkins Symptom Checklist; MINI, Mini International Neuropsychiatric Interview; PASS, Perinatal Anxiety Screening Scale; PHQ, Patient Health Questionnaire; PRA, Pregnancy-related anxiety scale; PRIME-MD, Primary Care Evaluation of Mental Disorders; SAS, Self-Rating Anxiety Scale; SCID, Structured Clinical Interview; SCL, Symptom Check List; SDS, Self-Rating Depression Scale; SRAS, Zung’s Self Rating Anxiety Scale; STAI, State–Trait Anxiety Inventory; STAI-S, Spielberger State Anxiety Inventory; STAI-T, Spielberger Trait Anxiety Inventory.

### Publication bias and sensitivity analysis

According to the visual inspection of the funnel plot (Supplementary Figure S4), no publication bias was shown in the included studies, which is consistent with the results of Egger’s test (*t* = −0.22, p = 0.82).

Sensitivity analysis was performed by removing studies one by one, which showed no study affected the prevalence estimate of CAD more than 0.004%, and the *I*^2^ index of heterogeneity test varied from 99.4% to 99.5%, suggesting that the results of the meta-analysis were reliable.

## Discussion

This systematic review and meta-analysis provided an up-to-date prevalence of CAD during the perinatal periods. The overall prevalence of CAD was reported to be 9%, with 9% in pregnancy and 8% in postpartum periods. Specifically, based on the different assessment time points, the pooled estimate of CAD was 15% in the first trimester, decreased to 9% in the second trimester, and then increased slightly to 10% in the third trimester. Meanwhile, during the postpartum period, the rate of CAD was highest at 13% in the first week after childbirth. It gradually decreased over the first year, ranging from 4% to 10%. The results of meta-regression analyses showed that the prevalence of CAD was influenced by assessment time points, study design, and the assessment tools used for anxiety and depression. However, it was not conditional on the publication year, country income level, and COVID-19 context.

In our meta-analysis, we found that the overall prevalence of CAD (9.0%, 8%–10%) was similar to the reporting results of the previous review with 9.5% (7.8%–11.2%) in antenatally and 8.2% (6.5%–9.9%) in postnatally (Falah-Hassani et al., 2017). Although our findings suggested that the prevalence was not conditional on the publication year before and after 2016, we observed that the prevalence in the first trimester and third trimester of pregnancy and within 1 month postpartum was slightly higher than the publication of the only meta-analysis of CAD in pregnancy and postpartum, while the prevalence in second trimester and from 2 to 12 months postpartum was lower than that of previous publication (Falah-Hassani et al., 2017). One possible reason is that previous reviews included 24 published and 42 unpublished original articles with some data of CAD collected through contacting the corresponding author (Falah-Hassani et al., 2017), while our up-to-date meta-analysis only included these studies that directly reported the prevalence of CAD, and there added additional 91 published studies to previous reviews. This dramatic increase in publications related to maternal CAD allowed us to perform a more refined estimate analysis. For example, the current meta-analysis added the number of studies at different stages in pregnancy and postpartum, and was able to examine the prevalence of CAD from delivery to 1 year postpartum thus allowing for analysis of periods of increased/decreased risks. Additionally, previous studies suggested that anxiety and depressive symptoms are more prevalent in early pregnancy and within 6 weeks postpartum (Bjørk, Veiby, Engelsen, & Gilhus, 2015; Langan Martin, McLean, Cantwell, & Smith, 2016; Viswasam, Eslick, & Starcevic, 2019). Moreover, a higher incidence rate was also observed at the early stage postpartum when controlled by previous psychological conditions (Langan et al., 2016; Nakić Radoš, Tadinac, & Herman, 2018). Notably, there are relatively limited studies reporting the prevalence of CAD within 1 week postpartum and from 1 week to 1 month postpartum, which indicates more research is needed in these two time points to determine an accurately estimated prevalence and strengthen the current results.

In our meta-analysis, the prevalence estimates were not dependent on country income level, this result was consistent with previous reviews indicating that country income level was not conditional on the prevalence of antenatal and postnatal CAD (Falah-Hassani et al., 2017). However, a comparison of the prevalence was observable between low-, middle- and high-income countries, findings suggested that a higher prevalence of CAD was observed in low- and middle-income countries, while high-income countries yielded the lowest prevalence rate. Furthermore, some studies synthesized the evidence of pure anxiety and depression in the perinatal period from high-income, low- and middle-income countries, indicating that there was a high prevalence of anxiety and depression occurring in low-income countries, with 24.0% (15.3%–33.8%) of anxiety and 20.7% (18.4%–23.0%) of depression in low-income countries, respectively (Roddy Mitchell et al.,2023a; Roddy Mitchell et al.,2023b). Notably, there was a small number of studies reporting the prevalence rate of CAD from low-income countries in the current meta-analysis, and most were from countries such as Pakistan, Nepal, Tanzania, and Rwanda. Besides, the assessment tools used are different in each low-income country, it is plausible that this high prevalence may be influenced by the assessment tool. Thus, the elevated prevalence may not accurately reflect a true statistic, and further studies on CAD in low-income countries are essential to validate this statistic in the future.

The impact of measurements was evaluated in the current meta-analysis, which showed that a statistical difference was also observed in these measurements used in included studies. The highest prevalence of anxiety and depression was at 63% for Patient Health Questionnaire 4 (PHQ-4) and the lowest prevalence was at 1% for Brief Symptom Index (BSI) and Computerized adaptive diagnostic tool CAT-MH®. One of the significant reasons for this finding may be due to the sensitivity of tools used to screen positive for anxiety and depression during pregnancy and the postpartum period, and the estimated prevalence by self-reported scales depended on the sensitivity and specificity of the scales endorsing anxiety and depression (Rondung, Massoudi, Nieminen, Wickberg, Peira, & Hultcrantz, 2024). Several reviews indicated that different cut-off values could affect the sensitivity or specificity of instruments, thereby influencing the estimated prevalence of maternal anxiety and depression from different cultural backgrounds (Ali, Ryan, & De Silva, 2016; Chorwe-Sungani, & Chipps, 2017; Fellmeth et al., 2021). For example, previous studies aimed to synthesize the optimal cut-offs for depression screening tools, which found the PHQ-9 was 8 (Zhou, Radojčić, Ashton-James, Yang, & Chen, 2023), while the EPDS was 11 (Levis, Negeri, Sun, Benedetti, & DEPRESSD EPDS Group, 2020). Besides, there is no evidence for which instruments are more suitable for maternal anxiety and depression (Rondung et al., 2024), the impact of assessment tools on results needs to be further demonstrated in the future. Furthermore, there were 32 measurements for anxiety and 29 measurements for depression in the current meta-analysis, and most of the tools were not recommended in guidelines to screen for maternal anxiety and depression, their usefulness for identification of maternal anxiety and depression during the perinatal period still remains uncertain. In addition, the context of measurement application may also contribute to this finding. A systematic review conducted by Smith et al., indicated that the context of screening tools is the critical factor determining the validity, and the application context of tools not only emphasizes geographical location but also refers to socio-economic background, language, educational level, maternal age, and cultural background of women in the target population (Sambrook Smith et al., 2022). This finding highlighted the importance of establishing the recommended tools for screening perinatal anxiety and depression in different cultures to better estimate the global prevalence of CAD.

We further explored the impact of COVID-19 context on the prevalence of CAD in pregnancy and postpartum, and no statistical difference was found. Significantly, the prevalence rates of perinatal CAD during COVID-19 period (13%, 9%–19%) were higher than of prevalence rates in non-COVID-19 background (9%, 7%–10%), but lower than the results of previous reviews with 18% during COVID-19 period (Sun, Zhu, Tao, Ma, & Jin, 2021). However, this review included only 14 studies conducted in the early stages of COVID-19. In addition, although the COVID-19 pandemic has increased the incidence of anxiety and depression, countries have taken proactive measures to address the impact of COVID-19 on maternal mental health (Kelly, Drogin, McSherry, & Donnelly, 2020), which may be one of the reasons for the lack of difference in the comorbidity of maternal anxiety and depression before and after COVID-19. Thus, this result needs to be further verified, but it highlights the need to pay special attention to maternal psychological problems during major public health emergencies.

## Strengths and limitations

The strength of this systematic review and meta-analysis provided an up-to-date prevalence rates of CAD during the pregnancy and postpartum periods. Moreover, this review explored the subgroup analyses based on different time points of assessment, assessment tools, study design, COVID-19 context, study country, and publication year on the prevalence of CAD. Furthermore, to address the issue of heterogeneity, meta-regression analyses were performed to test the sources of heterogeneity. Additionally, the consideration of the COVID-19 context was integrated into this review since the COVID-19 pandemic had a significant impact on global maternal mental health.

Our review also had some limitations. First, the majority of studies employed self-reported measures rather than using clinical diagnostic interviews for assessing CAD. In order to obtain more comprehensive incidence data, we did not distinguish between symptoms and disorders but looked at the population as a whole. Second, even though we employed a random-effect model, subgroup analysis, and meta-regression analysis to investigate the variability, there was still obvious heterogeneity in the current meta-analysis, which may lead to publication bias. Third, we only included articles published in the English language, which may result in selection bias for this review. Fourth, the CAD rates in the 1 week postpartum should be interpreted with caution due to the baby blues, and future studies should distinguish this issue and provide clearer reporting. Finally, maternal characteristics (e.g. age, parity, and marital status) have an effect on the prevalence of CAD, however, this meta-analysis failed to summarize these results due to the inconsistencies in the original research reports.

## Implication

The findings of this review have provided valuable insight into CAD during pregnancy and postpartum periods. First, the high prevalence rate of CAD in early pregnancy and early postpartum highlights the need for increased attention to women’s mental health during these critical periods in clinical practice. Second, in low- and middle-income nations where rates are higher than those observed in high-income countries. These lower-income regions often face resource constraints, for instance, they may lack adequate medical resources and have not established standardized mental health services. Therefore, prioritizing the mental health needs of women in low- and middle-income countries is essential (Nielsen-Scott, Fellmeth, Opondo, & Alderdice, 2022). It is important to mention that only 12 articles containing co-morbidity data from low-income countries were included in this meta-analysis, thus, further studies are necessary to enhance the reliability of these findings. Third, we identified a variety of tools used for measuring anxiety and depression, which contributes to heterogeneity in reported prevalence rates. The choice of measurement instruments can influence both sensitivity and accuracy when screening for anxiety and depression among affected populations. Consequently, future research should focus on identifying recommended measurement tools tailored for women with similar cultural backgrounds and characteristics across different nations to better standardize reporting practices regarding prevalence rates. Finally, the current original study was unclear about the reporting of assessment tools and maternal characteristics, as well as the timing of assessments, and it is suggested that follow-up studies could add clear and transparent reporting of these aspects.

## Conclusion

This systematic review and meta-analysis suggested that approximately one in 10 women experience CAD during pregnancy and postpartum. Meanwhile, the prevalence rates of CAD in the included 122 studies ranged from 1% to 73%. This variation may reflect differences in the study countries, assessment time points, and the assessment tools used for anxiety and depression. Future research should focus on this comorbidity and develop a targeted intervention approach and identify strategies for preventing and treating this problem, especially for women in the early pregnancy and early stage in postpartum.

## Supporting information

Ou et al. supplementary material 1 Ou et al. supplementary material

Ou et al. supplementary material 2 Ou et al. supplementary material

Ou et al. supplementary material 3 Ou et al. supplementary material

Ou et al. supplementary material 4 Ou et al. supplementary material

Ou et al. supplementary material 5 Ou et al. supplementary material

## Supplementary material

The supplementary material for this article can be found at http://doi.org/10.1017/S0033291725000601.
