# Supplementary material for: Prevalence of co-morbid anxiety and depression in pregnancy and postpartum: a systematic review and meta-analysis
Source: Psychol Med. 2025 Mar 13;55:e84. doi: 10.1017/S0033291725000601 (PMC12080659; doi:10.1017/S0033291725000601)
Supplement: Ou et al. supplementary material 1 — Ou et al. supplementary material [file S0033291725000601sup001.docx]

**Appendix**

**Title:** Prevalence of co-morbid anxiety and depression in pregnancy and postpartum: A systematic review and meta-analysis

**Authors:** Lu Ou^1^, Quan Shen ^2^, Meili Xiao ^1,*^, Weihong Wang^1,*^, Tan He ^2^, Binglu Wang ^2^

^1^ School of Nursing, Hunan Normal University , Changsha, Hunan Province, China.

^2^ Xiangya Nursing School of Central South University, Changsha, Hunan Province, China.

**^*^Address correspondence to:** School of Nursing, Hunan Normal University, 371 Tongzipo Road, Yuelu District, Changsha, Hunan 410013, China. E-mail: 202301064@hunnu.edu.cn

Lu Ou and Quan Shen contributed to the work equllly and should be regarded as co-first authors.

Meili Xiao and Weihong Wang contributed to the work as the corresponding author.

**Conflict of interest:** None

**Source of funding:** None

The review contains 4539 words.

Online Supplementary Table S1. Search strategies

| PubMed (n = 10,652) 【~2024/12/10】 |
| --- |
| #1 "Depression"[MeSH Terms] OR "Depressive Disorder"[MeSH Terms] OR "depression, postpartum"[MeSH Terms]  #2 "depression" [Title/Abstract] OR "depressive disorders"[Title/Abstract] OR "postpartum depression"[Title/Abstract] OR "depressive symptoms" [Title/Abstract]  #3 #1 OR #2  #4 ("Anxiety"[Mesh]) OR "Anxiety Disorders"[Mesh]  #5 "anxiety disorders"[Title/Abstract] OR "anxiety"[Title/Abstract]  #6 #4 OR #5  #7 #3 AND #6  #8 "Postpartum Period"[MeSH Terms] OR "Pregnancy"[MeSH Terms]  #9 "puerperium"[Title/Abstract] OR "gestation"[Title/Abstract] OR "postpartum period"[Title/Abstract] OR "pregnancy"[Title/Abstract] OR "pregnant"[Title/Abstract] OR "antenatal"[Title/Abstract] OR "prenatal"[Title/Abstract] OR "postnatal"[Title/Abstract] OR "postpartum"[Title/Abstract] OR "postbirth"[Title/Abstract] OR "post-birth"[Title/Abstract] OR "birth"[Title/Abstract] OR "childbirth"[Title/Abstract] OR "perinatal"[Title/Abstract] OR "perinatal period"[Title/Abstract]  #10: #8 OR #9  #11: #7 AND #10 |
| Embase (n = 19,416) 【~2024/12/10】 |
| #1 'depression'/exp OR 'perinatal depression'/exp OR 'antenatal depression'/exp OR 'postnatal depression'/exp  #2 'depression':ab,ti OR 'perinatal depression':ab,ti OR 'antenatal depression':ab,ti OR 'postnatal depression':ab,ti OR 'depressive symptoms':ab,ti OR 'depressive disorder':ab,ti OR 'postpartum depression':ab,ti  #3 #1 OR #2  #4 'anxiety'/exp OR 'anxiety disorder'/exp  #5 'anxiety':ab,ti OR 'anxiety disorder':ab,ti  #6 #4 OR #5  #7 #3 AND #6  #8 'puerperium'/exp OR 'pregnancy'/exp  #9 'puerperium':ab,ti OR 'gestation':ab,ti OR 'postpartum period':ab,ti OR 'pregnancy':ab,ti OR 'pregnant':ab,ti OR 'antenatal':ab,ti OR 'prenatal':ab,ti OR 'postnatal':ab,ti OR 'postpartum':ab,ti OR 'postbirth':ab,ti OR 'post-birth':ab,ti OR 'birth':ab,ti OR 'childbirth':ab,ti OR 'perinatal':ab,ti OR 'perinatal period':ab,ti  #10 #8 OR #9  #11 #7 AND #10 |
| The Cochrane Library (n = 2,897)【~2024/12/10】 |
| #1 MeSH descriptor: [Depression] explode all trees  #2 MeSH descriptor: [Depressive Disorder] explode all trees  #3 MeSH descriptor: [Depression, Postpartum] explode all trees  #4 (depression OR depressive disorders OR postpartum depression OR depressive symptoms):ti,ab,kw  #5 #1 OR #2 OR #3 OR #4  #6 MeSH descriptor: [Anxiety] explode all trees  #7 MeSH descriptor: [Anxiety Disorders] explode all trees  #8 ("anxiety disorders" OR "anxiety"):ti,ab,kw  #9 #6 OR #7 OR #8  #10 #5 AND #9  #11 MeSH descriptor: [Postpartum Period] explode all trees  #12 MeSH descriptor: [Pregnancy] explode all trees  #13 (puerperium OR gestation OR pregnant OR antenatal OR prenatal OR postnatal OR postpartum OR postbirth OR "post-birth" OR birth OR childbirth OR perinatal OR "perinatal period" OR "postpartum period" OR pregnancy):ti,ab,kw  #14 #11 OR #12 OR #13  #15 #10 AND #14 |
| CINAHL (n = 2,831) 【~2024/12/31】 |
| #1 (MM "Depression+") OR (MM "Depression, Postpartum")  #2 SU depression OR depressive disorders OR postpartum depression OR depressive symptoms  #3 #1 OR #2  #4 (MM "Anxiety+") OR (MM "Anxiety Disorders+")  #5 SU anxiety disorders OR anxiety  #6 #4 OR #5  #7 #3 AND #6  #8 (MM "Postnatal Period+") OR (MM "Perinatal Period") OR (MM "Pregnancy+")  #9 SU puerperium OR gestation OR pregnant OR antenatal OR prenatal OR postnatal OR postpartum OR postbirth OR "post-birth" OR birth OR childbirth OR perinatal OR “perinatal period” OR "postpartum period" OR pregnancy  #10 #8 OR #9  #11 #7 AND #10 |
| PsycINFO (n = 5,848) |
| #1 mainsubject ("Depression" OR "Depressive Disorder" OR "depression, postpartum")  #2 tiab ('depression' OR 'depressive disorders' OR 'postpartum depression' OR 'depressive symptoms')  #3 #1 OR #2  #4 mainsubject ('Anxiety' OR 'Anxiety Disorder')  #5 tiab ('Anxiety' OR 'Anxiety Disorder')  #6 #4 OR #5  #7 #3 AND #6  #8 mainsubject ("Postpartum Period" OR "Pregnancy")  #9 tiab ("puerperium" OR "gestation" OR "postpartum period" OR "pregnancy" OR "pregnant" OR "antenatal" OR "prenatal" OR "postnatal" OR "postpartum" OR "postbirth" OR "post-birth" OR "birth" OR "childbirth" OR "perinatal" OR "perinatal period")  #10 #8 OR #9  #11 #7 AND #10 |
| Web of Science (n = 20,426) |
| #1 TS = (Depression OR “Depressive Disorder” OR “depression, postpartum”) OR TS=(depressive disorders OR postpartum depression OR depressive symptoms))  #2 TS = (Anxiety OR “Anxiety Disorders”))  #3 (TS = ("postpartum period" OR pregnancy OR puerperium OR gestation OR pregnant OR antenatal OR prenatal OR postnatal OR postpartum OR postbirth OR "post-birth" OR birth OR childbirth OR perinatal OR “perinatal period”))  #4 #1 AND #2 AND #3 |
| Scopus (n = 3,213) |
| #1 (TITLE-ABS-KEY (depression OR "Depressive Disorder" OR "depressive symptoms" OR "postpartum depression")  #2 TITLE-ABS-KEY (anxiety OR "Anxiety Disorders")  #3 TITLE-ABS-KEY ("perinatal period" OR "Postpartum Period" perinatal OR postpartum OR postnatal OR postbirth OR post-birth OR birth OR childbirth OR pregnancy OR pregnant OR antenatal OR gestation OR prenatal OR puerperium)  #4 #1 AND #2 AND #3 |
| Google Scholar (n= 10) |
| #1 allintitle: Comorbid Anxiety and Depression in postpartum  #2 allintitle: Comorbid Anxiety and Depression in pregnant  #3 allintitle: Comorbid Anxiety and Depression in perinatal  #4 #1 OR #2 OR #3 |

Online Supplementary Table S2. Characteristics of the included studies on the prevalence of antenatal comorbid depression and anxiety

| Author,  Year | Country | Study design | Setting | Data collection time periods | Sample size | Age | Participant's characteristics | Measurement tool | | Time points | Outcome of interest | |
| --- | --- | --- | --- | --- | --- | --- | --- | --- | --- | --- | --- | --- |
|  |  |  |  |  |  |  |  | For anxiety  (with cut-off value) | For depression  (with cut-off value) |  | n | N |
| Sutter-Dallay 2004^1^ | France | Prospective cohort study | Hospital | NR | 497 | 29.6 ±4.2 | Postpartum | MINI | EPDS;  12 | 6 weeks postpartum | 13 | 497 |
| Adewuya 2005^2^ | Nigeria | Longitudinal study | Home | April 2003 to July 2003 | 632 | 27.45±11.07 | Postpartum | SRAS;  NR-cut off value | SRDS;  NR-cut off value | 1 week postpartum | 62 | 632 |
|  |  |  |  |  | 630 |  |  |  |  | 4 weeks postpartum | 33 | 630 |
|  |  |  |  |  | 600 |  |  |  |  | 8 weeks postpartum | 43 | 600 |
|  |  |  |  |  | 547 |  |  |  |  | 12 weeks postpartum | 29 | 547 |
|  |  |  |  |  | 512 |  |  |  |  | 24 weeks postpartum | 21 | 512 |
|  |  |  |  |  | 480 |  |  |  |  | 36 weeks postpartum | 12 | 480 |
| Andersson 2006^3^ | Sweden | Cross-sectional study | Hospital | October 2, 2000 to October 1, 2001 | 650 | NA | Healthy women | PRIME-MD system;  NA-cut off value | PRIME-MD system;  NA-cut off value | Second trimester  (16-18 weeks) | 39 | 190 |
|  |  |  |  |  |  |  |  |  |  | 3 to 6 months postpartum | 25 | 107 |
| Miller 2006^4^ | Australia | Cross-sectional study | Hospital | NR | 325 | 32±4.6  18 ~ 44 | Primiparous mothers | DASS-21;  NA-cut off value | DASS-21;  NA-cut off value | 6 weeks to 6 months postpartum | 23 | 325 |
| Lee 2007^5^ | China | Longitudinal study | Hospital | NR | 335 | 31±4.8 | Pregnancy | HADS;  7 | HADS;  8 | First trimester  (12.5±1.2) | 48 | 335 |
|  |  |  |  |  |  |  |  |  |  | Second trimester (19.5±1.8) | 42 | 335 |
|  |  |  |  |  |  |  |  |  |  | Third trimester  (34.4±1.7) | 57 | 335 |
| Phillips 2007^6^ | Australia | Cross-sectional | Community | September 2005 to April 2006 | 167 | 31.4±5.1  17 ~ 44 | Postpartum women with unsettled infants | DSM-IV criteria | EPDS;  Major depression(13)  Minor depression(10-12) | 2 weeks to 12 months postpartum | 31 | 167 |
| Sato 2008^7^ | Japan | Longitudinal study | Hospital | January to December 2004 | 1348 | 32 median  17~45 | Postpartum | HADS  Possible level (8) | HADS  Possible level (8) | 3 to 4 months postpartum | 55 | 1348 |
|  |  |  |  |  |  |  |  |  |  | 9 to 10 months postpartum | 72 | 1348 |
|  |  |  |  |  |  |  |  | HADS  Definite level (11) | HADS  Definite level (11) | 3 to 4 months postpartum | 26 | 1348 |
|  |  |  |  |  |  |  |  |  |  | 9 to 10 months postpartum | 39 | 1348 |
| Rowe 2008^8^ | Australia | Cross-sectional study | Community | December 2005 to March 2006 | 138 | NR | Postpartum | CIDI-Auto | CIDI-Auto | 29±10.3 weeks postpartum | 15 | 138 |
| Reck 2008^9^ | Germany | Longitudinal study | Community | December 2003 to February 2005 | 1024 | 33±5  15 ~ 45 | Postpartum | SCID-I | SCID-I | First 3 months postpartum. | 21 | 1024 |
| Faisal-Cury 2009^10^ | Brazil | Cross-sectional study | Hospital | May to September 2005 | 831 | 25  16 ~ 44 | Pregnancy | CIS-R;  12 | CIS-R;  12 | 20 to 30 weeks pregnancy | 129 | 831 |
| Ali et 2009^11^ | Pakistan | Quasi-experimental | Community | February 2004 to December 2006 | 420 | 26.46 ±4.82 | Postpartum | AKUADS;  19;  DSMIV criteria | AKUADS;  19;  DSMIV criteria | 1 month postpartum | 14 | 267 |
|  |  |  |  |  |  |  |  |  |  | 2 months postpartum | 14 | 267 |
|  |  |  |  |  |  |  |  |  |  | 6 months postpartum | 27 | 267 |
|  |  |  |  |  |  |  |  |  |  | 12 months postpartum | 35 | 267 |
| GIAKOUMAKI 2009^12^ | Greece | Longitudinal study | Hospital | NR | 235 | 31.3 (4+ 6) [mean + s.e] | Postpartum | STAI-S;  40 | EPDS;  Major depression: 14 | 2-3 days postpartum | 86 | 235 |
|  |  |  |  |  | 175 | 31.5 (4+ 3) [mean + s.e] |  |  |  | 3 months postpartum | 48 | 175 |
|  |  |  |  |  | 235 | 31.3 (4+ 6) [mean + s.e] |  | STAI-T;  40 |  | 2 to 3 days postpartum | 88 | 235 |
|  |  |  |  |  | 175 | 31.5 (4+ 3) [mean + s.e] |  |  |  | 3 months postpartum | 35 | 175 |
| QIAO 2009^13^ | China | Cross-sectional study | Hospital | 6 May to 20 July 2007 | 527 | Normal group:  29.2 ± 4.1  Anxiety or depression group:  29.0 ± 4.3  18 ~ 45 | Pregnancy | HADS;  9 | HADS;  9 | 20 to 41 pregnancy weeks | 18 | 527 |
| Teixeira 2009^14^ | Portugal | Longitudinal study | Hospital | January 2006 to December 2007 | 270 | 28.94±6.49  20 ~ 39 | Pregnancy | STAI-S;  45 | EPDS;  10 | First trimester  (8-14 weeks) | 29 | 270 |
|  |  |  |  |  |  |  |  |  |  | Second trimester  (20-24 weeks) | 21 | 270 |
|  |  |  |  |  |  |  |  |  |  | Third trimester  (30-34 weeks) | 30 | 270 |
| Batenburg-Eddes2009^15^ | Netherlands | Longitudinal study | NR | April 2002 to January 2006 | 5296 | 31±4.9 | NA | BSI;  0.5 | BSI;  0.5 | Perinatal | 75 | 5296 |
|  |  |  |  |  | 4731 |  | Pregnancy |  |  | 20 weeks pregnancy | 46 | 4731 |
|  |  |  |  |  | 4731 |  | Pregnancy |  |  | 2 months postpartum | 49 | 3947 |
| Woolhouse 2009^16^ | Australia | Longitudinal study | Hospital | April 1, 2003 to December 31, 2005 | 1507 | ＞18 | Postpartum | Self-reported anxiety | Self-reported depression | 9 months postpartum | 45 | 1385 |
| Austin 2010^17^ | Australia | Retrospective cohort study | Hospital | September 1999 to March 2002 | 1289 | 31.3±4.43 | Postpartum | Auto-CIDI;  NR-cut off value | EPDS;  NR-cut off value | 6 to 8 months postpartum | 29 | 1289 |
| Goodman 2010^18^ | USA | Longitudinal study | Hospital | June 2006 to February 2007 | 491 | 31.6 ±5.35  18 ~ 45 | Pregnancy | PHQ-ANXIETY;  NR-cut off value | EPDS;  10 | Third trimester  (>28 weeks) | 17 | 491 |
|  |  |  |  |  |  |  | Postpartum |  |  | 6 weeks postpartum | 7 | 299 |
| Yelland 2010^19^ | Australia | Cross-sectional study | Hospital | September to  October 2007 | 4269 | 31(Mean)  16 ~ 46 | Postpartum | DASS 21;  8 | DASS 21;  10 | 6 months postpartum | 344 | 4269 |
| Fisher 2010^20^ | VietNam | Cross-sectional study | Hospital | November 2006 to March 2007 | 199 | 25.6 ± 5.0 | Pregnancy | DSM–IV (SCID) | DSM–IV (SCID) | Pregnancy | 8 | 199 |
| Field 2010^21^ | USA | Cohort study | Hospital | NR | 911 | M = 27.5  18 ~ 40 | Pregnancy | STAI;  48 | CES-D;  16 | Prenatal early GA  (20 weeks) | 308 | 911 |
| Edhborg 2011^22^ | Bangladesh | Cohort study | Community | July 2007 to August 2008 | 674 | 24.6±6.1 | Postpartum | STAI-S;  45 | EPDS;  10 | 2 to 3 months postpartum | 23 | 674 |
| Figueiredo 2011^23^ | Portugal | Longtitudal study | Hospital | NR | 260 | NR | Pregnancy | STAI-S;  45 | EPDS;  10 | First trimester | 24 | 260 |
| Le Strat 2011^24^ | United States | Cross-sectional study | Database | 2001 to 2002 | 1524 | ≥18 | Pregnant and postpartum | AUDADIS-IV | AUDADIS-IV | Perinatal | 78 | 1524 |
| Paul 2012^25^ | US | Secondary analysis of RCT | Hospital | September 12, 2006 to August 1, 2009 | 1123 | 29.0±5.5 | Postpartum ; women with "well" newborns ≥34 gestational weeks | STAI;  40 | EPDS;  12 | 3 to 5 days postpartum | 42 | 1123 |
| Tavares 2012^26^ | Brazil | Cross-sectional study | Home | July 2007 to March 2008 | 919 | 13 ~ 45 | Postpartum | MINI | MINI | within 30 to 90 days postpartum | 115 | 919 |
| Grant 2012^27^ | Australia | Longtitudal study | Hospital | NR | 88 | 31.82 ± 4.28 | Pregnancy | STAI;  40 | EPDS;  13 | Third trimester | 7 | 88 |
|  |  |  |  |  |  |  | Postpartum |  | EPDS;  10 | 7 months postpartum | 11 | 88 |
| Ali 2012^28^ | Pakistan | Cross-sectional study | Hospital | September 2005 to January 2006 | 165 | 27.92±4.7 | Pregnancy | HADS;  8 | HADS;  8 | Pregnancy | 55 | 167 |
| STORKSEN 2012^29^ | Norway | Cross-sectional study | Hospital | November 2008 to April 2010 | 1642 | 31±4.7  18 ~ 45 | Pregnancy | SCL-25;  18 | EPDS;  12 | 32 weeks pregnancy | 78 | 1642 |
| Bener 2012^30^ | Qatar | Cross-sectional study | Hospital | January 2010 to May 2011 | 1659 | ≤45 | Postpartum | DASS 21;  8 | DASS 21;  10 | Within 6 months postpartum | 63 | 1659 |
| Ban 2012^31^ | UK | Cross-sectional study | Database | 1994–2009 | 116457 | 31(Median)  15 ~ 45 | Pregnancy | Recorded anxiety | Recorded depression | Pregnancy | 1083 | 116 457 |
|  |  |  |  |  |  |  | Postpartum |  |  | 9 months postpartum | 2539 | 116 457 |
| Ibanez 2012^32^ | France | Cohort study | Hospital | September 2003 to January 2006 | 1719 | NR | Pregnancy | STIA;  37 | CES-D;  16 | 24 to 28 weeks pregnancy | 227 | 1719 |
| Bindt 2013^33^ | Ghana ;Côte  D'Ivoire | Prospective cohort  study | Hospital | March 2010 to December 2011 | 717 | 29.1 ± 5.5 | Low-Obstetric Risk Women | GAD-7;  10 | PHQ-9;  10 | Third trimester | 76 | 717 |
| Wynter 2013^34^ | Australia | Prospective study | Community | 2006 to 2007 | 172 | 30.6 ±5.0 | Postpartum | CIDI | CIDI | 6 months postpartum | 1 | 172 |
| Makara-Studzińska 2013^35^ | Poland | Longitudinal study | Hospital | January 2011 to May 2012 | 314 | 28.64±4.77  18 ~ 45 | Pregnancy | HADS;  8 | HADS;  8 | First trimester  (11.22±1.8) | 40 | 314 |
|  |  |  |  |  |  |  |  |  |  | Second trimester  (21.05 ± 2.9) | 34 | 314 |
|  |  |  |  |  |  |  |  |  |  | Third trimester  (32.99 ± 2.8) | 39 | 314 |
| Thiagayson 2013^36^ | Singapore | Cross-sectional study | Hospital | September 2011 to March 2012 | 200 | 31.0±4.8  18 ~ 41 | Inpatient high-risk pregnant women at 23 weeks or more of gestation | STAI;  42/43 | EPDS;  8/9 | 23 or more weeks pregnancy | 10 | 200 |
| Bjørk 2014^37^ | Norway | Prospective cohort study | Hospital | 1999–2008 | 87468 | NA | Peripartum | SCL-4a | SCL-4d | Second trimester to 6 months postpartum | 5510 | 87468 |
| Enatescu 2014^38^ | Romania | Cross-sectional study | Hospital | January to June 2013 | 80 | 28.9±4.8 | NR | BAI;  NA | EPDS;  10 | 6 to 8 weeks postpartum | 40 | 80 |
| Farr 2014^39^ | US | Cross-sectional study | Phone | 2009 to 2010 | 4451 | NR | Postpartum | Self-reported anxiety | Self-reported depression | 2 to 4 months postpartum | 327 | 4451 |
| Miller 2014^40^ | USA | Prospective cohort study | Hospital | June to September 2009 | 461 | 32.6±4.9 | Postpartum | STAI;  40 | PHQ-9;  10 | 2 weeks postpartum | 28 | 461 |
| Koutra 2014^41^ | Greece | Prospective cohort study | Hospital | Starting in February 2007 | 438 | 29.77 ±4.68  ＞16 | Pregnancy | STAI;  48 | EPDS;  13 | 28 to 32 weeks pregnancy (completed weeks:38.31±1.55) | 50 | 438 |
| Segre 2014^42^ | American | Cross-sectional study | Hospital | December 2010 to May 2012 | 200 | 28 ±5.7  18 ~ 45 | Mothers of Newborns Hospitalized on the Neonatal ICU | BAI;  8 | EPDS;  12 | Postpartum | 27 | 200 |
| Simpson 2014^43^ | Canada | Cross-sectional study | Hospital | January 2011 and February 2013 | 240 | 30.5±5.7  16 ~ 46 | NR | GAD-7;  13 | EPDS;  17 | NR | 100 | 240 |
| Tendais 2014^44^ | Portugal | Case control study | Hospital | NR | 148 | NR | Women with low medical risk pregnancies | STAI;  45 | EPDS;  9 | Pregnancy | 9 | 139 |
|  |  |  |  | NR | 99 |  |  |  |  | Postpartum | 5 | 94 |
| Nguyen 2015^45^ | Vietnam | Prospective cohort study | Hospital | January to February 2008 | 211 | 25.6 ±5.1  20 ~ 44 | NA | DSM-IV (SCID-I) | DSM-IV (SCID-I) | Perinatal | 10 | 211 |
|  |  |  |  |  |  |  | Postpartum |  |  | 1 year postpartum | 5 | 211 |
| Cheng 2015^46^ | Singapore | Cross-sectional study | Hospital | NR | 1062 | NA | Pregnancy | STAI;  ≥41(high anxiety state）  ≥43(high anxiety trait） | EPDS;  15 | 26 weeks pregnancy | 82 | 1062 |
| Martini 2015^47^ | Germany | Longitudinal study | Community | NR | 306 | NR | Pregnancy | CIDI; | CIDI; | 10 to 12 weeks pregnancy | 65 | 302 |
| Abdelhai 2015^48^ | Egypt | Cross-sectional study | Hospital | 20 January 2013 to 24 March 2013 | 376 | 27.80 ± 5.99 | Pregnancy | HADS;  10 | HADS;  10 | First to Third trimester | 237 | 376 |
|  |  |  |  |  |  |  |  |  |  | First trimester | 49 | 67 |
|  |  |  |  |  |  |  |  |  |  | Second trimester | 51 | 77 |
|  |  |  |  |  |  |  |  |  |  | Third trimester | 137 | 232 |
| Waldie 2015^49^ | New Zealand | Retrospective cohort study | NR | 25th April 2009 to 25th March 2010 | 5664 | NR | Pregnancy | Self-reported anxiety | EPDS;  13 | Third trimester | 78 | 5659 |
| Falah-Hassani 2016^50^ | Canada | Longitudinal study | Hospital | April 2001 to January 2002 | 667 | 28.7±5.1 | Postpartum | STAI-State;  40 | EPDS;  10 | 1 to 8 weeks postpartum | 87 | 667 |
|  |  |  |  |  |  |  |  |  |  | 1 weeks postpartum | 83 | 522 |
|  |  |  |  |  |  |  |  |  |  | 4 weeks postpartum | 59 | 478 |
|  |  |  |  |  |  |  |  |  |  | 8 weeks postpartum | 54 | 501 |
| Tham 2016^51^ | Singapore | Prospective cohort study | Hospital | NR | 313 | 30.3±5.02  18 ~ 41 | Peripartum | STAI-S;  40 | EPDS;  10 | 26 to 28 weeks pregnancy | 60 | 313 |
|  |  |  |  |  |  |  |  |  |  | 3 months post-pregnancy | 41 | 313 |
| Venkatesh 2016^52^ | American | Prospective cohort study | Hospital | July 2010 to June 2014 | 576 | NA | Pregnancy | Recorded anxiety; | EPDS;  12 | Third trimester  (24-28 weeks ) | 102 | 576 |
|  |  |  |  |  |  |  | Postpartum |  |  | 6 weeks postpartum | 24 | 576 |
| Chen 2016^53^ | China | Longitudinal study | Hospital | 2014 | 195 | (31.7±3.8）  24 ~ 41 | High-risk pregnant women | HADS;  9 | HADS;  9 | 16 to 20 weeks pregnancy | 5 | 195 |
|  |  |  |  |  |  |  |  |  |  | 6 months pregnancy | 5 | 190 |
|  |  |  |  |  |  |  |  |  |  | 7 months pregnancy | 6 | 186 |
|  |  |  |  |  |  |  |  |  |  | 8 months pregnancy | 7 | 179 |
|  |  |  |  |  |  |  |  |  |  | 9 months pregnancy | 3 | 153 |
| Dikmen-Yildiz 2017^54^ | Turkey | Longitudinal study | Hospital | May 2014 to  May 2015 | 950 | 27.6±5.3  18 ~ 44 | NA | HADS;  11【Unclear in anxiety subscale, only total】 | EPDS;  14(pregnancy)  13(postpartum) | 26 to 35 weeks pregnancy | 104 | 950 |
|  |  |  |  |  | 858 |  |  |  |  | 4 to 6 weeks postpartum | 161 | 858 |
|  |  |  |  |  | 829 |  |  |  |  | 6 months postpartum | 74 | 829 |
| Nasreen 2018^55^ | Malaysia | Cross-sectional | Hospital | March 2016 to August 2017 | 450 | 29.3±5.0 | Expectant mothers during pregnancy | DASS 21;  8 | EPDS;  12 | Third trimester | 35 | 450 |
|  |  |  |  |  | 454 | 28.9±4.1 |  |  |  | Third trimester | 37 | 454 |
| Odinka 2018^56^ | Nigeria | Cross-sectional | Hospital | November and December 2015 | 309 | 29.65±4.87  20 ~ 46 | Postpartum | HADS;  Mild –Severe: 8  Moderate –Severe: 11  Severe: 15 | HADS;  Mild –Severe: 8  Moderate –Severe: 11  Severe: 15 | 6 to 14 weeks postpartum | 205 | 309 |
| Aryal 2018^57^ | Nepal | Cross-sectional | Community | NR | 164 | 16 ~ 45 | Pregnancy | HSCL 25;  17.5 | HSCL 25;  24.5 | Pregnancy | 24 | 164 |
|  |  |  |  |  | 567 |  | Postpartum |  |  | Postpartum | 65 | 567 |
| Dennis 2018^58^ | Canada | Cohort study | Community | January 2011 to March 2014 | 571 | 31.6±4.4 | Chinese recent immigrant, nonrecent immigrant, and Canadian-born women | STAI;  40 | EPDS;  9 | 4 weeks postpartum | 81 | 564 |
|  |  |  |  |  |  |  |  |  |  | 12 weeks postpartum | 49 | 483 |
|  |  |  |  |  |  |  |  |  |  | 52 weeks postpartum | 49 | 454 |
| Dennis 2018^59^ | Canada | Cohort study | Community | NR | 571 | NA | Chinese recent immigrant, nonrecent immigrant, and Canadian-born women | STAI;  40 | EPDS;  9 | 24 weeks postpartum | 120 | 549 |
| Redinger 2018^60^ | South Africa | Prospective cohort study | Hospital | June 2014 to July 2016 | 946 | 29.7 (mean)  18~44 | Pregnancy | STAI;  12 | EPDS;  13 | First trimester  (⩽14 weeks) | 68 | 936 |
| Suzuki 2018^61^ | Japan | Cross-sectional study | Hospital | April 2016 to October 2016 | 470 | NR | Pregnancy | GAD-2；  NR-cut off value | The Tale of Whooley’s two questions | First trimester | 75 | 470 |
| Nakić Radoš 2018^62^ | Croatia | Cross-sectional study | Community | NR | 272 | 29.5±4.4 | Postpartum. | STAI;  40 | EPDS;  8 | 6 weeks postpartum. | 42 | 272 |
| González-  Hernández 2019^63^ | Mexico | Cross sectional  study | Hospital | May 2016 to November 2017 | 188 | 24.7 ± 6.4  15 ~ 42 | Mother of premature Babies in NICU | HARS;  18 | BDI;  17 | 2 weeks postpartum | 26 | 188 |
| Agbaje 2019^64^ | Nigeria | Cross-sectional study | Hospital | June to September 2017 | 267 | 28.9±6.2  ＞15 | Postpartum | HADS;  8 | EPDS;  13 | 4 weeks postpartum;  8 weeks postpartum;  12 weeks postpartum | 69 | 267 |
| Gelman 2019^65^ | Mexico | Cross-sectional study | Hospital | October 2014 to December 2016 | 298 | NR | Pregnancy | HARS;  NR-cut off value | HDRS;  NR-cut off value | Third trimester  (28–40 weeks) | 141 | 298 |
| Aris-Meijer 2019^66^ | The Netherlands | Prospective cohort study | Hospital | May 2010 to March 2015 | 3842 | Mean 30  18 ~ 45 | Pregnancy | STAI;  42 | EPDS;  10 | 12 weeks pregnancy | 85 | 3842 |
| Samwel Ngocho 2019^67^ | Tanzania | Cross-sectional study | Hospital | July 2016 to August 2017 | 200 | 30(median)  25-35(IQR) | Pregnancy Women living with HIV | BSI-18;  1.01 | EPDS;  10 | Second or Third trimester | 36 | 200 |
| Tay 2019^68^ | Australia | Cross-sectional study | Database | 2006;2009;2012;2010 | 5239 | 39.7±1.5 | Pregnancy | Self-reported anxiety | Self-reported depression | Pregnancy | 151 | 5239 |
|  |  |  |  |  |  |  | Postpartum |  |  | Postpartum | 455 | 5239 |
| Uguz 2019^69^ | Turkey | Cross-sectional study | Hospital | NR | 1119 | 28.31 ± 5.65  ≥18 | Pregnancy | Unclear | Unclear | NR | 36 | 1119 |
| Genovez 2019^70^ | American | Cross-sectional study | Community | April 2016 to June 2016 | 100 | 28.11±5.55 | Immigrants  from Central American countries | GAD-7;  10 | PHQ-9;  10 | 12 months postpartum | 5 | 100 |
| Ramakrishna 2019^71^ | Australia | Cross-sectional study | Community | NR | 1070 | 29.7 (mean)  18 ~ 45 | Postpartum | STAI ;  45 | EPDS;  13 | Postpartum  (0-12 months) | 143 | 1070 |
| Wassif 2019^72^ | Egypt | Cross-sectional study | Hospital | September 2016 to the end of January 2017 | 500 | 25.6±5.07  18 ~ 40 | Postpartum | DASS-42;  8 | DASS-42;  10 | Postpartum  ( 2 to 6 months ) | 106 | 500 |
| Redinger 2020^73^ | South Africa | Prospective  cohort study | Hospital | 2014–2016 | 649 | 29.6±5.9  29 (median)  IQR 25-34 | Pregnancy | STAI;  12 | EPDS;  13 | 14 to 18 weeks pregnancy | 49 | 649 |
|  |  |  |  |  |  |  |  |  |  | 24 to 28 weeks pregnancy | 63 | 649 |
| Umuziga 2020^74^ | Rwanda | Cross-sectional study | Hospital; | October 2013 | 165 | ＞15 | NA | SRAS;  45 | EPDS;  10 | Perinatal | 52 | 165 |
| González-Mesa 2020^75^ | Spain and Turkey | Cross-sectional study | Hospital | NR | 250 | 30.3 | Pregnancy | STAI;  30 | EPDS;  NR-cut off value | NR | 113 | 250 |
|  |  |  |  |  | 264 | 31.7 |  |  |  |  | 25 | 263 |
| Premji 2020^76^ | Pakistan | Prospective cohort | Hospital | NR | 300 | 26.8  18 ~ 43 | Pregnancy | PRA;  answered “very true” to 3 or more of the 10 items | EDS;  9 | 12 to 19 weeks pregnancy | 24 | 282 |
|  |  |  |  |  |  |  |  |  |  | 12 to 19 weeks pregnancy | 14 | 282 |
|  |  |  |  |  |  |  |  |  |  | 22 to 29weeks pregnancy | 16 | 282 |
|  |  |  |  |  |  |  |  |  |  | 22 to 29 weeks pregnancy | 14 | 282 |
| Kantipudi 2020^77^ | India | Cross-sectional study | Hospital | January 2019 to March 2019 | 209 | 26.00±4.30  18 ~ 38 | Pregnancy | GAD-7;  9 | PHQ-9;  10 | 1 to 9 months pregnancy | 28 | 209 |
| Pabon 2020^78^ | Brazil | Cross-sectional study | Hospital | NR | 533 | 28.9±6.7 | Pregnancy | GAD-7;  10 | PHQ-9;  10 | ≥28 weeks pregnancy | 85 | 531 |
| Coo 2021^79^ | Chile | Longitudinal study | Hospital | May 2018 to December 2019 | 148 | 28.6  19 ~ 43 | Expectant mothers during pregnancy | PASS;  42 | EPDS;  Pregnancy:13 | Third trimester  (38.99±1.3 weeks) | 9 | 148 |
|  |  |  |  |  |  |  | Expectant mothers at postpartum |  | EPDS;  Postnatal:10 | 3 months postpartum | 13 | 148 |
|  |  |  |  |  |  |  |  |  |  | 6 months postpartum | 17 | 148 |
| Dong 2021^80^ | China | Cross-sectional study | Online | February 22 to February 27 2020 | 156 | 20 ~50 | Pregnancy during  the pandemic of COVID‑19 | SAS;  50 | SDS;  50 | Pregnancy | 13 | 156 |
| Bante 2021^81^ | Ethiopia | Cross-sectional study | Community | January 01 to November 30, 2019 | 667 | 27.4 ±6 | Pregnancy | GAD-7;  5 | PHQ-9;  5 | NR | 67 | 667 |
| Jha 2021^82^ | India | Cross-sectional study | Community | May to December 2016 | 457 | 23.9±3.9 | Pregnancy | MINI | PRIME‑MD PHQ | 25 to 34 weeks pregnancy | 12 | 457 |
| Zhou 2021^83^ | China | Cross-sectional study | Community | July 2019 and September 2019 | 813 | 29.0±4.5  17 ~ 54 | Pregnancy | GAD-7;  10 | PHQ-9;  10 | Third trimester | 75 | 813 |
| Arshad 2021^84^ | Australia | Cohort study | Hospital | NR | 75 | 31.51±5.43  19 ~ 44 | Postpartum  CALD women | Previous diagnosis | Previous diagnosis | within 24 to 48 hours postpartum | 1 | 75 |
|  |  |  |  |  | 75 | 29.00±4.94  19 ~ 38 | Postpartum  non-CALD women. |  |  | within 24 to 48 hours postpartum | 5 | 75 |
| Cena 2021^85^ | Italy | Cross-sectional study | Hospital | 2017–2019 | 934 | ＞18 | Pregnancy | STAI;  40  EPDS-3Anxiety;  6 | EPDS;  10  PHQ-9;  10 | Third trimester  (27–40 weeks ) | 64 | 934 |
| Cheng 2021^86^ | China(Taiwan) | Longitudinal study | Hospital | May 2012 to September 2013 | 156 | 31.46±4.24 | Pregnancy | STAI;  40 | CES-D;  16 | 23–28 weeks pregnancy  (24.63 ±1.00 weeks) | 39 | 156 |
|  |  |  |  |  |  |  | Pregnancy |  |  | 32 to 36 weeks pregnancy | 42 | 147 |
|  |  |  |  |  |  |  | Pregnancy |  |  | over 36 weeks pregnancy | 40 | 129 |
|  |  |  |  |  |  |  | Postpartum |  |  | 4 to 6 weeks postpartum | 27 | 83 |
| Lilliecreutz 2021^87^ | Sweden | Case-control study | Hospital | 2013 | 2271 | NR | Pregnancy | PRIME-MD | EPDS;  13  PRIME-MD | First trimester  ( <14+0 weeks) | 22 | 2271 |
| Luo 2021^88^ | China | Cross-sectional study | Hospital | February 28, 2020 to April 26, 2020 | 2237 | 30.25±3.99  19 ~ 47 | Women during the COVID-19 pandemic | GAD-7;  7 | PHQ-9;  10 | Perinatal | 142 | 2237 |
| Nasreen 2021^89^ | Malaysia | Prospective cohort study | Hospital | March 2016 to August 2017 | 566 | NR | Postpartum | DASS 21;  8 | EPDS;  12 | 2 to 3 months postpartum | 9 | 566 |
| Stampini1 2021^90^ | Italy | Cross-sectional study | Online | April 9th, 2020 to  May 3rd, 2020 | 739 | 18 ~ 48 | Women during the COVID-19 pandemic | PHQ-4;  6 | PHQ-4;  6 | Perinatal | 464 | 739 |
| Mateus 2022^91^ | Multi-country | Prospective cohort study | Online | June 7th to October  31st 2020 | 3326 | 31.44±4.97 | Pregnancy | GAD-7;  10 | EPDS;  13 | Perinatal | 504 | 3326 |
|  | Brazil |  |  |  | 268 | NR |  |  |  | Pregnancy | 72 | 268 |
|  | Chile |  |  |  | 172 | NR |  |  |  | Pregnancy | 47 | 172 |
|  | Cyprus |  |  |  | 220 | NR |  |  |  | Pregnancy | 19 | 220 |
|  | Greece |  |  |  | 337 | NR |  |  |  | Pregnancy | 30 | 337 |
|  | Israel |  |  |  | 218 | NR |  |  |  | Pregnancy | 27 | 218 |
|  | Portugal |  |  |  | 753 | NR |  |  |  | Pregnancy | 115 | 753 |
|  | Spain |  |  |  | 353 | NR |  |  |  | Pregnancy | 69 | 353 |
|  | Turkey |  |  |  | 806 | NR |  |  |  | Pregnancy | 86 | 806 |
|  | UK |  |  |  | 199 | NR |  |  |  | Pregnancy | 39 | 199 |
|  | Multi-country |  |  |  | 3939 | 32.18±4.95 | Postpartum |  |  | <1 month  1 month  2 months  3 months  4 months  5 months  6 months | 799 | 3939 |
|  | Brazil |  |  |  | 597 | NR |  |  |  | Postpartum | 197 | 597 |
|  | Chile |  |  |  | 272 | NR |  |  |  | Postpartum | 76 | 272 |
|  | Cyprus |  |  |  | 249 | NR |  |  |  | Postpartum | 28 | 249 |
|  | Greece |  |  |  | 376 | NR |  |  |  | Postpartum | 66 | 376 |
|  | Israel |  |  |  | 335 | NR |  |  |  | Postpartum | 31 | 335 |
|  | Portugal |  |  |  | 669 | NR |  |  |  | Postpartum | 116 | 669 |
|  | Spain |  |  |  | 475 | NR |  |  |  | Postpartum | 113 | 475 |
|  | Turkey |  |  |  | 620 | NR |  |  |  | Postpartum | 91 | 620 |
|  | UK |  |  |  | 346 | NR |  |  |  | Postpartum | 81 | 346 |
| Zhou 2022^92^ | China | Cross-sectional study | Hospital | Early March to late September 2021 | 210 | 30.8±4.2  21 ~ 46 | Pregnancy  with twin pregnancy | SAS;  50 | SDS;  53 | Pregnancy | 51 | 210 |
| Ray 2022^93^ | India | Cross-sectional study | Hospital | March 2020 to July 2021 | 124 | 30.6±4.13  22 ~ 40 | Pregnancy or had delivered within 1 year in the COVID-19 Pandemic | GAD-7;  10 | PHQ-9;  10 | Perinatal | 23 | 124 |
| Abdus-salam 2022^94^ | Nigeria | Cross-sectional study | Hospital | August to November 2020 | 380 | 32±4.78 | pregnancy in Covid-19 | HADS;  NR-cut off value | HADS;  NR-cut off value | Pregnancy | 15 | 380 |
| Motrico 2022^95^ | Spain | Cross-sectional study | Online | 15 June to 31 December 2020 | 3356 | 33.7±4.3 | Perinatal in Covid-19 | GAD-7;  10 | EPDS;  10 | Perinatal | 980 | 3356 |
| Avalos 2022^96^ | American | Cross-sectional study | Hospital | 22 June and 30 September 2020 | 6592 | NR | COVID-19 during pregnancy | GAD-7;  5 | PHQ-8;  5 | Pregnancy | 1559 | 6236 |
| Esquivel 2022^97^ | Cuba | Longitudinal cohort study | Hospital | January to June 2017 | 281 | NR | Postpartum | STAI;  40 | EPDS;  9 | 4 weeks postpartum | 16 | 274 |
|  |  |  |  |  |  |  | Postpartum |  |  | 12 weeks postpartum | 9 | 261 |
|  |  |  |  |  |  |  | Postpartum |  |  | 24 weeks postpartum | 8 | 243 |
| IRUM 2022^98^ | Pakistan | Cross-Sectional Study | Hospital | From April 2019 to August 2019 | 200 | 26.7 ± 5.0 | Pregnancy | HADS;  8 | HADS;  8 | Pregnancy | 100 | 200 |
| Ma 2022^99^ | China | Prospective cohort study | Hospital | January to September ,2008 | 1583 | ≥20 | Pregnancy | SAS;  50 | CES-D;  16 | 30 to 34 weeks pregnancy | 142 | 1583 |
| Malaju 2022^100^ | Ethiopia | Longitudinal study | Hospital | October, 2020 to March, 2021 | 775 | 26.33±4.355 | Postpartum | DASS-21;  8 | DASS-21;  10 | 6 weeks postpartum | 112 | 775 |
|  |  |  |  |  |  |  |  |  |  | 12 weeks postpartum | 94 | 775 |
|  |  |  |  |  |  |  |  |  |  | 18 weeks postpartum | 63 | 775 |
| Odinka 2022^101^ | Nigeria | Cross-sectional study | Hospital | November to December 2015 | 309 | 29.65±4.87  20 ~ 46 | Postpartum | HADS;  11 | HADS;  11 | 6 to 14 weeks postpartum | 74 | 309 |
| Yang 2023^102^ | China | Cross-sectional study | Hospital | August 2021 to February 2022 | 1963 | 29.81 ± 4.51  18 ~ 42 | Pregnant women in the post-COVID-19 era | GAD-7;  5 | PHQ-9;  5 | Pregnancy | 365 | 1963 |
| Hannon 2023^103^ | Ireland | Cohort study | Hospital | January 2012 to March 2017 | 1804 | NR | Nulliparous | DASS 21;  6 | DASS 21;  7 | Pregnancy | 27 | 1804 |
|  |  |  |  |  |  |  |  |  |  | 3 months postpartum | 29 | 1804 |
|  |  |  |  |  |  |  |  |  |  | 6 months postpartum | 41 | 1804 |
|  |  |  |  |  |  |  |  |  |  | 9 months postpartum | 32 | 1804 |
|  |  |  |  |  |  |  |  |  |  | 12 months postpartum | 43 | 1804 |
| Yang 2023^104^ | China | Longitudinal study | NR | NR | 457 | 28.48±3.223 | Pregnancy | HADS;  7 | HADS;  7 | Pregnancy | 39 | 457 |
| Craemer 2023^105^ | American | Cross-sectional study | Hospital | July 2019 to March 2022 | 717 | NR | Perinatal | Computerized adaptive diagnostic tool CAT-MH®; | Computerized adaptive diagnostic tool CAT-MH®; | Perinatal | 9 | 717 |
| Hou 2023^106^ | China | Cohort study | Hospital | May 2019 to December 2019 | 1082 | 28.69 ±4.01 | Pregnancy | GAD-7;  5 | EPDS;  9 | First trimester | 252 | 1082 |
|  |  |  |  |  |  |  | Pregnancy |  |  | 15 to 27 weeks pregnancy | 50 | 926 |
|  |  |  |  |  |  |  | Pregnancy |  |  | 28 to 40 weeks pregnancy | 35 | 757 |
|  |  |  |  |  |  |  | Postpartum |  |  | Within 1 year postpartum | 49 | 685 |
| Tambawala 2023^107^ | United Arab  Emirates | Cross-sectional study | Hospital | NR | 438 | 31.79 (mean)  19 ~52 | During the COVID-19 pandemic | GAD-7;  5 | EPDS;  10 | Perinatal | 142 | 438 |
| Bertram 2023^108^ | Australia | Cohort study | Database | 2013 to 2022 | 66 945 | 31.0 (24–38) | Perinatal | Recorded-anxiety;  NA | Recorded-depression;  NA | Perinatal | 4638 | 49598 |
| Dadhwal 2023^109^ | India | Cross-sectional study | Community | June 2014 to June 2016 | 680 | 18-40 | Postpartum | STAI;  40 | EPDS;  10 | 4 weeks to 6 months postpartum | 43 | 680 |
| Gennaro 2023^110^ | US | Cross-sectional study | Hospital | March 2018 to March 2022. | 452 | 26.7 ±5.15 | Pregnancy | GAD-7;  10 | EPDS;  10 | Pregnancy | 7 | 194 |
| Costa 2024^111^ | Multi-country | Cross-sectional study | Online | June 2020 to  April 2021 | 11809 | 25-35 | During the COVID-19 pandemic | GAD-7;  10 | EPDS;  13 | Perinatal | 2303 | 11809 |
| Fijean 2024^112^ | French | Cross-sectional study | Hospital | April 2020 and June 2023 | 923 | NR | During the COVID-19 pandemic | STAI-EPDS-3ANXIETY;  10 | EPDS;  10 | 2 months postpartum | 109 | 508 |
| Altendahl 2024^113^ | US | Cohort study | Online | March 2020 to October 2020 | 317 | 31.5 ±4.91 | During the COVID-19 pandemic | GAD-7;  10 | EPDS;  13 | Second trimester | 15 | 174 |
|  |  |  |  |  |  |  |  |  |  | Third trimester | 27 | 301 |
|  |  |  |  |  |  |  |  |  |  | 6 to 8 weeks postpartum | 21 | 311 |
|  |  |  |  |  |  |  |  |  |  | 6 months postpartum | 28 | 243 |
| Eriksson 2024^114^ | Sweden | Cross-sectional study | Hospital | 2014 to 2018 | 112 | NR | Postpartum | BAI;  Definite level (16) | EPDS;  Definite level (11) | 6 weeks postpartum | 9 | 112 |
| Bain 2024^115^ | Pakistan | Experimental study | Hospital | April 2019 to October 2022 | 18318 | NR | During the COVID-19 pandemic | HADS;  8 | HADS;  8 | Pregnancy | 62 | 18318 |
| Bante 2024^116^ | Ethiopia | Cross-sectional study | Community | February to March 2022. | 451 | 29.08 ±5.48 | During the COVID-19 pandemic | DASS-21;  mild ( 8–9 ), moderate (10–14 ), severe ( 15–19), extremely severe (＞20) | DASS-21;  mild (10–13 ), moderate (14–20 ), severe (21–27), and extremely severe ( ＞28 ) | 6 to 12 months postpartum | 50 | 449 |
| Chen 2024^117^ | China | Cohort study | Hospital | January 2019 to January 2022 | 648 | NR | Postpartum | SAS;  50 | SDS;  53 | Postpartum | 234 | 648 |
| Haile 2024^118^ | Ethiopia | Cross-sectional study | Community | 1 July and 30 August 2021 | 872 | M=29  (IQR: 33, 26) | During the COVID-19 pandemic | PRA;  13 | PHQ-9;  10 | Pregnancy | 56 | 858 |
| Shen 2024^119^ | China | Longitudinal study | Database | March 22, 2017 and March 30, 2022 | 1941 | Mean=30.92  18-47 | Perinatal | SAS;  50 | SDS;  50 | First trimester | 304 | 1941 |
|  |  |  |  |  |  |  |  |  |  | Second trimester | 157 | 1878 |
|  |  |  |  |  |  |  |  |  |  | Third trimester | 226 | 1941 |
|  |  |  |  |  |  |  |  |  |  | Postpartum | 252 | 1901 |
| Zheng 2024^120^ | China | Longitudinal study | Database | July 2017 to July 2021 | 2876 | 31.3±3.9 | Pregnancy | SAS;  50 | CESD;  15 | Pregnancy | 190 | 2876 |
|  |  |  |  |  |  |  |  |  |  | First trimester | 305 | 2876 |
|  |  |  |  |  |  |  |  |  |  | Second trimester | 103 | 2876 |
|  |  |  |  |  |  |  |  |  |  | Third trimester | 159 | 2876 |
| Jin 2024^121^ | China | Cohort study | Hospital | October 2022 to November 2023. | 549 | 30±4.81 | Pregnancy | SAS;  50 | EPDS;  13 | Pregnancy | 1 | 549 |
| Ma 2024^122^ | China | Cohort study | Hospital | January 2021  to October 2023. | 80 | 18-35 | Third trimester | HADS;  7 | HADS;  7 | Third trimester | 20 | 80 |

AKUADS, Aga Khan University Anxiety and Depression Scale

AUDADIS-IV, Disorder and Associated Disabilities Interview Schedule-DSM-IV

BAI, Beck Anxiety Inventory

BSI, Brief Symptom Index

CESD, Center for Epidemiological Studies-Depression scale

CIDI, Composite International Diagnostic Interview

CIS-R, Clinical Interview Schedule - Revised Version

DASS, Depression, Anxiety and Stress Scales

EPDS, Edinburgh Postnatal Depression Scale

GAD, Generalized Anxiety Disorder scale

HADS, Hospital Anxiety and Depression Scales

HARS, Hamilton Anxiety rating scale

HSCL, Hopkins Symptom Checklist

MINI, Mini International Neuropsychiatric Interview

PASS, Perinatal Anxiety Screening Scale

PHQ, Patient Health Questionnaire

PRA, Pregnancy-related anxiety scale

PRIME-MD, Primary Care Evaluation of Mental Disorders

SAS, Self-Rating Anxiety Scale

SCID, Structured Clinical Interview

SCL, Symptom Check List

SDS, Self-Rating Depression Scale

SRAS, Zung’s Self Rating Anxiety Scale

STAI, State-Trait Anxiety Inventory

STAI-S, Spielberger State Anxiety Inventory

STAI-T, Spielberger Trait Anxiety Inventory

**Online Supplementary Table S3. Quality assessment of the studies**

| **Studies** | **Representation** | **Target population** | **Random selection** | **Nonresponse bias** | **Data collected from subjects** | **Acceptable case definition** | **Study instrument**  **(if applicable)** | **Mode of data collection** | **Length** | **Numerator and denominator** | **Total** |
| --- | --- | --- | --- | --- | --- | --- | --- | --- | --- | --- | --- |
| Sutter-Dallay 2004 | higher risk | low risk | high risk | high risk | low risk | low risk | low risk | low risk | low risk | low risk | moderate |
| Adewuya 2005 | high risk | low risk | high risk | low risk | low risk | high risk | low risk | low risk | low risk | low risk | moderate |
| Andersson 2006 | high risk | low risk | high risk | low risk | low risk | low risk | low risk | low risk | low risk | high risk | moderate |
| Miller 2006 | high risk | high risk | high risk | low risk | low risk | low risk | low risk | low risk | low risk | low risk | moderate |
| Lee 2007 | high risk | low risk | high risk | low risk | low risk | low risk | low risk | low risk | low risk | low risk | low |
| Phillips 2007 | high risk | low risk | high risk | high risk | low risk | low risk | low risk | low risk | low risk | low risk | moderate |
| Sato 2008 | high risk | low risk | high risk | high risk | low risk | low risk | low risk | low risk | low risk | low risk | moderate |
| Rowe 2008 | high risk | low risk | high risk | low risk | low risk | low risk | low risk | low risk | low risk | low risk | low |
| Reck 2008 | high risk | low risk | high risk | low risk | low risk | low risk | low risk | low risk | low risk | low risk | low |
| Faisal-Cury 2009 | high risk | low risk | high risk | low risk | low risk | low risk | high risk | low risk | low risk | high risk | low |
| Ali et al., 2009 | low risk | low risk | high risk | high risk | low risk | low risk | low risk | low risk | low risk | low risk | low |
| Giakoumaki 2009 | high risk | low risk | high risk | high risk | low risk | low risk | low risk | low risk | low risk | low risk | moderate |
| Qiao 2009 | high risk | low risk | high risk | low risk | low risk | low risk | low risk | low risk | low risk | low risk | low |
| Teixeira 2009 | high risk | low risk | low risk | low risk | low risk | low risk | low risk | low risk | low risk | low risk | low |
| Batenburg-Eddes 2009 | high risk | low risk | high risk | high risk | low risk | low risk | low risk | low risk | low risk | low risk | moderate |
| Woolhouse 2009 | high risk | low risk | high risk | low risk | low risk | low risk | low risk | low risk | low risk | low risk | low |
| Austin 2010 | high risk | low risk | high risk | high risk | low risk | high risk | low risk | low risk | low risk | low risk | moderate |
| Goodman 2010 | high risk | low risk | high risk | high risk | low risk | low risk | low risk | low risk | low risk | low risk | moderate |
| Yelland 2010 | high risk | low risk | high risk | high risk | low risk | low risk | low risk | low risk | low risk | low risk | moderate |
| Fisher 2010 | high risk | low risk | low risk | low risk | low risk | low risk | low risk | low risk | low risk | low risk | low |
| Field 2010 | high risk | low risk | high risk | low risk | low risk | low risk | low risk | low risk | low risk | low risk | low |
| Edhborg 2011 | high risk | low risk | high risk | low risk | low risk | low risk | low risk | low risk | low risk | low risk | low |
| Figueiredo 2011 | high risk | low risk | low risk | high risk | low risk | low risk | low risk | low risk | low risk | low risk | low |
| Le Strat et al. 2011 | low risk | low risk | high risk | low risk | low risk | low risk | low risk | low risk | low risk | low risk | low |
| Paul 2012 | high risk | low risk | high risk | low risk | low risk | low risk | low risk | low risk | low risk | low risk | low |
| Tavares 2012 | high risk | low risk | high risk | low risk | low risk | low risk | low risk | low risk | low risk | low risk | low |
| Grant 2012 | high risk | low risk | high risk | high risk | low risk | low risk | low risk | low risk | low risk | low risk | moderate |
| Ali 2012 | high risk | low risk | high risk | low risk | low risk | low risk | low risk | low risk | low risk | low risk | low |
| Storksen 2012 | high risk | low risk | high risk | high risk | low risk | low risk | low risk | low risk | low risk | low risk | moderate |
| Bener 2012 | high risk | low risk | high risk | low risk | low risk | low risk | low risk | low risk | low risk | low risk | low |
| Ban et al. 2012 | low risk | low risk | low risk | low risk | low risk | low risk | high risk | low risk | low risk | low risk | low |
| Ibanez 2012 | high risk | low risk | high risk | low risk | low risk | low risk | low risk | low risk | low risk | low risk | low |
| Bindt 2013 | high risk | low risk | high risk | high risk | low risk | low risk | low risk | low risk | low risk | low risk | moderate |
| Wynter 2013 | high risk | low risk | high risk | high risk | low risk | low risk | low risk | low risk | low risk | low risk | moderate |
| Makara-Studzińska 2013 | high risk | low risk | high risk | low risk | low risk | low risk | low risk | low risk | low risk | low risk | low |
| Thiagayson 2013 | high risk | low risk | high risk | low risk | low risk | low risk | low risk | low risk | low risk | low risk | low |
| Bjørk et al.2014 | low risk | low risk | high risk | low risk | low risk | low risk | low risk | low risk | low risk | low risk | low |
| Enatescu 2014 | high risk | low risk | high risk | low risk | low risk | low risk | low risk | low risk | low risk | low risk | low |
| Farr 2014 | high risk | low risk | low risk | low risk | low risk | low risk | low risk | low risk | low risk | low risk | low |
| Miller 2014 | high risk | low risk | high risk | low risk | low risk | low risk | low risk | low risk | low risk | low risk | low |
| Koutra 2014 | high risk | low risk | high risk | low risk | low risk | low risk | low risk | low risk | low risk | low risk | low |
| Segre 2014 | high risk | low risk | high risk | low risk | low risk | low risk | low risk | low risk | low risk | low risk | low |
| Simpson 2014 | high risk | low risk | high risk | low risk | low risk | low risk | low risk | low risk | low risk | low risk | low |
| Tendais 2014 | high risk | low risk | high risk | high risk | low risk | low risk | low risk | low risk | low risk | low risk | moderate |
| Nguyen 2015 | high risk | low risk | low risk | low risk | low risk | low risk | low risk | low risk | low risk | low risk | low |
| Cheng 2015 | high risk | low risk | high risk | low risk | low risk | low risk | low risk | low risk | low risk | low risk | low |
| Martini 2015 | high risk | low risk | high risk | low risk | low risk | low risk | low risk | low risk | low risk | low risk | low |
| Abdelhai 2015 | high risk | low risk | low risk | low risk | low risk | low risk | low risk | low risk | low risk | low risk | low |
| Waldie 2015 | high risk | low risk | high risk | low risk | low risk | high risk | high risk | low risk | low risk | low risk | moderate |
| Falah-Hassani 2016 | high risk | low risk | high risk | low risk | low risk | low risk | low risk | low risk | low risk | low risk | low |
| Tham 2016 | high risk | low risk | high risk | low risk | low risk | low risk | low risk | low risk | low risk | low risk | low |
| Venkatesh 2016 | high risk | low risk | high risk | low risk | low risk | low risk | low risk | low risk | low risk | high risk | moderate |
| Chen 2016 | high risk | low risk | high risk | low risk | low risk | low risk | low risk | low risk | low risk | low risk | low |
| Dikmen-Yildiz 2017 | high risk | low risk | high risk | low risk | low risk | low risk | low risk | low risk | low risk | low risk | low |
| Nasreen 2018 | high risk | low risk | high risk | low risk | low risk | low risk | low risk | low risk | low risk | low risk | low |
| Odinka 2018 | high risk | low risk | low risk | low risk | low risk | low risk | low risk | low risk | low risk | low risk | low |
| Aryal 2018 | high risk | low risk | high risk | low risk | low risk | low risk | low risk | low risk | low risk | low risk | low |
| Dennis 2018 | high risk | low risk | high risk | low risk | low risk | low risk | low risk | low risk | low risk | low risk | low |
| Dennis 2018 | high risk | low risk | high risk | low risk | low risk | low risk | low risk | low risk | low risk | low risk | low |
| Redinger 2018 | high risk | low risk | high risk | low risk | low risk | low risk | low risk | low risk | low risk | low risk | low |
| Suzuki 2018 | high risk | low risk | high risk | low risk | low risk | low risk | low risk | low risk | low risk | low risk | low |
| Nakić Radoš 2018 | high risk | low risk | high risk | low risk | low risk | low risk | low risk | low risk | low risk | low risk | low |
| González-Hernández 2019 | high risk | low risk | high risk | low risk | low risk | low risk | low risk | low risk | low risk | low risk | low |
| Agbaje 2019 | high risk | low risk | high risk | low risk | low risk | low risk | low risk | low risk | low risk | low risk | low |
| Genovez 2019 | high risk | low risk | high risk | low risk | low risk | low risk | low risk | low risk | low risk | low risk | low |
| Aris-Meijer 2019 | low risk | high risk | low risk | low risk | low risk | low risk | low risk | low risk | low risk | low | Low |
| Samwel Ngocho 2019 | high risk | low risk | high risk | low risk | low risk | low risk | low risk | low risk | low risk | low risk | low |
| Tay 2019 | low risk | low risk | low risk | high risk | low risk | high risk | high risk | low risk | high risk | low risk | moderate |
| Uguz 2019 | high risk | low risk | high risk | low risk | low risk | low risk | low risk | low risk | low risk | low risk | low |
| Gelman 2019 | high risk | low risk | high risk | low risk | low risk | high risk | high risk | low risk | low risk | low risk | moderate |
| Ramakrishna 2019 | high risk | low risk | high risk | low risk | low risk | low risk | low risk | low risk | low risk | low risk | low |
| Wassif 2019 | high risk | low risk | low risk | low risk | low risk | low risk | low risk | low risk | low risk | low risk | low |
| Redinger 2020 | high risk | low risk | high risk | low risk | low risk | low risk | low risk | low risk | low risk | low risk | low |
| Umuziga 2020 | high risk | low risk | low risk | low risk | low risk | low risk | low risk | low risk | low risk | low risk | low |
| González-Mesa 2020 | high risk | low risk | high risk | high risk | low risk | high risk | low risk | low risk | low risk | low risk | moderate |
| Premji 2020 | high risk | low risk | high risk | low risk | low risk | low risk | low risk | low risk | low risk | low risk | low |
| Kantipudi 2020 | high risk | low risk | high risk | low risk | low risk | low risk | low risk | low risk | low risk | low risk | low |
| Pabon 2020 | high risk | low risk | high risk | low risk | low risk | low risk | low risk | low risk | low risk | low risk | low |
| Coo 2021 | high risk | low risk | high risk | high risk | low risk | low risk | low risk | low risk | low risk | low risk | moderate |
| Dong 2021 | high risk | low risk | high risk | low risk | low risk | low risk | low risk | low risk | low risk | low risk | low |
| Bante 2021 | high risk | low risk | low risk | low risk | low risk | low risk | low risk | low risk | low risk | low risk | low |
| Jha 2021 | high risk | low risk | high risk | low risk | low risk | low risk | low risk | low risk | low risk | low risk | low |
| Zhou 2021 | high risk | low risk | low risk | low risk | low risk | low risk | low risk | low risk | low risk | low risk | low |
| Arshad 2021 | high risk | low risk | high risk | low risk | low risk | low risk | low risk | low risk | high risk | low risk | moderate |
| Cena 2021 | high risk | low risk | high risk | low risk | low risk | low risk | low risk | low risk | low risk | low risk | low |
| Cheng 2021 | high risk | low risk | high risk | high risk | low risk | low risk | low risk | low risk | low risk | low risk | moderate |
| Lilliecreutz 2021 | high risk | low risk | high risk | low risk | low risk | low risk | low risk | low risk | low risk | low risk | low |
| Luo 2021 | high risk | low risk | high risk | low risk | low risk | low risk | low risk | low risk | low risk | low risk | low |
| Nasreen 2021 | high risk | low risk | high risk | high risk | low risk | low risk | low risk | low risk | low risk | low risk | moderate |
| Stampini1 2021 | high risk | low risk | high risk | low risk | low risk | low risk | low risk | low risk | low risk | low risk | low |
| Ray 2022 | high risk | low risk | low risk | low risk | low risk | low risk | low risk | low risk | low risk | low risk | low |
| Mateus 2022 | high risk | low risk | high risk | high risk | low risk | low risk | low risk | low risk | low risk | low risk | moderate |
| Zhou 2022 | high risk | low risk | high risk | low risk | low risk | low risk | low risk | low risk | low risk | low risk | low |
| Abdus-salam 2022 | high risk | low risk | low risk | low risk | low risk | low risk | low risk | low risk | low risk | low risk | low |
| Motrico 2022 | high risk | low risk | high risk | low risk | low risk | low risk | low risk | low risk | low risk | low risk | low |
| Avalos 2022 | high risk | low risk | high risk | low risk | low risk | low risk | low risk | low risk | low risk | low risk | low |
| Esquivel 2022 | high risk | low risk | high risk | low risk | low risk | low risk | low risk | low risk | low risk | low risk | low |
| IRUM 2022 | high risk | low risk | high risk | low risk | low risk | low risk | low risk | low risk | low risk | low risk | low |
| Ma 2022 | high risk | low risk | high risk | low risk | low risk | low risk | low risk | low risk | low risk | low risk | low |
| Malaju 2022 | high risk | low risk | high risk | low risk | low risk | low risk | low risk | low risk | low risk | low risk | low |
| Odinka 2022 | high risk | low risk | low risk | low risk | low risk | low risk | low risk | low risk | low risk | low risk | low |
| Yang 2023 | high risk | low risk | high risk | low risk | low risk | low risk | low risk | low risk | low risk | low risk | low |
| Hannon 2023 | high risk | low risk | high risk | high risk | low risk | low risk | low risk | low risk | low risk | low risk | moderate |
| Yang 2023 | high risk | low risk | high risk | low risk | low risk | low risk | low risk | low risk | low risk | low risk | low |
| Craemer 2023 | high risk | low risk | high risk | low risk | low risk | low risk | low risk | low risk | low risk | low risk | low |
| Hou 2023 | high risk | low risk | high risk | low risk | low risk | low risk | low risk | low risk | low risk | low risk | low |
| Tambawala 2023 | high risk | low risk | high risk | low risk | low risk | low risk | low risk | low risk | low risk | low risk | low |
| Bertram 2023 | low risk | low risk | high risk | low risk | low risk | low risk | low risk | low risk | low risk | low risk | low |
| Dadhwal 2023 | high risk | low risk | high risk | low risk | low risk | low risk | low risk | low risk | low risk | low risk | low |
| Gennaro 2023 | high risk | low risk | high risk | low risk | low risk | low risk | low risk | low risk | low risk | low risk | low |
| Costa 2024 | low risk | low risk | high risk | low risk | low risk | low risk | low risk | low risk | low risk | low risk | low |
| Fijean 2024 | high risk | low risk | high risk | high risk | low risk | low risk | low risk | low risk | low risk | low risk | moderate |
| Altendahl 2024 | high risk | low risk | high risk | high risk | low risk | low risk | low risk | low risk | low risk | low risk | moderate |
| Eriksson 2024 | high risk | low risk | high risk | high risk | low risk | low risk | low risk | low risk | low risk | low risk | moderate |
| Bain 2024 | high risk | low risk | high risk | low risk | low risk | low risk | low risk | low risk | low risk | low risk | low |
| Bante 2024 | high risk | low risk | low risk | low risk | low risk | low risk | low risk | low risk | low risk | low risk | low |
| Chen 2024 | high risk | low risk | high risk | low risk | low risk | low risk | low risk | low risk | low risk | low risk | low |
| Haile 2024 | high risk | low risk | high risk | low risk | low risk | low risk | low risk | low risk | low risk | low risk | low |
| Shen 2024 | high risk | low risk | high risk | low risk | low risk | low risk | low risk | low risk | low risk | low risk | low |
| Zheng 2024 | high risk | low risk | high risk | low risk | low risk | low risk | low risk | low risk | low risk | low risk | low |
| Jin 2024 | high risk | low risk | high risk | low risk | low risk | low risk | low risk | low risk | low risk | low risk | low |

Online Supplementary Table S4. Meta-regression results

| **Meta regression** | **Coefficient(β)** | **Standard Error** | **Lower 95%CI** | **Upper 95%CI** | **P** | **Overall R^2^** | **Overall P** |
| --- | --- | --- | --- | --- | --- | --- | --- |
| **Publication year** |  |  |  |  |  | 0.00% | 0.4131 |
| Before 2016 | Ref |  |  |  |  |  |  |
| 2016-2024 | 0.157 | 0.1488 | -0.1345 | 0.4486 | 0.2911 |  |  |
| **Covid-19 context** |  |  |  |  |  | 0.00% | 0.3518 |
| No | Ref |  |  |  |  |  |  |
| Yes | -0.3722 | 0.1835 | -0.7318 | -0.0126 | 0.0425 |  |  |
| **Time points** |  |  |  |  |  | 9.68% | 0.0012 |
| The first trimester | Ref |  |  |  |  |  |  |
| The second trimester | -0.5532 | 0.3242 | -1.1887 | 0.0823 | 0.088 |  |  |
| The third trimester | -0.4654 | 0.2918 | -1.0373 | 0.1065 | 0.1107 |  |  |
| Within 1 week postpartum | -0.1261 | 0.4733 | -1.0538 | 0.8016 | 0.79 |  |  |
| 1 week to 1 month postpartum | -0.6931 | 0.4427 | -1.5608 | 0.1746 | 0.1174 |  |  |
| 1 month to 2 month postpartum | -0.4165 | 0.3469 | -1.0965 | 0.2634 | 0.2299 |  |  |
| 2 month to 4 month postpartum | -0.9973 | 0.3338 | -1.6514 | -0.3431 | 0.0028 |  |  |
| 4 month to 6 month postpartum | -0.8745 | 0.358 | -1.5763 | -0.1727 | 0.0146 |  |  |
| 6 month to 12 month postpartum | -1.3548 | 0.3481 | -2.0371 | -0.6725 | <.0001 |  |  |
| Pregnancy (not specified) | -0.7249 | 0.2819 | -1.2773 | -0.1724 | 0.0101 |  |  |
| Postpartum (not specified) | -0.0332 | 0.3107 | -0.6422 | 0.5759 | 0.915 |  |  |
| Perinatal (not specified) | -0.2923 | 0.3378 | -0.9545 | 0.3698 | 0.3869 |  |  |
| **Design** |  |  |  |  |  | 8.09% | 0.0006 |
| Cross-sectional study | Ref |  |  |  |  |  |  |
| Cohort study | -0.8001 | 0.2382 | -1.2671 | -0.3332 | 0.0008 |  |  |
| Longitudinal study | -0.428 | 0.1703 | -0.7617 | -0.0943 | 0.012 |  |  |
| Prospective cohort study | -0.6137 | 0.2217 | -1.0482 | -0.1792 | 0.0056 |  |  |
| Quasi-experimental study | -0.4993 | 0.4738 | -1.4279 | 0.4294 | 0.292 |  |  |
| Retrospective cohort study | -1.9961 | 0.651 | -3.272 | -0.7201 | 0.0022 |  |  |
| Secondary analysis of RCT | -1.2397 | 0.9137 | -3.0305 | 0.551 | 0.1748 |  |  |
| Case control study | -0.7051 | 0.5613 | -1.8052 | 0.3951 | 0.2091 |  |  |
| **Country** |  |  |  |  |  | 1.84% | 0.0952 |
| High-income countries | Ref |  |  |  |  |  |  |
| Middle-income countries | 0.3775 | 0.1621 | 0.0597 | 0.6953 | 0.0199 |  |  |
| Low-income countries | 0.3548 | 0.2078 | -0.0524 | 0.7621 | 0.0877 |  |  |
| **Country (detailed)** |  |  |  |  |  | 34.28% | <.0001 |
| Egypt | Ref |  |  |  |  |  |  |
| Australia | -2.0535 | 0.401 | -2.8394 | -1.2675 | <.0001 |  |  |
| Bangladesh | -2.7258 | 0.8433 | -4.3787 | -1.0729 | 0.0012 |  |  |
| Brazil | -0.9867 | 0.4746 | -1.9169 | -0.0566 | 0.0376 |  |  |
| Canada | -1.171 | 0.44 | -2.0333 | -0.3087 | 0.0078 |  |  |
| Chile | -1.307 | 0.4831 | -2.2538 | -0.3602 | 0.0068 |  |  |
| China | -1.6825 | 0.371 | -2.4095 | -0.9554 | <.0001 |  |  |
| Croatia | -1.2162 | 0.8303 | -2.8435 | 0.4111 | 0.143 |  |  |
| Cuba | -2.5444 | 0.574 | -3.6694 | -1.4194 | <.0001 |  |  |
| Cyprus | -1.6634 | 0.6408 | -2.9195 | -0.4074 | 0.0094 |  |  |
| Ethiopia | -1.5599 | 0.5043 | -2.5484 | -0.5714 | 0.002 |  |  |
| France | -2.1339 | 0.6398 | -3.3879 | -0.8798 | 0.0009 |  |  |
| Germany | -2.0249 | 0.6366 | -3.2725 | -0.7773 | 0.0015 |  |  |
| Ghana and Côte D'Ivoire | -1.5924 | 0.8252 | -3.2098 | 0.025 | 0.0536 |  |  |
| Greece | -0.9451 | 0.4407 | -1.8088 | -0.0814 | 0.032 |  |  |
| India | -1.7626 | 0.5604 | -2.861 | -0.6642 | 0.0017 |  |  |
| Ireland | -3.3234 | 0.4792 | -4.2625 | -2.3842 | <.0001 |  |  |
| Israel | -1.5828 | 0.6373 | -2.832 | -0.3337 | 0.013 |  |  |
| Italy | -0.9076 | 0.6281 | -2.1387 | 0.3236 | 0.1485 |  |  |
| Japan | -2.429 | 0.4773 | -3.3646 | -1.4934 | <.0001 |  |  |
| Malaysia | -2.3685 | 0.5612 | -3.4685 | -1.2685 | <.0001 |  |  |
| Mexico | -0.6957 | 0.6323 | -1.935 | 0.5436 | 0.2712 |  |  |
| Nepal | -1.3942 | 0.6348 | -2.6384 | -0.1501 | 0.0281 |  |  |
| The Netherlands | -3.6232 | 0.5512 | -4.7036 | -2.5427 | <.0001 |  |  |
| New Zealand | -3.6323 | 0.8257 | -5.2508 | -2.0139 | <.0001 |  |  |
| Nigeria | -1.7292 | 0.4136 | -2.5398 | -0.9186 | <.0001 |  |  |
| Norway | -2.2524 | 0.6276 | -3.4824 | -1.0224 | 0.0003 |  |  |
| Pakistan | -1.6541 | 0.4152 | -2.4678 | -0.8403 | <.0001 |  |  |
| Poland | -1.4709 | 0.553 | -2.5548 | -0.3871 | 0.0078 |  |  |
| Portugal | -1.6528 | 0.434 | -2.5035 | -0.8022 | 0.0001 |  |  |
| Qatar | -2.6189 | 0.8273 | -4.2404 | -0.9973 | 0.0015 |  |  |
| Romania | -0.0412 | 0.8257 | -1.6595 | 1.5771 | 0.9602 |  |  |
| Rwanda | -0.5027 | 0.8261 | -2.1218 | 1.1163 | 0.5428 |  |  |
| Singapore | -1.6374 | 0.5098 | -2.6366 | -0.6383 | 0.0013 |  |  |
| South Africa | -1.8606 | 0.5506 | -2.9397 | -0.7814 | 0.0007 |  |  |
| Spain | -1.0014 | 0.5049 | -1.991 | -0.0118 | 0.0473 |  |  |
| Sweden | -1.2239 | 0.5552 | -2.3121 | -0.1357 | 0.0275 |  |  |
| Tanzania | -1.0628 | 0.8319 | -2.6933 | 0.5676 | 0.2014 |  |  |
| Turkey | -1.4385 | 0.4398 | -2.3005 | -0.5764 | 0.0011 |  |  |
| UK | -2.261 | 0.5035 | -3.2478 | -1.2742 | <.0001 |  |  |
| United ArabEmirates | -0.4744 | 0.821 | -2.0835 | 1.1346 | 0.5633 |  |  |
| US | -2.0325 | 0.3983 | -2.8132 | -1.2518 | <.0001 |  |  |
| Vietnam | -2.6657 | 0.5854 | -3.8131 | -1.5182 | <.0001 |  |  |
| **Measurement tool for anxiety** |  |  |  |  |  | 14.59% | 0.0014 |
| HADS | Ref |  |  |  |  |  |  |
| AKUADS | -0.3217 | 0.4775 | -1.2576 | 0.6142 | 0.5005 |  |  |
| AUDADIS-IV | -0.7489 | 0.8949 | -2.5028 | 1.0051 | 0.4027 |  |  |
| Auto-CIDI | -1.5708 | 0.9068 | -3.3482 | 0.2066 | 0.0832 |  |  |
| BAI | 0.515 | 0.5435 | -0.5502 | 1.5802 | 0.3433 |  |  |
| BSI | -2.0337 | 0.8954 | -3.7887 | -0.2787 | 0.0231 |  |  |
| BSI-18 | 0.5087 | 0.9008 | -1.2568 | 2.2742 | 0.5723 |  |  |
| CIDI | -0.2512 | 0.5921 | -1.4116 | 0.9092 | 0.6714 |  |  |
| CIS-R | 0.3607 | 0.8917 | -1.3871 | 2.1085 | 0.6859 |  |  |
| Computerized adaptive diagnostic tool CAT-MH® | -2.1544 | 0.9478 | -4.0121 | -0.2966 | 0.023 |  |  |
| DASS21 | -0.8861 | 0.2856 | -1.4458 | -0.3263 | 0.0019 |  |  |
| DASS42 | 0.6723 | 0.8922 | -1.0764 | 2.4211 | 0.4511 |  |  |
| GAD-2 | 0.3883 | 0.8944 | -1.3646 | 2.1412 | 0.6642 |  |  |
| GAD-7 | 0.1327 | 0.2382 | -0.3342 | 0.5996 | 0.5775 |  |  |
| HARS | 0.8717 | 0.645 | -0.3924 | 2.1358 | 0.1765 |  |  |
| HSCL25 | 0.1779 | 0.6474 | -1.091 | 1.4468 | 0.7835 |  |  |
| MINI | -0.8663 | 0.5453 | -1.9351 | 0.2025 | 0.1121 |  |  |
| PASS | -0.2353 | 0.552 | -1.3171 | 0.8466 | 0.6699 |  |  |
| PHQ-4 | 1.7581 | 0.8885 | 0.0166 | 3.4995 | 0.0479 |  |  |
| PHQ-ANXIETY | -1.326 | 0.6745 | -2.6481 | -0.0039 | 0.0493 |  |  |
| PRA | -0.5866 | 0.4333 | -1.4359 | 0.2626 | 0.1758 |  |  |
| PRIME-MD | 0.346 | 0.5385 | -0.7095 | 1.4014 | 0.5206 |  |  |
| Recorded anxiety | -0.9354 | 0.424 | -1.7664 | -0.1043 | 0.0274 |  |  |
| SAS | -0.17 | 0.2956 | -0.7495 | 0.4094 | 0.5652 |  |  |
| SCID | -0.8625 | 0.4432 | -1.7311 | 0.0061 | 0.0516 |  |  |
| SCL-25 | -0.8235 | 0.8949 | -2.5775 | 0.9305 | 0.3575 |  |  |
| SCL-4a | -0.5412 | 0.8882 | -2.282 | 1.1996 | 0.5423 |  |  |
| Self-reported anxiety | -1.0351 | 0.4239 | -1.8659 | -0.2043 | 0.0146 |  |  |
| SRAS | -0.4578 | 0.3728 | -1.1885 | 0.2729 | 0.2195 |  |  |
| STAI | -0.0177 | 0.224 | -0.4568 | 0.4214 | 0.9372 |  |  |
| STAI/EPDS-3ANXIETY | 0.1163 | 0.6421 | -1.1422 | 1.3749 | 0.8562 |  |  |
| STAI-S | 0.0987 | 0.3521 | -0.5914 | 0.7887 | 0.7793 |  |  |
| STAI-T | 0.9308 | 0.6437 | -0.3308 | 2.1925 | 0.1482 |  |  |
| **Measurement tool for depression** |  |  |  |  |  | 11.78% | 0.0053 |
| HADS | Ref |  |  |  |  |  |  |
| AKUADS | -0.274 | 0.4888 | -1.2321 | 0.6841 | 0.5752 |  |  |
| AUDADIS-IV | -0.7008 | 0.9114 | -2.4871 | 1.0854 | 0.4419 |  |  |
| BDI | 0.2932 | 0.9228 | -1.5155 | 2.1019 | 0.7507 |  |  |
| BSI | -1.9857 | 0.9119 | -3.773 | -0.1984 | 0.0294 |  |  |
| CESD | 0.3005 | 0.3201 | -0.3269 | 0.9279 | 0.3478 |  |  |
| CIDI | -0.3838 | 0.7631 | -1.8795 | 1.1119 | 0.615 |  |  |
| CIS-R | 0.4087 | 0.9083 | -1.3715 | 2.1889 | 0.6527 |  |  |
| Computerized adaptive diagnostic tool CAT-MH® | -2.1063 | 0.9634 | -3.9946 | -0.2181 | 0.0288 |  |  |
| DASS21 | -0.8098 | 0.3119 | -1.4212 | -0.1984 | 0.0094 |  |  |
| DASS42 | 0.7204 | 0.9088 | -1.0608 | 2.5015 | 0.428 |  |  |
| EPDS | -0.0862 | 0.2006 | -0.4793 | 0.3069 | 0.6673 |  |  |
| EPDS PRIME-MD | -0.3379 | 0.9277 | -2.156 | 1.4803 | 0.7157 |  |  |
| EPDS/PHQ-9 | -0.4091 | 0.9127 | -2.1979 | 1.3798 | 0.654 |  |  |
| HDRS | 1.5232 | 0.9067 | -0.254 | 3.3004 | 0.093 |  |  |
| HSCL25 | 0.226 | 0.6606 | -1.0687 | 1.5207 | 0.7323 |  |  |
| MINI | 0.1932 | 0.9089 | -1.5882 | 1.9745 | 0.8317 |  |  |
| PHQ-4 | 1.8061 | 0.9051 | 0.0321 | 3.5801 | 0.046 |  |  |
| PHQ-8 | 0.8852 | 0.9049 | -0.8884 | 2.6589 | 0.328 |  |  |
| PHQ-9 | -0.0279 | 0.3229 | -0.6609 | 0.605 | 0.931 |  |  |
| Previous diagnosis | -0.2542 | 0.9858 | -2.1863 | 1.6779 | 0.7965 |  |  |
| PRIME-MD | 0.7524 | 0.661 | -0.5431 | 2.0479 | 0.255 |  |  |
| PRIME‑MD PHQ | -1.3682 | 0.9485 | -3.2272 | 0.4907 | 0.1491 |  |  |
| Recorded depression | -1.3525 | 0.5412 | -2.4132 | -0.2918 | 0.0124 |  |  |
| SCID | -1.2025 | 0.5048 | -2.1918 | -0.2133 | 0.0172 |  |  |
| SCL-4d | -0.4932 | 0.9048 | -2.2665 | 1.2802 | 0.5857 |  |  |
| SDS | 0.3594 | 0.3802 | -0.3857 | 1.1045 | 0.3445 |  |  |
| Self-reported depression | -0.7322 | 0.4786 | -1.6702 | 0.2057 | 0.126 |  |  |
| SRDS | -0.6723 | 0.4092 | -1.4742 | 0.1297 | 0.1004 |  |  |
| The Tale of Whooley’s two questions | 0.4363 | 0.9109 | -1.3489 | 2.2215 | 0.6319 |  |  |

**References**

1. Sutter-Dallay, A.L., Giaconne-Marcesche, V., Glatigny-Dallay, E., and Verdoux, H.Women with anxiety disorders during pregnancy are at increased risk of intense postnatal depressive symptoms: a prospective survey of the MATQUID cohort. *Eur Psychiatry* 2004；19, 459-463.

2. Adewuya, A.O., and Afolabi, O.T. The course of anxiety and depressive symptoms in Nigerian postpartum women. *Arch Womens Ment Health* 2005；8, 257-259.

3. Andersson, L., Sundström-Poromaa, I., Wulff, M., Aström, M., and Bixo, M. Depression and anxiety during pregnancy and six months postpartum: a follow-up study. *Acta Obstet Gynecol Scand* 2006；85, 937-944.

4. Miller, R.L., Pallant, J.F., and Negri, L.M. Anxiety and stress in the postpartum: Is there more to postnatal distress than depression? *BMC Psychiatry* 2006；6.

5. Lee, A. M., Lam, S. K., Sze Mun Lau, S. M., Chong, C. S., Chui, H. W., & Fong, D. Y. Prevalence, course, and risk factors for antenatal anxiety and depression. *Obstetrics and gynecology* 2007；110(5), 1102–1112.

6. Phillips, J., Sharpe, L., and Matthey, S. Rates of depressive and anxiety disorders in a residential mother-infant unit for unsettled infants. *Aust N Z J Psychiatry* 2007.*41*, 836-842.

7. Sato, Y., Kato, T., & Kakee, N. A six-month follow-up study of maternal anxiety and depressive symptoms among Japanese. *Journal of epidemiology* 2008；18(2), 84–87.

8. Rowe, H.J., Fisher, J.R., and Loh, W.M.. The Edinburgh Postnatal Depression Scale detects but does not distinguish anxiety disorders from depression in mothers of infants. *Arch Womens Ment Health* 2008；11, 103-108.

9. Reck, C., Struben, K., Backenstrass, M., Stefenelli, U., Reinig, K., Fuchs, T., Sohn, C., and Mundt, C. Prevalence, onset and comorbidity of postpartum anxiety and depressive disorders. *Acta Psychiatrica Scandinavica* 2008；118, 459-468.

10. Faisal-Cury, A., Menezes, P., Araya, R., and Zugaib, M. Common mental disorders during pregnancy: prevalence and associated factors among low-income women in São Paulo, Brazil: depression and anxiety during pregnancy. *Arch Womens Ment Health* 2009；12, 335-343.

11. Ali, N.S., Ali, B.S., and Azam, I.S. .Post partum anxiety and depression in peri-urban communities of Karachi, Pakistan: a quasi-experimental study. *BMC Public Health* 2009；9.

12. Giakoumaki, O., Vasilaki, K., Lili, L., Skouroliakou, M., and Liosis, G. The role of maternal anxiety in the early postpartum period: screening for anxiety and depressive symptomatology in Greece. *J Psychosom Obstet Gynaecol* 2009；30, 21-28.

13. Qiao, Y.X., Wang, J., Li, J., and Ablat, A. The prevalence and related risk factors of anxiety and depression symptoms among Chinese pregnant women in Shanghai. *Aust N Z J Obstet Gynaecol* 2009；49, 185-190.

14. Teixeira, C., Figueiredo, B., Conde, A., Pacheco, A., and Costa, R. Anxiety and depression during pregnancy in women and men. *J Affect Disord* 2009；119, 142-148.

15. van Batenburg-Eddes, T., de Groot, L., Huizink, A.C., Steegers, E.A.P., Hofman, A., Jaddoe, V.W.V., Verhulst, F.C., and Tiemeier, H. Maternal Symptoms of Anxiety During Pregnancy Affect Infant Neuromotor Development: The Generation R Study. *Developmental Neuropsychology* 2009；34, 476-493.

16. Woolhouse, H., Brown, S., Krastev, A., Perlen, S., and Gunn, J. Seeking help for anxiety and depression after childbirth: results of the Maternal Health Study. *Archives of Women's Mental Health* 2990；12, 75-83.

17. Austin, M.P., Hadzi-Pavlovic, D., Priest, S.R., Reilly, N., Wilhelm, K., Saint, K., and Parker, G. Depressive and anxiety disorders in the postpartum period: how prevalent are they and can we improve their detection? *Arch Womens Ment Health* 2010；13, 395-401.

18. Goodman, J. H., & Tyer-Viola, L.Detection, treatment, and referral of perinatal depression and anxiety by obstetrical providers. *Journal of women's health* 2010, 19(3), 477–490.

19. Yelland, J., Sutherland, G., and Brown, S.J. Postpartum anxiety, depression and social health: findings from a population-based survey of Australian women. *BMC Public Health* 2010；10.

20. Fisher, J., Tran, T., La, B.T., Kriitmaa, K., Rosenthal, D., and Tran, T.Common perinatal mental disorders in northern Viet Nam: community prevalence and health care use. *Bull World Health Organ* 2010；88, 737-745.

21. Field, T., Diego, M., Hernandez-Reif, M., Figueiredo, B., Deeds, O., Ascencio, A., Schanberg, S., and Kuhn, C.Comorbid depression and anxiety effects on pregnancy and neonatal outcome. *Infant Behavior and Development* 2010；*33*, 23-29.

22. Edhborg, M., Nasreen, H.E., and Kabir, Z.N.Impact of postpartum depressive and anxiety symptoms on mothers' emotional tie to their infants 2-3 months postpartum: a population-based study from rural Bangladesh. *Arch Womens Ment Health* 2011；14, 307-316.

23. Figueiredo, B., and Conde, A.Anxiety and depression in women and men from early pregnancy to 3-months postpartum. *Arch Womens Ment Health* 2011；14, 247-255.

24. Le Strat, Y., Dubertret, C., and Le Foll, B. Prevalence and correlates of major depressive episode in pregnant and postpartum women in the United States. *J Affect Disord* 2011；135, 128-138.

25. Paul, I.M., Downs, D.S., Schaefer, E.W., Beiler, J.S., and Weisman, C.S. Postpartum anxiety and maternal-infant health outcomes. *Pediatrics* 2013；131, e1218-1224.

26. Tavares, D., Quevedo, L., Jansen, K., Souza, L., Pinheiro, R., and Silva, R.. Prevalence of suicide risk and comorbidities in postpartum women in Pelotas. *Braz J Psychiatry* 2012；34, 270-276.

27. Grant, K.A., Bautovich, A., McMahon, C., Reilly, N., Leader, L., and Austin, M.P. Parental care and control during childhood: associations with maternal perinatal mood disturbance and parenting stress. *Arch Womens Ment Health* 2012；15, 297-305.

28. Ali, N.S., Azam, I.S., Ali, B.S., Tabbusum, G., and Moin, S.S.Frequency and associated factors for anxiety and depression in pregnant women: a hospital-based cross-sectional study. *Scientific World Journal 2012*, 653098.

29. Storksen, H.T., Eberhard-Gran, M., Garthus-Niegel, S., and Eskild, A.Fear of childbirth; the relation to anxiety and depression. *Acta Obstet Gynecol Scand* 2012；91, 237-242.

30. Bener, A., Sheikh, and Gerber. Prevalence of psychiatric disorders and associated risk factors in women during their postpartum period: a major public health problem and global comparison. *International Journal of Women's Health* 2012.

31. Ban, L., Gibson, J.E., West, J., Fiaschi, L., Oates, M.R., and Tata, L.J.Impact of socioeconomic deprivation on maternal perinatal mental illnesses presenting to UK general practice. *British Journal of General Practice* 2012;62, e671-e678.

32. Ibanez, G., Charles, M.-A., Forhan, A., Magnin, G., Thiebaugeorges, O., Kaminski, M., and Saurel-Cubizolles, M.-J.Depression and anxiety in women during pregnancy and neonatal outcome: Data from the EDEN mother–child cohort. *Early Human Development* 2012;88, 643-649.

33. Bindt, C., Guo, N., Bonle, M.T., Appiah-Poku, J., Hinz, R., Barthel, D., Schoppen, S., Feldt, T., Barkmann, C., Koffi, M., et al.No association between antenatal common mental disorders in low-obstetric risk women and adverse birth outcomes in their offspring: results from the CDS study in Ghana and Côte D'Ivoire. *PLoS One* 2013;8, e80711.

34. Wynter, K., Rowe, H., and Fisher, J.. Common mental disorders in women and men in the first six months after the birth of their first infant: A community study in Victoria, Australia. *Journal of Affective Disorders* 2013; *151*, 980-985.

35. Makara-Studzińska, M., Morylowska-Topolska, J., Sygit, K., Sygit, M., and Goździewska, M. Socio-demographical and psychosocial determinants of anxiety symptoms in a population of pregnant women in the regions of central and eastern Poland. *Ann Agric Environ Med* 2013;20, 195-202.

36. Thiagayson, P., Krishnaswamy, G., Lim, M.L., Sung, S.C., Haley, C.L., Fung, D.S., Allen, J.C., Jr., and Chen, H.Depression and anxiety in Singaporean high-risk pregnancies - prevalence and screening. *Gen Hosp Psychiatry* 2013;35, 112-116.

37. Bjørk, M.H., Veiby, G., Reiter, S.C., Berle, J., Daltveit, A.K., Spigset, O., Engelsen, B.A., and Gilhus, N.E. Depression and anxiety in women with epilepsy during pregnancy and after delivery: a prospective population-based cohort study on frequency, risk factors, medication, and prognosis. *Epilepsia* 2015;56, 28-39.

38. Enatescu, V.R., Enatescu, I., Craina, M., Gluhovschi, A., Papava, I., Romosan, R., Marian, C., Oprea, A., and Bernad, E.State and trait anxiety as a psychopathological phenomenon correlated with postpartum depression in a Romanian sample: a pilot study. *J Psychosom Obstet Gynaeco*l 2014;35, 55-61.

39. Farr, S.L., Dietz, P.M., O'Hara, M.W., Burley, K., and Ko, J.Y. (2014). Postpartum anxiety and comorbid depression in a population-based sample of women. *J Womens Health (Larchmt)* 23, 120-128.

40. Miller, E.S., Hoxha, D., Wisner, K.L., and Gossett, D.R.The impact of perinatal depression on the evolution of anxiety and obsessive-compulsive symptoms. *Arch Womens Ment Health* 2015;18, 457-461.

41. Koutra, K., Vassilaki, M., Georgiou, V., Koutis, A., Bitsios, P., Chatzi, L., and Kogevinas, M.. Antenatal maternal mental health as determinant of postpartum depression in a population based mother-child cohort (Rhea Study) in Crete, Greece. *Soc Psychiatry Psychiatr Epidemiol* 2014; 49, 711-721.

42. Segre, L.S., McCabe, J.E., Chuffo-Siewert, R., and O’Hara, M.W.Depression and Anxiety Symptoms in Mothers of Newborns Hospitalized on the Neonatal Intensive Care Unit. *Nursing Research* 2014;63, 320-332.

43. Simpson, W., Glazer, M., Michalski, N., Steiner, M., & Frey, B. N.Comparative efficacy of the generalized anxiety disorder 7-item scale and the Edinburgh Postnatal Depression Scale as screening tools for generalized anxiety disorder in pregnancy and the postpartum period. Canadian journal of psychiatry. *Revue canadienne de psychiatrie* 2014;59(8), 434–440.

44. Tendais, I., Costa, R., Conde, A., and Figueiredo, B.. Screening for depression and anxiety disorders from pregnancy to postpartum with the EPDS and STAI. *Span J Psychol* 2014;*17*, E7.

45. Nguyen, T.T., Tran, T.D., Tran, T., La, B., Nguyen, H., and Fisher, J.Postpartum change in common mental disorders among rural Vietnamese women: incidence, recovery and risk and protective factors. *Br J Psychiatry*2015; 206, 110-115.

46. Cheng, T.S., Chen, H., Lee, T., Teoh, O.H., Shek, L.P., Lee, B.W., Chee, C., Godfrey, K.M., Gluckman, P.D., Kwek, K., et al.An independent association of prenatal depression with wheezing and anxiety with rhinitis in infancy. *Pediatr Allergy Immunol* 2015;26, 765-771.

47. Martini, J., Petzoldt, J., Einsle, F., Beesdo-Baum, K., Höfler, M., and Wittchen, H.U. Risk factors and course patterns of anxiety and depressive disorders during pregnancy and after delivery: a prospective-longitudinal study. *J Affect Disord* 2015;175, 385-395.

48. Abdelhai, R., and Mosleh, H. Screening for antepartum anxiety and depression and their association with domestic violence among Egyptian pregnant women. *J Egypt Public Health Assoc* 2015;90, 101-108.

49. Waldie, K.E., Peterson, E.R., D'Souza, S., Underwood, L., Pryor, J.E., Carr, P.A., Grant, C., and Morton, S.M.Depression symptoms during pregnancy: Evidence from Growing Up in New Zealand. *J Affect Disord* 2015;186, 66-73.

50. Falah-Hassani, K., Shiri, R., and Dennis, C.-L.Prevalence and risk factors for comorbid postpartum depressive symptomatology and anxiety. *Journal of Affective Disorders* 2016;198, 142-147.

51. Tham, E.K., Tan, J., Chong, Y.S., Kwek, K., Saw, S.M., Teoh, O.H., Goh, D.Y., Meaney, M.J., and Broekman, B.F. Associations between poor subjective prenatal sleep quality and postnatal depression and anxiety symptoms. *J Affect Disord* 2016;*202*, 91-94.

52. Venkatesh, K.K., Nadel, H., Blewett, D., Freeman, M.P., Kaimal, A.J., and Riley, L.E. Implementation of universal screening for depression during pregnancy: feasibility and impact on obstetric care. *American Journal of Obstetrics and Gynecology* 2016;*215*, 517.e511-517.e518.

53. Chen, J., Cai, Y., Liu, Y., Qian, J., Ling, Q., Zhang, W., Luo, J., Chen, Y., & Shi, S.Factors Associated with Significant Anxiety and Depressive Symptoms in Pregnant Women with a History of Complications. *Shanghai archives of psychiatry* 2016;28(5), 253–262.

54. Dikmen-Yildiz, P., Ayers, S., and Phillips, L.Depression, anxiety, PTSD and comorbidity in perinatal women in Turkey: A longitudinal population-based study. *Midwifery* 2017;55, 29-37.

55. Nasreen, H.E., Rahman, J.A., Rus, R.M., Kartiwi, M., Sutan, R., and Edhborg, M. Prevalence and determinants of antepartum depressive and anxiety symptoms in expectant mothers and fathers: results from a perinatal psychiatric morbidity cohort study in the east and west coasts of Malaysia. *BMC Psychiatry* 2018;18, 195.

56. Odinka, J.I., Nwoke, M., Chukwuorji, J.C., Egbuagu, K., Mefoh, P., Odinka, P.C., Amadi, K.U., and Muomah, R.C.Post-partum depression, anxiety and marital satisfaction: A perspective from Southeastern Nigeria. *S Afr J Psychiatr* 2018;24, 1109.

57. Aryal, K. K., Alvik, A., Thapa, N., Mehata, S., Roka, T., Thapa, P., Pandey, P., & Pedersen, B. S.Anxiety and Depression among Pregnant Women and Mothers of Children Under one Year in Sindupalchowk District. *Journal of Nepal Health Research Council2018;*16(2), 195–204.

58. Dennis, C.L., Brown, H.K., Wanigaratne, S., Fung, K., Vigod, S.N., Grigoriadis, S., Marini, F., and Brennenstuhl, S.Prevalence, Incidence, and Persistence of Postpartum Depression, Anxiety, and Comorbidity among Chinese Immigrant and Nonimmigrant Women: A Longitudinal Cohort Study. *Can J Psychiatry* 2018;63, 44-53.

59. Dennis, C.-L., Brown, H.K., Wanigaratne, S., Vigod, S.N., Grigoriadis, S., Fung, K., Marini, F., and Brennenstuhl, S.Determinants of comorbid depression and anxiety postnatally: A longitudinal cohort study of Chinese-Canadian women. *Journal of Affective Disorders* 2018;227, 24-30.

60. Redinger, S., Norris, S.A., Pearson, R.M., Richter, L., and Rochat, T. First trimester antenatal depression and anxiety: prevalence and associated factors in an urban population in Soweto, South Africa. *Journal of Developmental Origins of Health and Disease* 2017*;9,* 30-40.

61. Suzuki, S., Yamada, F., and Eto, M. (2018). Influence of intimate partner violence on mental status in Japanese women during the first trimester of pregnancy. J Matern Fetal Neonatal Med *31*, 1524-1526.

62. Nakić Radoš, S.Anxiety During Pregnancy and Postpartum: Course, Predictors and Comorbidity with Postpartum Depression. *Acta Clinica Croatica* 2018;57, 39-51.

63. González-Hernández, A., González-Hernandez, D., Fortuny-Falconi, C.M., Tovilla-Zárate, C.A., Fresan, A., Nolasco-Rosales, G.A., Juárez-Rojop, I.E., López-Narváez, M.L., Gonzalez-Castro, T.B., and Escobar Chan, Y.M.Prevalence and Associated Factors to Depression and Anxiety in Women with Premature Babies Hospitalized in a Neonatal Intensive-Care Unit in a Mexican Population. *Journal of Pediatric Nursing* 2019;45, e53-e56.

64. Agbaje, O.S., Anyanwu, J.I., Umoke, P.I.C., Iwuagwu, T.E., Iweama, C.N., Ozoemena, E.L., and Nnaji, I.R.Depressive and anxiety symptoms and associated factors among postnatal women in Enugu-North Senatorial District, South-East Nigeria: a cross-sectional study. *Archives of Public Health* 2019;77.

65. Leff Gelman, P., Mancilla-Herrera, I., Flores-Ramos, M., Saravia Takashima, M.F., Cruz Coronel, F.M., Cruz Fuentes, C., Perez Molina, A., Hernandez-Ruiz, J., Silva-Aguilera, F.S., Farfan-Labonne, B., et al.The cytokine profile of women with severe anxiety and depression during pregnancy. *BMC Psychiatry* 2019;19, 104.

66. Aris-Meijer, J., Bockting, C., Stolk, R., Verbeek, T., Beijers, C., van Pampus, M., and Burger, H.What If Pregnancy Is Not Seventh Heaven? The Influence of Specific Life Events during Pregnancy and Delivery on the Transition of Antenatal into Postpartum Anxiety and Depression. *Int J Environ Res Public Health* 2019;16.

67. Ngocho, J.S., Watt, M.H., Minja, L., Knettel, B.A., Mmbaga, B.T., Williams, P.P., and Sorsdahl, K. Depression and anxiety among pregnant women living with HIV in Kilimanjaro region, Tanzania. *PLoS One* 2019;14, e0224515.

68. Tay, C.T., Teede, H.J., Boyle, J.A., Kulkarni, J., Loxton, D., and Joham, A.E. Perinatal Mental Health in Women with Polycystic Ovary Syndrome: A Cross-Sectional Analysis of an Australian Population-Based Cohort. *J Clin Med* 2019;8.

69. Uguz, F., Yakut, E., Aydogan, S., Bayman, M.G., and Gezginc, K.The impact of maternal major depression, anxiety disorders and their comorbidities on gestational age, birth weight, preterm birth and low birth weight in newborns. *J Affect Disord* 2019;259, 382-385.

70. Genovez, M.Postpartum Depression and Anxiety in Central American Immigrant Women: The Role of Familism and Social Suppor.2019.

71. Ramakrishna, S., Cooklin, A.R., and Leach, L.S.Comorbid anxiety and depression: a community-based study examining symptomology and correlates during the postpartum period. *Journal of Reproductive and Infant Psychology* 2019;37, 468-479.

72. Wassif, O. M., Abdo, A. S., Elawady, M. A., Abd Elmaksoud, A. E., & Eldesouky, R. S. Assessment of Postpartum Depression and Anxiety among Females Attending Primary Health Care Facilities in Qaliubeya Governorate, Egypt. *Journal of environmental and public health*;2019, 3691752.

73. Redinger, S., Pearson, R.M., Houle, B., Norris, S.A., and Rochat, T.J.Antenatal depression and anxiety across pregnancy in urban South Africa. *Journal of Affective Disorders* 2020;277, 296-305.

74. Umuziga, M.P., Adejumo, O., and Hynie, M.A cross-sectional study of the prevalence and factors associated with symptoms of perinatal depression and anxiety in Rwanda. *BMC Pregnancy and Childbirth* 2020;20.

75. Gonzalez-Mesa, E., Kabukcuoglu, K., Blasco, M., Korukcu, O., Ibrahim, N., Gonzalez-Cazorla, A., and Cazorla, O.Comorbid anxiety and depression (CAD) at early stages of the pregnancy. A multicultural cross-sectional study. *J Affect Disord* 2020;270, 85-89.

76. Premji, S.S., Lalani, S., Shaikh, K., Mian, A., Forcheh, N., Dosani, A., Letourneau, N., Yim, I.S., Bhamani, S.S., and Maternal-Infant Global Health Team-Collaborators In Research, M. Comorbid Anxiety and Depression among Pregnant Pakistani Women: Higher Rates, Different Vulnerability Characteristics, and the Role of Perceived Stress. *Int J Environ Res Public Health* 2020;17.

77. Jyothi Kantipudi, S., Kannan, G.K., Viswanathan, S., Ranganathan, S., Menon, J., and Ramanathan, S.Antenatal Depression and Generalized Anxiety Disorder in a Tertiary Hospital in South India. *Indian Journal of Psychological Medicine* 2020;42, 513-518.

78. Pabon, S., Parpinelli, M.A., Narvaez, M.B., Charles, C.M.p., Guida, J.P., Escobar, M.F., Cecatti, J.G., and Costa, M.L.Overall Maternal Morbidity during Pregnancy Identified with the WHO-WOICE Instrument. *BioMed Research International* *2020*, 9740232.

79. Coo, S., García, M.I., and Mira, A.Examining the association between subjective childbirth experience and maternal mental health at six months postpartum. *J Reprod Infant Psychol* 2023;41, 275-288.

80. Dong, H., Hu, R., Lu, C., Huang, D., Cui, D., Huang, G., and Zhang, M.Investigation on the mental health status of pregnant women in China during the Pandemic of COVID-19. *Arch Gynecol Obstet* 2021;303, 463-469.

81. Bante, A., Mersha, A., Zerdo, Z., Wassihun, B., and Yeheyis, T.Comorbid anxiety and depression: Prevalence and associated factors among pregnant women in Arba Minch zuria district, Gamo zone, southern Ethiopia. *PLoS One* 2021;16, e0248331.

82. Jha, S., Salve, H.R., Goswami, K., Sagar, R., and Kant, S.Prevalence of Common Mental Disorders among pregnant women-Evidence from population-based study in rural Haryana, India. *J Family Med Prim Care* 2021;10, 2319-2324.

83. Wensu, Z., Xidi, Z., Shaojie, L., Baohua, Z., Yunhan, Y., Huilan, X., Zhao, H., and Xiyue, X. Does the Presence of Anxiety and Depression Symptoms Mediate the Association Between Family Functions and Self-Efficacy in Pregnant Women in the Third Trimester?: A Community-Based Cross-Sectional Survey. *Front Psychiatry* 2021;12,726093.

84. Arshad, A., Foresti, K., Rech, M., Brakoulias, V., and Zubaran, C. Demoralization, depression and anxiety in postpartum women of culturally and linguistic diverse backgrounds in Australia. *European Journal of Midwifery* 2021;5, 45.

85. Cena, L., Gigantesco, A., Mirabella, F., Palumbo, G., Camoni, L., Trainini, A., and Stefana, A. Prevalence of comorbid anxiety and depressive symptomatology in the third trimester of pregnancy: Analysing its association with sociodemographic, obstetric, and mental health features. *Journal of Affective Disorders* 2021;295, 1398-1406.

86. Cheng, C.-Y., Chou, Y.-H., Chang, C.-H., and Liou, S.-R.Trends of Perinatal Stress, Anxiety, and Depression and Their Prediction on Postpartum Depression. *International Journal of Environmental Research and Public Health* 2021;18.

87. Lilliecreutz, C., Josefsson, A., Mohammed, H., Josefsson, A., and Sydsjö, G.Mental disorders and risk factors among pregnant women with depressive symptoms in Sweden—A case‐control study. *Acta Obstetricia et Gynecologica Scandinavica* 2021;100, 1068-1074.

88. Luo, Z., Xue, L., Ma, L., and Liu, Z.Comorbid Anxiety and Depression and Related Factors Among Pregnant and Postpartum Chinese Women During the Coronavirus Disease 2019 Pandemic. *Frontiers in Psychology* 2021;12.

89. Nasreen, H.E., Pasi, H.B., Aris, M.A.M., Rahman, J.A., Rus, R.M., and Edhborg, M.Impact of parental perinatal depressive and anxiety symptoms trajectories on early parent-infant impaired bonding: a cohort study in east and west coasts of Malaysia. *Archives of Women's Mental Health* 2021;25, 377-387.

90. Stampini, V., Monzani, A., Caristia, S., Ferrante, G., Gerbino, M., De Pedrini, A., Amadori, R., Rabbone, I., and Surico, D.The perception of Italian pregnant women and new mothers about their psychological wellbeing, lifestyle, delivery, and neonatal management experience during the COVID-19 pandemic lockdown: a web-based survey. *BMC Pregnancy and Childbirth* 2021;21.

91. Mateus, V., Cruz, S., Costa, R., Mesquita, A., Christoforou, A., Wilson, C.A., Vousoura, E., Dikmen-Yildiz, P., Bina, R., Dominguez-Salas, S., et al.Rates of depressive and anxiety symptoms in the perinatal period during the COVID-19 pandemic: Comparisons between countries and with pre-pandemic data. *J Affect Disord* 2021;316, 245-253.

92. Zhou, Y., Huang, J., Baker, P.N., Liao, B., and Yu, X.The prevalence and associated factors of prenatal depression and anxiety in twin pregnancy: a cross-sectional study in Chongqing, China. *BMC Pregnancy Childbirth* 2022;22, 877.

93. Ray, P.B., Chakraborty, M.K., & Hazra, S.A Cross-sectional Study to Assess the Anxiety and Depression among Perinatal Mothers during the COVID-19 Pandemic. *Journal of South Asian Federation of Obstetrics and Gynaecology*.2022.

94. Abdus-Salam, R.A., Balogun, R.O., Lawal, T.V., Lawal, O.O., Lawal, R.O., Abdulmalik, J.O., and Morhason-Bello, I.O.Assessment of anxiety and depression, and coping mechanisms during COVID-19 lockdown among pregnant women. *Heliyon* 2022;8, e10902.

95. Motrico, E., Dominguez-Salas, S., Rodriguez-Dominguez, C., Gomez-Gomez, I., Rodriguez-Munoz, M.F., and Gomez-Baya, D.The Impact of the COVID-19 Pandemic on Perinatal Depression and Anxiety: A Large Cross-sectional Study in Spain.*Psicothema* 2022;34, 200-208.

96. Avalos, L.A., Nance, N., Badon, S.E., Young-Wolff, K., Ames, J., Zhu, Y., Hedderson, M.M., Ferrara, A., Zerbo, O., Greenberg, M., and Croen, L.A.Associations of COVID-19-Related Health, Healthcare and Economic Factors With Prenatal Depression and Anxiety. *International Journal of Public Health* 2022;67.

97. Esquivel Lauzurique, M., Vera Fernández, Y., Dennis, C.-L., Rubén Quesada, M., Álvarez Valdés, G., Lye, S., and Tamayo-Pérez, V.Prevalence, Incidence, and Persistence of Postpartum Anxiety, Depression, and Comorbidity. *Journal of Perinatal & Neonatal Nursing* 2022;36, E15-E24.

98. Irum, S., Khan, A.A., Rabbani, U., Lodhi, F.S., and Elsous, A.Frequency and Risk Factors of Anxiety and Depression among Pregnant Women in Abbottabad, Pakistan: A Facility-Based Cross-Sectional Study. *Pakistan Journal of Medical and Health Sciences* 2022;16, 465-468.

99. Ma, S., Yin, X., Tao, R., Jiang, X., Xie, J., Li, P., Zhu, D., and Zhu, P. Association of maternal prenatal depression and anxiety with toddler sleep: the China-Anhui Birth Cohort study. *Archives of Women's Mental Health* 2022;25, 431-439.

100. Malaju, M.T., Alene, G.D., and Bisetegn, T.A.Longitudinal path analysis for the directional association of depression, anxiety and posttraumatic stress disorder with their comorbidities and associated factors among postpartum women in Northwest Ethiopia: A cross-lagged autoregressive modelling study. *PLoS One* 2022;17, e0273176.

101. Odinka, J.I., Nwoke, M., Chukwuorji, J.C., Mefoh, P., Ezeme, M.S., Nduanya, U.C., Oraegbunam, C., and Odinka, P.C.Mindful awareness as a predictor of postpartum depression and anxiety among Igbo nursing mothers in Enugu, South-East Nigeria. *Mental Health, Religion & Culture* 2022;25, 578-591.

102. Yang, H., Pan, Y., Chen, W., Yang, X., Liu, B., Yuan, N., and Zhang, X.Prevalence of and relevant factors for depression and anxiety symptoms among pregnant women on the eastern seaboard of China in the post-COVID-19 era: a cross-sectional study. *BMC Psychiatry* 2023;23(1), 564.

103. Hannon, S., Gartland, D., Higgins, A., Brown, S.J., Carroll, M., Begley, C., and Daly, D. Physical health and comorbid anxiety and depression across the first year postpartum in Ireland (MAMMI study): A longitudinal population-based study. *J Affect Disord* 2023;328, 228-237.

104. Yang, Z., Wang, X., Wang, M., Yan, S., Wu, F., and Zhang, F.Trajectory of prenatal anxiety and depression and its association with fetal growth development. *Early Hum Dev* 2023;187, 105875.

105. Craemer, K.A., Garland, C.E., Sayah, L., Duffecy, J., Geller, S.E., and Maki, P.M.Perinatal mental health in low-income urban and rural patients: The importance of screening for comorbidities. *General Hospital Psychiatry* 2023;83, 130-139.

106. Hou, Y., Shang, M., Yu, X., Gu, Y., Li, H., Lu, M., Jiang, M., Zhen, H., Zhu, B., and Tao, F. Joint effects of recent stressful life events and adverse childhood experiences on perinatal comorbid anxiety and depression. *BMC Pregnancy and Childbirth* 2023;23(1), 41.

107. Tambawala, Z.Y., Saquib, S., Salman, A., Khalid, M., Beshtawi, H., Akbari, E.A., Binashoor, A., Hubaishi, N., Hamza, L.K., and Rayssi, M.A.Perinatal anxiety and depression amidst the COVID-19 pandemic in Dubai, United Arab Emirates*. AJOG Global Reports* 2023;3(1), 100164.

108. Bertram, G., Becuzzi, N., & Wilson, E. Trends in perinatal mental health within a tertiary institution from 2013 to 2022. *The Australian & New Zealand journal of obstetrics & gynaecology* 2023; 63(6), 821–824.

109. Dadhwal, V., Sagar, R., Bhattacharya, D., Kant, S., Misra, P., Choudhary, V., & Vanamail, P. Prevalence of postpartum depression & anxiety among women in rural India: Risk factors & psychosocial correlates. *The Indian journal of medical research* 2023; 158(4), 407–416.

110. Gennaro, S., Melnyk, B. M., Szalacha, L. A., Hoying, J., Cooper, A., Aviles, M. M., O'Connor, C., & Gibeau, A. Depression, anxiety, and stress in pregnant Black people: A case for screening and evidence-based intervention. *The Nurse practitioner* 2023; 48(12), 37–46.

111. Costa, R., Mesquita, A., Motrico, E., Domínguez-Salas, S., Dikmen-Yildiz, P., Saldivia, S., Vousoura, E., Osorio, A., Wilson, C. A., Bina, R., Levy, D., Christoforou, A., González, M. F., Hancheva, C., Felice, E., & Pinto, T. M. Unmet needs in mental healthcare for women with clinically significant symptoms of perinatal depression and/or anxiety during the COVID-19 pandemic. *Acta psychiatrica Scandinavica* 2024; 150(5), 474–491.

112. Fijean, A. L., Marçais, M., Banasiak, C., Morel, O., Dahlhoff, S., Olieric, M. F., Mottet, N., Epstein, J., & Bertholdt, C. Universal screening of postpartum depression with Edinburgh Postpartum Depression Scale: A prospective observational study. *International journal of gynaecology and obstetrics: the official organ of the International Federation of Gynaecology and Obstetrics* 2024; 167(2), 758–764.

113. Altendahl, M. R., Xu, L., Asiodu, I., Boscardin, J., Gaw, S. L., Flaherman, V. J., Jacoby, V. L., Richards, M. C., Krakow, D., & Afshar, Y. Patterns of Peripartum Depression and Anxiety During the Pre-Vaccine COVID-19 Pandemic. *Research square* 2022; rs.3.rs-2294673.

114. Eriksson, A., Kimmel, M. C., Furmark, T., Wikman, A., Grueschow, M., Skalkidou, A., Frick, A., & Fransson, E. Investigating heart rate variability measures during pregnancy as predictors of postpartum depression and anxiety: an exploratory study. *Translational psychiatry* 2024; 14(1), 203.

115. Bain, M., Park, S., Zaidi, A., Atif, N., Rahman, A., Malik, A., & Surkan, P. J. Social Support and Spousal Relationship Quality Improves Responsiveness among Anxious Mothers. *Child psychiatry and human development* 2024; 10.1007/s10578-024-01702-5. Advance online publication.

116. Bante, A., Ayele, G., Alamirew, B., & Ahmed, M. Regulatory problems and associated factors among infants in Arba Minch health and demographic surveillance system sites, southern Ethiopia. *PloS one* 2024; 19(6), e0305722.

117. Chen, J. J., Chen, X. J., She, Q. M., Li, J. X., & Luo, Q. H. Clinical risk factors for preterm birth and evaluating maternal psychology in the postpartum period. *World journal of psychiatry* 2024; 14(5), 661–669.

118. Haile, T. T., Kebede, A. A., Gessesse, D. N., Tsega, N. T., Aklil, M. B., Temesgan, W. Z., Anteneh, T. A., Tibebu, N. S., Alemu, H. N., Seyoum, A. T., Tiguh, A. E., Yismaw, A. E., Mihret, M. S., Nenko, G., Wondie, K. Y., Taye, B. T., & Abegaz, M. Y. Anxiety and associated factors in Northwest Ethiopian pregnant women: a broad public health concern.*Frontiers in public health* 2024; 11, 1300229.

119. Shen, Q, Xiao, M, Wang, B, He, T, Zhao, J, & Lei, J. Comorbid Anxiety and Depression among Pregnant and Postpartum Women: A Longitudinal Population-Based Study, *Depression and Anxiety* 2024; 7802142, 9 pages.

120. Zheng, J, Huang, L, Zhang, Y, Shen, M, Wang, X, Li, H, Liu, Z, Tian, Y, Lei, X, & Gao, Y. Associations between lifestyle and comorbid anxiety and depression in pregnant women[J]. *Journal of Environmental and Occupational Medicine* 2024; 41(3): 235-242.

121. Jin, X., Zhu, J., Wang, N., Sun, L., Yu, J., Wang, S., & Sun, G. Eating behavior during pregnancy mediates the association between depression and diet quality--a new strategy for intervention in pregnancy. *Frontiers in public health* 2024; 12, 1339149.

122. Ma, J., He, W., Fan, C., Duan, J., Wu, J., Zhang, C., & Peng, Y. The Adverse Effects of Anxiety and Depression on Pregnant Women with Hypothyroidism in Late Pregnancy. *Actas espanolas de psiquiatria* 2024; 52(5), 625–631.
