# Supplementary figures and images for: Prevalence of co-morbid anxiety and depression in pregnancy and postpartum: a systematic review and meta-analysis
Source: Psychol Med. 2025 Mar 13;55:e84. doi: 10.1017/S0033291725000601 (PMC12080659; doi:10.1017/S0033291725000601)

**Funnel Plot**

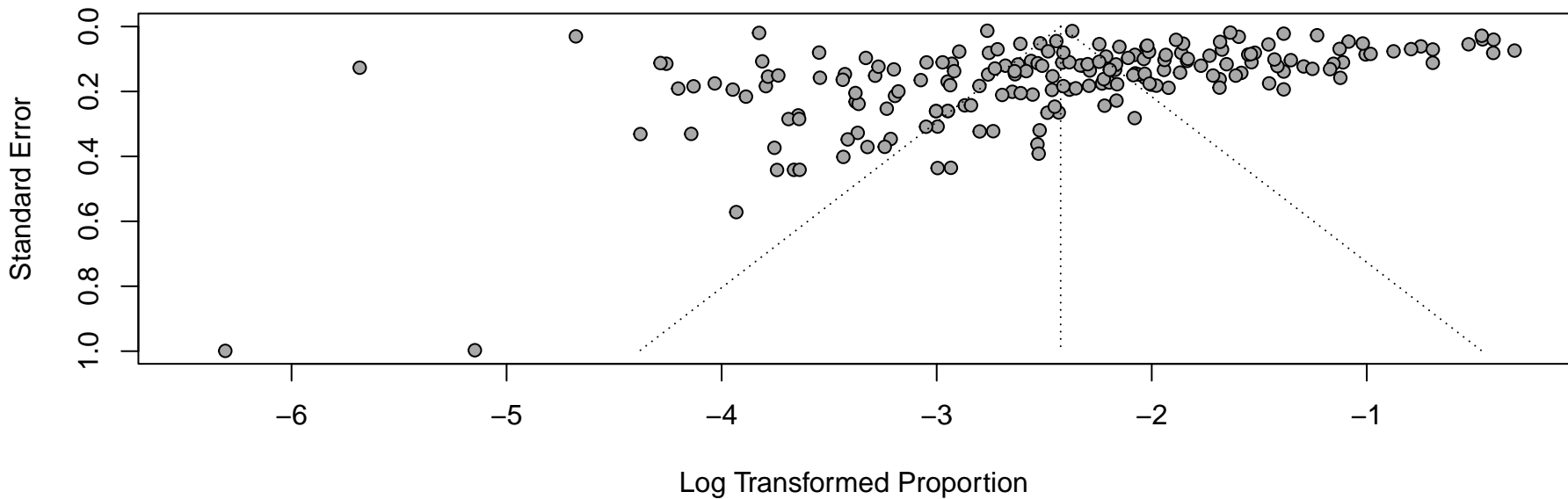

Supplement: Ou et al. supplementary material 5 — Ou et al. supplementary material [file S0033291725000601sup005.pdf]
