# Supplementary material for: Prevalence of co-morbid anxiety and depression in pregnancy and postpartum: a systematic review and meta-analysis
Source: Psychol Med. 2025 Mar 13;55:e84. doi: 10.1017/S0033291725000601 (PMC12080659; doi:10.1017/S0033291725000601)
Supplement: Ou et al. supplementary material 2 — Ou et al. supplementary material [file S0033291725000601sup002.pdf]

| Study | Events | Total | Proportion | 95%-CI | Weight |
|---|---|---|---|---|---|
| **Country_type = High-income** | | | | | |
| Sutter-Dallay 2004 | 13 | 497 | 0.03 | [0.01; 0.04] | 0.5% |
| Andersson 2006 (1) | 39 | 190 | 0.21 | [0.15; 0.27] | 0.5% |
| Andersson 2006 (2) | 25 | 107 | 0.23 | [0.16; 0.33] | 0.5% |
| Miller 2006 | 23 | 325 | 0.07 | [0.05; 0.10] | 0.5% |
| Phillips 2007 | 31 | 167 | 0.19 | [0.13; 0.25] | 0.5% |
| Sato 2008 (1) | 55 | 1348 | 0.04 | [0.03; 0.05] | 0.5% |
| Sato 2008 (2) | 72 | 1348 | 0.05 | [0.04; 0.07] | 0.5% |
| Sato 2008 (3) | 26 | 1348 | 0.02 | [0.01; 0.03] | 0.5% |
| Sato 2008 (4) | 39 | 1348 | 0.03 | [0.02; 0.04] | 0.5% |
| Rowe 2008 | 15 | 138 | 0.11 | [0.06; 0.17] | 0.5% |
| Reck 2008 | 21 | 1024 | 0.02 | [0.01; 0.03] | 0.5% |
| Giakoumakigiako 2009 (1) | 86 | 235 | 0.37 | [0.30; 0.43] | 0.5% |
| Giakoumakigiako 2009 (2) | 48 | 175 | 0.27 | [0.21; 0.35] | 0.5% |
| Giakoumakigiako 2009 (3) | 88 | 235 | 0.37 | [0.31; 0.44] | 0.5% |
| Giakoumakigiako 2009 (4) | 35 | 175 | 0.20 | [0.14; 0.27] | 0.5% |
| Teixeira 2009 (1) | 29 | 270 | 0.11 | [0.07; 0.15] | 0.5% |
| Teixeira 2009 (2) | 21 | 270 | 0.08 | [0.05; 0.12] | 0.5% |
| Teixeira 2009 (3) | 30 | 270 | 0.11 | [0.08; 0.15] | 0.5% |
| Batenburg-Eddes 2009 | 75 | 5296 | 0.01 | [0.01; 0.02] | 0.5% |
| Woolhouse 2009 | 45 | 1385 | 0.03 | [0.02; 0.04] | 0.5% |
| Austin 2010 | 29 | 1289 | 0.02 | [0.02; 0.03] | 0.5% |
| Goodman 2010 (1) | 17 | 491 | 0.03 | [0.02; 0.05] | 0.5% |
| Goodman 2010 (2) | 7 | 299 | 0.02 | [0.01; 0.05] | 0.5% |
| Yelland 2010 | 344 | 4269 | 0.08 | [0.07; 0.09] | 0.5% |
| Field 2010 | 308 | 911 | 0.34 | [0.31; 0.37] | 0.5% |
| Figueiredo 2011 | 24 | 260 | 0.09 | [0.06; 0.13] | 0.5% |
| Le Strat 2011 | 78 | 1524 | 0.05 | [0.04; 0.06] | 0.5% |
| Paul 2012 | 42 | 1123 | 0.04 | [0.03; 0.05] | 0.5% |
| Grant 2012 (1) | 7 | 88 | 0.08 | [0.03; 0.16] | 0.5% |
| Grant 2012 (2) | 11 | 88 | 0.12 | [0.06; 0.21] | 0.5% |
| Storksen 2012 | 78 | 1642 | 0.05 | [0.04; 0.06] | 0.5% |
| Bener 2012 | 63 | 1659 | 0.04 | [0.03; 0.05] | 0.5% |
| Ban 2012 (1) | 1083 | 116457 | 0.01 | [0.01; 0.01] | 0.5% |
| Ban 2012 (2) | 2539 | 116457 | 0.02 | [0.02; 0.02] | 0.5% |
| Ibanez 2012 | 227 | 1719 | 0.13 | [0.12; 0.15] | 0.5% |
| Wynter 2013 | 1 | 172 | 0.01 | [0.00; 0.03] | 0.3% |
| Thiagayson 2013 | 10 | 200 | 0.05 | [0.02; 0.09] | 0.5% |
| Bjørk 2014 | 5510 | 87468 | 0.06 | [0.06; 0.06] | 0.5% |
| Farr 2014 | 327 | 4451 | 0.07 | [0.07; 0.08] | 0.5% |
| Miller 2014 | 28 | 461 | 0.06 | [0.04; 0.09] | 0.5% |
| Koutra 2014 | 50 | 438 | 0.11 | [0.09; 0.15] | 0.5% |
| Segre 2014 | 27 | 200 | 0.14 | [0.09; 0.19] | 0.5% |
| Simpson 2014 | 100 | 240 | 0.42 | [0.35; 0.48] | 0.5% |
| Tendais 2014 (1) | 9 | 139 | 0.06 | [0.03; 0.12] | 0.5% |
| Tendais 2014 (2) | 5 | 94 | 0.05 | [0.02; 0.12] | 0.4% |
| Cheng 2015 | 82 | 1062 | 0.08 | [0.06; 0.09] | 0.5% |
| Martini 2015 | 65 | 302 | 0.22 | [0.17; 0.27] | 0.5% |
| Waldie 2015 | 78 | 5659 | 0.01 | [0.01; 0.02] | 0.5% |
| Falah-Hassani 2016 | 87 | 667 | 0.13 | [0.11; 0.16] | 0.5% |
| Tham 2016 (1) | 60 | 313 | 0.19 | [0.15; 0.24] | 0.5% |
| Tham 2016 (2) | 41 | 313 | 0.13 | [0.10; 0.17] | 0.5% |
| Venkatesh 2016 (1) | 102 | 576 | 0.18 | [0.15; 0.21] | 0.5% |
| Venkatesh 2016 (2) | 24 | 576 | 0.04 | [0.03; 0.06] | 0.5% |
| Dennis 2018 (1) | 81 | 564 | 0.14 | [0.12; 0.18] | 0.5% |
| Dennis 2018 (2) | 49 | 483 | 0.10 | [0.08; 0.13] | 0.5% |
| Dennis 2018 | 120 | 549 | 0.22 | [0.18; 0.26] | 0.5% |
| Suzuki 2018 | 75 | 470 | 0.16 | [0.13; 0.20] | 0.5% |
| Aris-Meijer 2019 | 85 | 3842 | 0.02 | [0.02; 0.03] | 0.5% |
| Tay 2019 (1) | 151 | 5239 | 0.03 | [0.02; 0.03] | 0.5% |
| Tay 2019 (2) | 455 | 5239 | 0.09 | [0.08; 0.09] | 0.5% |
| Genovez 2019 | 5 | 100 | 0.05 | [0.02; 0.11] | 0.4% |
| Ramakrishna 2019 | 143 | 1070 | 0.13 | [0.11; 0.16] | 0.5% |
| González-Mesa 2020 (2) | 25 | 263 | 0.10 | [0.06; 0.14] | 0.5% |
| Kantipudi 2020 | 28 | 209 | 0.13 | [0.09; 0.19] | 0.5% |
| Jha 2021 | 12 | 457 | 0.03 | [0.01; 0.05] | 0.5% |
| Arshad 2021 | 6 | 75 | 0.08 | [0.03; 0.17] | 0.5% |
| Cena 2021 | 64 | 934 | 0.07 | [0.05; 0.09] | 0.5% |
| Lilliecreutz 2021 | 22 | 299 | 0.07 | [0.05; 0.11] | 0.5% |
| Stampini1 2021 | 464 | 739 | 0.63 | [0.59; 0.66] | 0.5% |
| Ray 2022 | 23 | 124 | 0.19 | [0.12; 0.27] | 0.5% |
| Mateus 2022 (1) | 504 | 3326 | 0.15 | [0.14; 0.16] | 0.5% |
| Mateus 2022 (2) | 799 | 3939 | 0.20 | [0.19; 0.22] | 0.5% |
| Motrico 2022 | 980 | 3356 | 0.29 | [0.28; 0.31] | 0.5% |
| Avalos 2022 | 1559 | 6236 | 0.25 | [0.24; 0.26] | 0.5% |
| Hannon 2023 (1) | 27 | 1804 | 0.01 | [0.01; 0.02] | 0.5% |
| Hannon 2023 (2) | 29 | 1804 | 0.02 | [0.01; 0.02] | 0.5% |
| Hannon 2023 (3) | 41 | 1804 | 0.02 | [0.02; 0.03] | 0.5% |
| Hannon 2023 (4) | 32 | 1804 | 0.02 | [0.01; 0.02] | 0.5% |
| Hannon 2023 (5) | 43 | 1804 | 0.02 | [0.02; 0.03] | 0.5% |
| Craemer 2023 | 9 | 717 | 0.01 | [0.01; 0.02] | 0.5% |
| Tambawala 2023 | 142 | 438 | 0.32 | [0.28; 0.37] | 0.5% |
| Bertram 2023 | 4638 | 49598 | 0.09 | [0.09; 0.10] | 0.5% |
| Dadhwal 2023 | 43 | 680 | 0.06 | [0.05; 0.08] | 0.5% |
| Gennaro 2023 | 7 | 194 | 0.04 | [0.01; 0.07] | 0.5% |
| Costa 2024 | 2303 | 11809 | 0.20 | [0.19; 0.20] | 0.5% |
| Fijean 2024 | 109 | 508 | 0.21 | [0.18; 0.25] | 0.5% |
| Altendahl 2024(1) | 15 | 174 | 0.09 | [0.05; 0.14] | 0.5% |
| Altendahl 2024(2) | 27 | 301 | 0.09 | [0.06; 0.13] | 0.5% |
| Altendahl 2024(3) | 21 | 311 | 0.07 | [0.04; 0.10] | 0.5% |
| Altendahl 2024(4) | 28 | 243 | 0.12 | [0.08; 0.16] | 0.5% |
| Eriksson 2024 | 9 | 112 | 0.08 | [0.04; 0.15] | 0.5% |
| **Random effects model** | | **479376** | **0.08** | **[0.06; 0.09]** | **47.2%** |
| Heterogeneity: $I^2 = 99.7\%$, $\tau^2 = 0.8911$, $p = 0$ | | | | | |
| **Country_type = Low-income** | | | | | |
| Adewuya 2005 (1) | 62 | 632 | 0.10 | [0.08; 0.12] | 0.5% |
| Adewuya 2005 (2) | 33 | 630 | 0.05 | [0.04; 0.07] | 0.5% |
| Adewuya 2005 (3) | 43 | 600 | 0.07 | [0.05; 0.10] | 0.5% |
| Adewuya 2005 (4) | 29 | 547 | 0.05 | [0.04; 0.08] | 0.5% |
| Adewuya 2005 (5) | 21 | 512 | 0.04 | [0.03; 0.06] | 0.5% |
| Adewuya 2005 (6) | 12 | 480 | 0.02 | [0.01; 0.04] | 0.5% |
| Ali 2009 (1) | 14 | 267 | 0.05 | [0.03; 0.09] | 0.5% |
| Ali 2009 (2) | 14 | 267 | 0.05 | [0.03; 0.09] | 0.5% |
| Ali 2009 (3) | 27 | 267 | 0.10 | [0.07; 0.14] | 0.5% |
| Ali 2009 (4) | 35 | 267 | 0.13 | [0.09; 0.18] | 0.5% |
| Ali 2012 | 55 | 167 | 0.33 | [0.26; 0.41] | 0.5% |
| Odinka 2018 | 205 | 309 | 0.66 | [0.61; 0.72] | 0.5% |
| Aryal 2018 (1) | 24 | 164 | 0.15 | [0.10; 0.21] | 0.5% |
| Aryal 2018 (2) | 65 | 567 | 0.11 | [0.09; 0.14] | 0.5% |
| Agbaje 2019 | 69 | 267 | 0.26 | [0.21; 0.32] | 0.5% |
| Ngocho 2019 | 36 | 200 | 0.18 | [0.13; 0.24] | 0.5% |
| Umuziga 2020 | 52 | 165 | 0.32 | [0.25; 0.39] | 0.5% |
| Premji 2020 (1) | 24 | 282 | 0.09 | [0.06; 0.12] | 0.5% |
| Premji 2020 (2) | 16 | 282 | 0.06 | [0.03; 0.09] | 0.5% |
| Premji 2020 (3) | 14 | 282 | 0.05 | [0.03; 0.08] | 0.5% |
| Premji 2020 (4) | 14 | 282 | 0.05 | [0.03; 0.08] | 0.5% |
| Bante 2021 | 67 | 667 | 0.10 | [0.08; 0.13] | 0.5% |
| Abdus-salam 2022 | 15 | 380 | 0.04 | [0.02; 0.06] | 0.5% |
| IRUM 2022 | 100 | 200 | 0.50 | [0.43; 0.57] | 0.5% |
| Malaju 2022 (1) | 112 | 775 | 0.14 | [0.12; 0.17] | 0.5% |
| Malaju 2022 (2) | 94 | 775 | 0.12 | [0.10; 0.15] | 0.5% |
| Malaju 2022 (3) | 63 | 775 | 0.08 | [0.06; 0.10] | 0.5% |
| Odinka 2022 | 74 | 309 | 0.24 | [0.19; 0.29] | 0.5% |
| Bain 2024 | 62 | 18218 | 0.00 | [0.00; 0.00] | 0.5% |
| Bante 2024 | 50 | 449 | 0.11 | [0.08; 0.14] | 0.5% |
| Haile 2024 | 56 | 858 | 0.07 | [0.05; 0.08] | 0.5% |
| **Random effects model** | | **30842** | **0.09** | **[0.07; 0.13]** | **16.2%** |
| Heterogeneity: $I^2 = 99\%$, $\tau^2 = 0.9699$, $p = 0$ | | | | | |
| **Country_type = Middle-income** | | | | | |
| Lee 2007 (1) | 48 | 335 | 0.14 | [0.11; 0.19] | 0.5% |
| Lee 2007 (2) | 42 | 335 | 0.13 | [0.09; 0.17] | 0.5% |
| Lee 2007 (3) | 57 | 335 | 0.17 | [0.13; 0.21] | 0.5% |
| Faisal-Cury 2009 | 129 | 831 | 0.16 | [0.13; 0.18] | 0.5% |
| Qiao 2009 | 18 | 527 | 0.03 | [0.02; 0.05] | 0.5% |
| Fisher 2010 | 8 | 199 | 0.04 | [0.02; 0.08] | 0.5% |
| Edhborg 2011 | 23 | 674 | 0.03 | [0.02; 0.05] | 0.5% |
| Tavares 2012 | 115 | 919 | 0.13 | [0.10; 0.15] | 0.5% |
| Bindt 2013 | 76 | 717 | 0.11 | [0.08; 0.13] | 0.5% |
| Makara-Studzinska 2013 (1) | 40 | 314 | 0.13 | [0.09; 0.17] | 0.5% |
| Makara-Studzinska 2013 (2) | 34 | 314 | 0.11 | [0.08; 0.15] | 0.5% |
| Makara-Studzinska 2013 (3) | 39 | 314 | 0.12 | [0.09; 0.17] | 0.5% |
| Enatescu 2014 | 40 | 80 | 0.50 | [0.39; 0.61] | 0.5% |
| Nguyen 2015 (1) | 10 | 211 | 0.05 | [0.02; 0.09] | 0.5% |
| Nguyen 2015 (2) | 5 | 211 | 0.02 | [0.01; 0.05] | 0.4% |
| Abdelhai 2015 | 237 | 376 | 0.63 | [0.58; 0.68] | 0.5% |
| Abdelhai 2015 (1) | 49 | 67 | 0.73 | [0.61; 0.83] | 0.5% |
| Abdelhai 2015 (2) | 51 | 77 | 0.66 | [0.55; 0.77] | 0.5% |
| Abdelhai 2015 (3) | 137 | 232 | 0.59 | [0.52; 0.65] | 0.5% |
| Chen 2016 (1) | 5 | 195 | 0.03 | [0.01; 0.06] | 0.4% |
| Chen 2016 (2) | 5 | 190 | 0.03 | [0.01; 0.06] | 0.4% |
| Chen 2016 (3) | 6 | 186 | 0.03 | [0.01; 0.07] | 0.5% |
| Chen 2016 (4) | 7 | 179 | 0.04 | [0.02; 0.08] | 0.5% |
| Chen 2016 (5) | 3 | 153 | 0.02 | [0.00; 0.06] | 0.4% |
| Dikmen-Yildiz 2017 (1) | 104 | 950 | 0.11 | [0.09; 0.13] | 0.5% |
| Dikmen-Yildiz 2017 (2) | 161 | 858 | 0.19 | [0.16; 0.22] | 0.5% |
| Dikmen-Yildiz 2017 (3) | 74 | 829 | 0.09 | [0.07; 0.11] | 0.5% |
| Nasreen 2018 | 72 | 904 | 0.08 | [0.06; 0.10] | 0.5% |
| Redinger 2018 | 68 | 936 | 0.07 | [0.06; 0.09] | 0.5% |
| Nakic Radoš 2018 | 42 | 272 | 0.15 | [0.11; 0.20] | 0.5% |
| González-Hernández 2019 | 26 | 188 | 0.14 | [0.09; 0.20] | 0.5% |
| Gelman 2019 | 141 | 298 | 0.47 | [0.42; 0.53] | 0.5% |
| Uguz 2019 | 36 | 1119 | 0.03 | [0.02; 0.04] | 0.5% |
| Wassif 2019 | 106 | 500 | 0.21 | [0.18; 0.25] | 0.5% |
| Redinger 2020 (1) | 49 | 649 | 0.08 | [0.06; 0.10] | 0.5% |
| Redinger 2020 (2) | 63 | 649 | 0.10 | [0.08; 0.12] | 0.5% |
| González-Mesa 2020 (1) | 113 | 250 | 0.45 | [0.39; 0.52] | 0.5% |
| Pabon 2020 | 85 | 531 | 0.16 | [0.13; 0.19] | 0.5% |
| Coo 2021 (1) | 9 | 148 | 0.06 | [0.03; 0.11] | 0.5% |
| Coo 2021 (2) | 13 | 148 | 0.09 | [0.05; 0.15] | 0.5% |
| Coo 2021 (3) | 17 | 148 | 0.11 | [0.07; 0.18] | 0.5% |
| Dong 2021 | 13 | 156 | 0.08 | [0.05; 0.14] | 0.5% |
| Zhou 2021 | 75 | 813 | 0.09 | [0.07; 0.11] | 0.5% |
| Cheng 2021 (1) | 39 | 156 | 0.25 | [0.18; 0.33] | 0.5% |
| Cheng 2021 (2) | 42 | 147 | 0.29 | [0.21; 0.37] | 0.5% |
| Cheng 2021 (3) | 40 | 129 | 0.31 | [0.23; 0.40] | 0.5% |
| Cheng 2021 (4) | 27 | 83 | 0.33 | [0.23; 0.44] | 0.5% |
| Luo 2021 | 142 | 2237 | 0.06 | [0.05; 0.07] | 0.5% |
| Nasreen 2021 | 9 | 566 | 0.02 | [0.01; 0.03] | 0.5% |
| Zhou 2022 | 51 | 210 | 0.24 | [0.19; 0.31] | 0.5% |
| Esquivel 2022 (1) | 16 | 274 | 0.06 | [0.03; 0.09] | 0.5% |
| Esquivel 2022 (2) | 9 | 261 | 0.03 | [0.02; 0.06] | 0.5% |
| Esquivel 2022 (3) | 8 | 243 | 0.03 | [0.01; 0.06] | 0.5% |
| Ma 2022 | 142 | 1583 | 0.09 | [0.08; 0.10] | 0.5% |
| Yang 2023 | 365 | 1963 | 0.19 | [0.17; 0.20] | 0.5% |
| Yang 2023 | 39 | 457 | 0.09 | [0.06; 0.11] | 0.5% |
| Hou 2023 (1) | 252 | 1082 | 0.23 | [0.21; 0.26] | 0.5% |
| Hou 2023 (2) | 50 | 926 | 0.05 | [0.04; 0.07] | 0.5% |
| Hou 2023 (3) | 35 | 757 | 0.05 | [0.03; 0.06] | 0.5% |
| Hou 2023 (4) | 49 | 685 | 0.07 | [0.05; 0.09] | 0.5% |
| Chen 2024 | 234 | 648 | 0.36 | [0.32; 0.40] | 0.5% |
| Shen 2024(1) | 304 | 1941 | 0.16 | [0.14; 0.17] | 0.5% |
| Shen 2024(2) | 157 | 1878 | 0.08 | [0.07; 0.10] | 0.5% |
| Shen 2024(3) | 226 | 1941 | 0.12 | [0.10; 0.13] | 0.5% |
| Shen 2024(4) | 252 | 1901 | 0.13 | [0.12; 0.15] | 0.5% |
| Zheng 2024 (1) | 190 | 2876 | 0.07 | [0.06; 0.08] | 0.5% |
| Zheng 2024 (2) | 305 | 2876 | 0.11 | [0.10; 0.12] | 0.5% |
| Zheng 2024 (3) | 103 | 2876 | 0.04 | [0.03; 0.04] | 0.5% |
| Zheng 2024 (4) | 159 | 2876 | 0.06 | [0.05; 0.06] | 0.5% |
| Jin 2024 | 1 | 549 | 0.00 | [0.00; 0.01] | 0.3% |
| Ma 2024 | 20 | 80 | 0.25 | [0.16; 0.36] | 0.5% |
| **Random effects model** | | **50518** | **0.11** | **[0.09; 0.13]** | **36.6%** |
| Heterogeneity: $I^2 = 98.7\%$, $\tau^2 = 0.8123$, $p = 0$ | | | | | |
| **Random effects model** | | **560736** | **0.09** | **[0.08; 0.10]** | **100.0%** |
| Heterogeneity: $I^2 = 99.5\%$, $\tau^2 = 0.8939$, $p = 0$ | | | | | |
| Test for subgroup differences: $\chi^2_2 = 4.94$, df = 2 ($p = 0.0848$) | | | | | |
