# Supplementary material for: Prevalence of co-morbid anxiety and depression in pregnancy and postpartum: a systematic review and meta-analysis
Source: Psychol Med. 2025 Mar 13;55:e84. doi: 10.1017/S0033291725000601 (PMC12080659; doi:10.1017/S0033291725000601)
Supplement: Ou et al. supplementary material 4 — Ou et al. supplementary material [file S0033291725000601sup004.pdf]

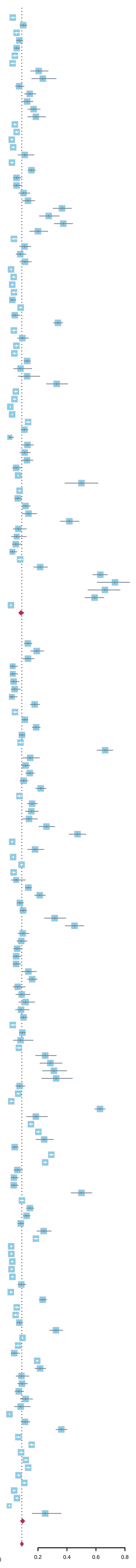

Study	Events	Total	Proportion	95%−CI	Weight
**Year = Before2016**					
Sutter−Dallay 2004	13	497	0.03	[0.01; 0.04]	0.5%
Adewuya 2005 (1)	62	632	0.10	[0.08; 0.12]	0.5%
Adewuya 2005 (2)	33	630	0.05	[0.04; 0.07]	0.5%
Adewuya 2005 (3)	43	600	0.07	[0.05; 0.10]	0.5%
Adewuya 2005 (4)	29	547	0.05	[0.04; 0.08]	0.5%
Adewuya 2005 (5)	21	512	0.04	[0.03; 0.06]	0.5%
Adewuya 2005 (6)	12	480	0.02	[0.01; 0.04]	0.5%
Andersson 2006 (1)	39	190	0.21	[0.15; 0.27]	0.5%
Andersson 2006 (2)	25	107	0.23	[0.16; 0.33]	0.5%
Miller 2006	23	325	0.07	[0.05; 0.10]	0.5%
Lee 2007 (1)	48	335	0.14	[0.11; 0.19]	0.5%
Lee 2007 (2)	42	335	0.13	[0.09; 0.17]	0.5%
Lee 2007 (3)	57	335	0.17	[0.13; 0.21]	0.5%
Phillips 2007	31	167	0.19	[0.13; 0.25]	0.5%
Sato 2008 (1)	55	1348	0.04	[0.03; 0.05]	0.5%
Sato 2008 (2)	72	1348	0.05	[0.04; 0.07]	0.5%
Sato 2008 (3)	26	1348	0.02	[0.01; 0.03]	0.5%
Sato 2008 (4)	39	1348	0.03	[0.02; 0.04]	0.5%
Rowe 2008	15	138	0.11	[0.06; 0.17]	0.5%
Reck 2008	21	1024	0.02	[0.01; 0.03]	0.5%
Faisal−Cury 2009	129	831	0.16	[0.13; 0.18]	0.5%
Ali 2009 (1)	14	267	0.05	[0.03; 0.09]	0.5%
Ali 2009 (2)	14	267	0.05	[0.03; 0.09]	0.5%
Ali 2009 (3)	27	267	0.10	[0.07; 0.14]	0.5%
Ali 2009 (4)	35	267	0.13	[0.09; 0.18]	0.5%
Giakoumakigiako 2009 (1)	86	235	0.37	[0.30; 0.43]	0.5%
Giakoumakigiako 2009 (2)	48	175	0.27	[0.21; 0.35]	0.5%
Giakoumakigiako 2009 (3)	88	235	0.37	[0.31; 0.44]	0.5%
Giakoumakigiako 2009 (4)	35	175	0.20	[0.14; 0.27]	0.5%
Qiao 2009	18	527	0.03	[0.02; 0.05]	0.5%
Teixeira 2009 (1)	29	270	0.11	[0.07; 0.15]	0.5%
Teixeira 2009 (2)	21	270	0.08	[0.05; 0.12]	0.5%
Teixeira 2009 (3)	30	270	0.11	[0.08; 0.15]	0.5%
Batenburg−Eddes 2009	75	5296	0.01	[0.01; 0.02]	0.5%
Woolhouse 2009	45	1385	0.03	[0.02; 0.04]	0.5%
Austin 2010	29	1289	0.02	[0.02; 0.03]	0.5%
Goodman 2010 (1)	17	491	0.03	[0.02; 0.05]	0.5%
Goodman 2010 (2)	7	299	0.02	[0.01; 0.05]	0.5%
Yelland 2010	344	4269	0.08	[0.07; 0.09]	0.5%
Fisher 2010	8	199	0.04	[0.02; 0.08]	0.5%
Field 2010	308	911	0.34	[0.31; 0.37]	0.5%
Edhborg 2011	23	674	0.03	[0.02; 0.05]	0.5%
Figueiredo 2011	24	260	0.09	[0.06; 0.13]	0.5%
Le Strat 2011	78	1524	0.05	[0.04; 0.06]	0.5%
Paul 2012	42	1123	0.04	[0.03; 0.05]	0.5%
Tavares 2012	115	919	0.13	[0.10; 0.15]	0.5%
Grant 2012 (1)	7	88	0.08	[0.03; 0.16]	0.5%
Grant 2012 (2)	11	88	0.12	[0.06; 0.21]	0.5%
Ali 2012	55	167	0.33	[0.26; 0.41]	0.5%
Storksen 2012	78	1642	0.05	[0.04; 0.06]	0.5%
Bener 2012	63	1659	0.04	[0.03; 0.05]	0.5%
Ban 2012 (1)	1083	116457	0.01	[0.01; 0.01]	0.5%
Ban 2012 (2)	2539	116457	0.02	[0.02; 0.02]	0.5%
Ibanez 2012	227	1719	0.13	[0.12; 0.15]	0.5%
Bindt 2013	76	717	0.11	[0.08; 0.13]	0.5%
Wynter 2013	1	172	0.01	[0.00; 0.03]	0.3%
Makara−Studzinska 2013 (1)	40	314	0.13	[0.09; 0.17]	0.5%
Makara−Studzinska 2013 (2)	34	314	0.11	[0.08; 0.15]	0.5%
Makara−Studzinska 2013 (3)	39	314	0.12	[0.09; 0.17]	0.5%
Thiagayson 2013	10	200	0.05	[0.02; 0.09]	0.5%
Bjørk 2014	5510	87468	0.06	[0.06; 0.06]	0.5%
Enatescu 2014	40	80	0.50	[0.39; 0.61]	0.5%
Farr 2014	327	4451	0.07	[0.07; 0.08]	0.5%
Miller 2014	28	461	0.06	[0.04; 0.09]	0.5%
Koutra 2014	50	438	0.11	[0.09; 0.15]	0.5%
Segre 2014	27	200	0.14	[0.09; 0.19]	0.5%
Simpson 2014	100	240	0.42	[0.35; 0.48]	0.5%
Tendais 2014 (1)	9	139	0.06	[0.03; 0.12]	0.5%
Tendais 2014 (2)	5	94	0.05	[0.02; 0.12]	0.4%
Nguyen 2015 (1)	10	211	0.05	[0.02; 0.09]	0.5%
Nguyen 2015 (2)	5	211	0.02	[0.01; 0.05]	0.4%
Cheng 2015	82	1062	0.08	[0.06; 0.09]	0.5%
Martini 2015	65	302	0.22	[0.17; 0.27]	0.5%
Abdelhai 2015	237	376	0.63	[0.58; 0.68]	0.5%
Abdelhai 2015 (1)	49	67	0.73	[0.61; 0.83]	0.5%
Abdelhai 2015 (2)	51	77	0.66	[0.55; 0.77]	0.5%
Abdelhai 2015 (3)	137	232	0.59	[0.52; 0.65]	0.5%
Waldie 2015	78	5659	0.01	[0.01; 0.02]	0.5%
**Random effects model**		**377017**	**0.08**	**[0.07; 0.10]**	**40.4%**
Heterogeneity: I^2 = 99.6%, τ^2 = 0.9857, p = 0					
**Year = 2016−2024**					
Falah−Hassani 2016	87	667	0.13	[0.11; 0.16]	0.5%
Tham 2016 (1)	60	313	0.19	[0.15; 0.24]	0.5%
Tham 2016 (2)	41	313	0.13	[0.10; 0.17]	0.5%
Chen 2016 (1)	5	195	0.03	[0.01; 0.06]	0.4%
Chen 2016 (2)	5	190	0.03	[0.01; 0.06]	0.4%
Chen 2016 (3)	6	186	0.03	[0.01; 0.07]	0.5%
Chen 2016 (4)	7	179	0.04	[0.02; 0.08]	0.5%
Chen 2016 (5)	3	153	0.02	[0.00; 0.06]	0.4%
Venkatesh 2016 (1)	102	576	0.18	[0.15; 0.21]	0.5%
Venkatesh 2016 (2)	24	576	0.04	[0.03; 0.06]	0.5%
Dikmen−Yildiz 2017 (1)	104	950	0.11	[0.09; 0.13]	0.5%
Dikmen−Yildiz 2017 (2)	161	858	0.19	[0.16; 0.22]	0.5%
Dikmen−Yildiz 2017 (3)	74	829	0.09	[0.07; 0.11]	0.5%
Nasreen 2018	72	904	0.08	[0.06; 0.10]	0.5%
Odinka 2018	205	309	0.66	[0.61; 0.72]	0.5%
Aryal 2018 (1)	24	164	0.15	[0.10; 0.21]	0.5%
Aryal 2018 (2)	65	567	0.11	[0.09; 0.14]	0.5%
Dennis 2018 (1)	81	564	0.14	[0.12; 0.18]	0.5%
Dennis 2018 (2)	49	483	0.10	[0.08; 0.13]	0.5%
Dennis 2018	120	549	0.22	[0.18; 0.26]	0.5%
Redinger 2018	68	936	0.07	[0.06; 0.09]	0.5%
Suzuki 2018	75	470	0.16	[0.13; 0.20]	0.5%
Nakic Radoš 2018	42	272	0.15	[0.11; 0.20]	0.5%
González−Hernández 2019	26	188	0.14	[0.09; 0.20]	0.5%
Agbaje 2019	69	267	0.26	[0.21; 0.32]	0.5%
Gelman 2019	141	298	0.47	[0.42; 0.53]	0.5%
Aris−Meijer 2019	85	3842	0.02	[0.02; 0.03]	0.5%
Ngocho 2019	36	200	0.18	[0.13; 0.24]	0.5%
Tay 2019 (1)	151	5239	0.03	[0.02; 0.03]	0.5%
Tay 2019 (2)	455	5239	0.09	[0.08; 0.09]	0.5%
Uguz 2019	36	1119	0.03	[0.02; 0.04]	0.5%
Genovez 2019	5	100	0.05	[0.02; 0.11]	0.4%
Ramakrishna 2019	143	1070	0.13	[0.11; 0.16]	0.5%
Wassif 2019	106	500	0.21	[0.18; 0.25]	0.5%
Redinger 2020 (1)	49	649	0.08	[0.06; 0.10]	0.5%
Redinger 2020 (2)	63	649	0.10	[0.08; 0.12]	0.5%
Umuziga 2020	52	165	0.32	[0.25; 0.39]	0.5%
González−Mesa 2020 (1)	113	250	0.45	[0.39; 0.52]	0.5%
González−Mesa 2020 (2)	25	263	0.10	[0.06; 0.14]	0.5%
Premji 2020 (1)	24	282	0.09	[0.06; 0.12]	0.5%
Premji 2020 (2)	16	282	0.06	[0.03; 0.09]	0.5%
Premji 2020 (3)	14	282	0.05	[0.03; 0.08]	0.5%
Premji 2020 (4)	14	282	0.05	[0.03; 0.08]	0.5%
Kantipudi 2020	28	209	0.13	[0.09; 0.19]	0.5%
Pabon 2020	85	531	0.16	[0.13; 0.19]	0.5%
Coo 2021 (1)	9	148	0.06	[0.03; 0.11]	0.5%
Coo 2021 (2)	13	148	0.09	[0.05; 0.15]	0.5%
Coo 2021 (3)	17	148	0.11	[0.07; 0.18]	0.5%
Dong 2021	13	156	0.08	[0.05; 0.14]	0.5%
Bante 2021	67	667	0.10	[0.08; 0.13]	0.5%
Jha 2021	12	457	0.03	[0.01; 0.05]	0.5%
Zhou 2021	75	813	0.09	[0.07; 0.11]	0.5%
Arshad 2021	6	75	0.08	[0.03; 0.17]	0.5%
Cena 2021	64	934	0.07	[0.05; 0.09]	0.5%
Cheng 2021 (1)	39	156	0.25	[0.18; 0.33]	0.5%
Cheng 2021 (2)	42	147	0.29	[0.21; 0.37]	0.5%
Cheng 2021 (3)	40	129	0.31	[0.23; 0.40]	0.5%
Cheng 2021 (4)	27	83	0.33	[0.23; 0.44]	0.5%
Lilliecreutz 2021	22	299	0.07	[0.05; 0.11]	0.5%
Luo 2021	142	2237	0.06	[0.05; 0.07]	0.5%
Nasreen 2021	9	566	0.02	[0.01; 0.03]	0.5%
Stampini1 2021	464	739	0.63	[0.59; 0.66]	0.5%
Ray 2022	23	124	0.19	[0.12; 0.27]	0.5%
Mateus 2022 (1)	504	3326	0.15	[0.14; 0.16]	0.5%
Mateus 2022 (2)	799	3939	0.20	[0.19; 0.22]	0.5%
Zhou 2022	51	210	0.24	[0.19; 0.31]	0.5%
Abdus−salam 2022	15	380	0.04	[0.02; 0.06]	0.5%
Motrico 2022	980	3356	0.29	[0.28; 0.31]	0.5%
Avalos 2022	1559	6236	0.25	[0.24; 0.26]	0.5%
Esquivel 2022 (1)	16	274	0.06	[0.03; 0.09]	0.5%
Esquivel 2022 (2)	9	261	0.03	[0.02; 0.06]	0.5%
Esquivel 2022 (3)	8	243	0.03	[0.01; 0.06]	0.5%
IRUM 2022	100	200	0.50	[0.43; 0.57]	0.5%
Ma 2022	142	1583	0.09	[0.08; 0.10]	0.5%
Malaju 2022 (1)	112	775	0.14	[0.12; 0.17]	0.5%
Malaju 2022 (2)	94	775	0.12	[0.10; 0.15]	0.5%
Malaju 2022 (3)	63	775	0.08	[0.06; 0.10]	0.5%
Odinka 2022	74	309	0.24	[0.19; 0.29]	0.5%
Yang 2023	365	1963	0.19	[0.17; 0.20]	0.5%
Hannon 2023 (1)	27	1804	0.01	[0.01; 0.02]	0.5%
Hannon 2023 (2)	29	1804	0.02	[0.01; 0.02]	0.5%
Hannon 2023 (3)	41	1804	0.02	[0.02; 0.03]	0.5%
Hannon 2023 (4)	32	1804	0.02	[0.01; 0.02]	0.5%
Hannon 2023 (5)	43	1804	0.02	[0.02; 0.03]	0.5%
Yang 2023	39	457	0.09	[0.06; 0.11]	0.5%
Craemer 2023	9	717	0.01	[0.01; 0.02]	0.5%
Hou 2023 (1)	252	1082	0.23	[0.21; 0.26]	0.5%
Hou 2023 (2)	50	926	0.05	[0.04; 0.07]	0.5%
Hou 2023 (3)	35	757	0.05	[0.03; 0.06]	0.5%
Hou 2023 (4)	49	685	0.07	[0.05; 0.09]	0.5%
Tambawala 2023	142	438	0.32	[0.28; 0.37]	0.5%
Bertram 2023	4638	49598	0.09	[0.09; 0.10]	0.5%
Dadhwal 2023	43	680	0.06	[0.05; 0.08]	0.5%
Gennaro 2023	7	194	0.04	[0.01; 0.07]	0.5%
Costa 2024	2303	11809	0.20	[0.19; 0.20]	0.5%
Fijean 2024	109	508	0.21	[0.18; 0.25]	0.5%
Altendahl 2024(1)	15	174	0.09	[0.05; 0.14]	0.5%
Altendahl 2024(2)	27	301	0.09	[0.06; 0.13]	0.5%
Altendahl 2024(3)	21	311	0.07	[0.04; 0.10]	0.5%
Altendahl 2024(4)	28	243	0.12	[0.08; 0.16]	0.5%
Eriksson 2024	9	112	0.08	[0.04; 0.15]	0.5%
Bain 2024	62	18218	0.00	[0.00; 0.00]	0.5%
Bante 2024	50	449	0.11	[0.08; 0.14]	0.5%
Chen 2024	234	648	0.36	[0.32; 0.40]	0.5%
Haile 2024	56	858	0.07	[0.05; 0.08]	0.5%
Shen 2024(1)	304	1941	0.16	[0.14; 0.17]	0.5%
Shen 2024(2)	157	1878	0.08	[0.07; 0.10]	0.5%
Shen 2024(3)	226	1941	0.12	[0.10; 0.13]	0.5%
Shen 2024(4)	252	1901	0.13	[0.12; 0.15]	0.5%
Zheng 2024 (1)	190	2876	0.07	[0.06; 0.08]	0.5%
Zheng 2024 (2)	305	2876	0.11	[0.10; 0.12]	0.5%
Zheng 2024 (3)	103	2876	0.04	[0.03; 0.04]	0.5%
Zheng 2024 (4)	159	2876	0.06	[0.05; 0.06]	0.5%
Jin 2024	1	549	0.00	[0.00; 0.01]	0.3%
Ma 2024	20	80	0.25	[0.16; 0.36]	0.5%
**Random effects model**		**183719**	**0.09**	**[0.08; 0.11]**	**59.6%**
Heterogeneity: I^2 = 99.1%, τ^2 = 0.8314, p = 0					
**Random effects model**		**560736**	**0.09**	**[0.08; 0.10]**	**100.0%**
Heterogeneity: I^2 = 99.5%, τ^2 = 0.8939, p = 0					
Test for subgroup differences: χ^2_1 = 0.70, df = 1 (p = 0.4037)					
